# Randomised Clinical Trial Investigating the Specificity of a Novel Skin Test (C-Tb) for Diagnosis of *M. tuberculosis* Infection

**DOI:** 10.1371/journal.pone.0064215

**Published:** 2013-05-14

**Authors:** Henrik Aggerbeck, Rafaela Giemza, Paulatsya Joshi, Pernille N. Tingskov, Søren T. Hoff, Julia Boyle, Peter Andersen, David J. M. Lewis

**Affiliations:** 1 Department of Vaccine Development, Statens Serum Institut, Copenhagen, Denmark; 2 Clinical Research Centre, Faculty of Health and Medical Sciences, University of Surrey Guildford, Guildford, United Kingdom; 3 Centre for Infection, St George’s University of London, London, United Kingdom; 4 Department of Infectious Disease Immunology, Statens Serum Institut, Copenhagen, Denmark; National Institute for Infectious Diseases (L. Spallanzani), Italy

## Abstract

**Background:**

Tuberculin skin testing is simple and relatively inexpensive, but the specificity of PPD is affected by BCG vaccination.

**Objective:**

Determine optimal dose and specificity of recombinant ESAT-6 and CFP-10 (C-Tb) produced in *Lactococcus lactis* for diagnosis of *M. tuberculosis* infection.

**Methods:**

In a dose finding phase I trial 0.01 or 0.1 µg preserved and unpreserved C-Tb was injected by Mantoux technique in 38 patients with active tuberculosis and induration responses measured. In a phase II specificity trial in 151 uninfected, BCG vaccinated participants 0.1 µg C-Tb was compared to 2 TU PPD.

**Results:**

0.1 µg C-Tb gave a median induration of 15 mm after 2 days. Phenol preservation did not affect the response. The specificity of C-Tb was 99.3% (95% CI 96–100%) regarding indurations ≥5 mm as a positive outcome. This was higher than the specificity of PPD (63% using a cut-off of 5 mm or 92% using a cut-off of 15 mm to adjust for non-specific BCG responses). Local adverse reactions following C-Tb injection included transient itching and discomfort as expected components of the immune response.

**Conclusion:**

C-Tb offers a simple and convenient skin test to diagnose *M. tuberculosis* infection using a single, universal cut-off unaffected by BCG vaccination.

**Trial Registration:**

ClinicalTrials.gov NCT01033929 and NCT01241188.

## Introduction

Tuberculosis (TB) constitutes an enormous global burden with 8.7 million new cases and an estimated 1.4 million deaths in 2011 [1]. Pulmonary TB disease develops in between 5 to10% of exposed individuals within 1–2 years after exposure [2], [3], and is defined by *Mycobacterium tuberculosis* (MTB) positive samples from sputum or infected tissues [4], [5]. Asymptomatic, latent TB infection may reactivate at a later stage, particularly if the individual acquires immunodeficiency from HIV. In low prevalence countries a predominant goal is to detect and treat latent TB in order to achieve the stage of TB elimination. This includes testing high risk groups such as close contacts of an active TB case, HIV-infected, and children below 5 years of age [6], [7]. There is no gold standard for the diagnosis of latent TB. Many guidelines include Tuberculin Purified Protein Derivative (PPD) and the interferon-γ release assays (IGRAs) QuantiFERON®-TB Gold In Tube (QFT-IT, Cellestis, Carnegie, Australia) and T-SPOT®.*TB* (Oxford Immunotec, Abingdon, UK) for the detection of latent TB, either alone or in combination [4], [8], [9].

Tuberculin skin testing (TST) has been used for a century [10]. It is a “low tech” procedure resulting in an induration typically 10–20 mm in diameter at the injection site of an MTB infected patient [11]–[13]. TST can be delivered and interpreted by point-of-care medical, nursing or paramedical staff, has no need for a laboratory, with a sensitivity around 75–90% [11], [13]. Reactivity to PPD may appear around 6 weeks after exposure to MTB [14]. PPDs main disadvantage is its cross-reactivity with environmental mycobacteria and all BCG vaccine strains. To compensate for this cut-off points between 5 and 15 mm of induration are recommended in various guidelines [6], [15]. However, if infected with HIV a low cut-off point may be used in spite of known BCG vaccination. This gives rise to a complex interpretation of PPD results with numerous local variations and clinical uncertainties.

The IGRAs are blood based *in vitro* assays based on peptides covering the MTB specific, BCG-deleted antigens ESAT-6 and CFP-10 giving specificities of 98–100% [16]. Due to costs and complexity the World Health Organization issued in 2011 a ‘negative’ policy statement with a caution against replacing TST by IGRAs as public health intervention to detect latent MTB infection in low- and middle-income settings [17]. As discussed in a recent workshop there is a need for an improved skin test [18]. C-Tb is a skin test for the diagnosis of MTB infection applied in the same way as PPD but using ESAT-6 and CFP-10 produced as recombinant proteins in *Lactococcus lactis*.

We report here (i) a phase I trial to determine an optimal dose of C-Tb and to compare unpreserved and phenol preserved C-Tb when testing patients with active tuberculosis, and (ii) a phase II trial to investigate the specificity of C-Tb in uninfected, BCG vaccinated participants which represent two key populations in which a TST must operate for clinical diagnosis of active or latent TB.

## Materials and Methods

### C-Tb

C-Tb was manufactured by SSI, Denmark according to Good Manufacturing Practice as a solution of recombinant ESAT-6 (dimer) and CFP-10 mixed in a weight ratio of 1∶1. Both antigens derived from *M. tuberculosis* were cloned and expressed in *Lactococcus lactis* [19]. For blinding purposes C-Tb were filled in identical 10 dose vials as PPD RT 23 SSI and appeared as indistinguishable clear, colourless solutions. C-Tb was injected by the Mantoux technique as previously described [20].

### QFT-IT

*In vitro* IFNγ responses were measured in blood samples prior to administration of C-Tb. An IFNγ response ≥0.35 IU/ml was regarded positive indicating MTB infection, negative <0.35 IU/ml. The result was indeterminate if the positive mitogen control minus blank was <0.5 IU/ml. Results above 10 IU/ml were reported as 10 IU/ml. In the specificity trial in healthy volunteers, additional blood samples for QFT-IT measurements were collected 28 days (and 6 months) after skin testing.

### Study Designs

The two double-blind studies described here were designed as within-subject paired comparisons in which two skin test agents were randomized in blocks of 10 or 12 to the left or right forearm of each participant. Vials of each agent were put in boxes numbered in succession and the volunteers were given intradermal injections of 0.1 mL by Mantoux technique in their order of appearance. In the dose-finding trial preserved (0.5% phenol) and unpreserved C-Tb (doses of 0.01 µg and 0.1 µg per 0.1 mL) were compared. In the specificity trial 0.1 µg C-Tb (preserved with 0.5% phenol) was compared with a standard 2 TU PPD RT 23 SSI dose.

The protocols for the two trials and supporting CONSORT checklist are available as supporting information; see Checklist S1 and Protocols S1 and S2.

### Dose-finding Trial

The dose-finding trial was conducted from February to November 2010 at the Centre for Infection at St. George’s University of London, United Kingdom and included 38 adults. The recruited patients were diagnosed with active TB with a compatible clinical picture of TB with the intention to treat (Table 1). Participants were recruited from outpatient clinics in the South West area of London within the first 2 month of acute phase of antibiotic treatment and included with a positive culture, PCR result, smear microscopy, or IGRA result. Treatments with products likely to modify the immune response (e.g. corticosteroids or blood products) or HIV infection were exclusion criteria. The patients were recruited in 3 groups of 12. The first group received 0.01 µg preserved and unpreserved C-Tb in either arm according to a randomization code. Based on an evaluation of safety and skin reactions the last two groups received the same dose of 0.1 µg. Follow-up visits took place after 1, 2, 3, 4, and 28 days and included measurements of indurations and erythema. Immediate pain caused by the injections was assessed by a Visual Analogue Scale (VAS) using a ruler (Schlenker Enterprises LTD). The ruler transformed various degrees of pain visualized by smileys into a scale from 0 mm (no pain) to 100 mm (worst possible). Blood samples for haematology and biochemistry analyses were collected at baseline screening within 28 days before skin testing and at last visit 21–32 days after testing.

**Table 1:** Distribution of primary site of tuberculosis infection and number of patients in dose-finding trial.

	C-Tb dose group (/0.1 mL)	
TB diagnose	0.01 µg	0.1 µg
Pulmonary	6 (50%)	13 (50%)
Lymph node	0 (0%)	6 (23%)
Pleural	2 (16%)	2 (8%)
Spinal	3 (25%)	0 (0%)
TB meningitis	0 (0%)	1 (4%)
Other^1^	1 (8%)	4 (15%)
Total	12	26

1 Including disseminated/miliary TB.

### Specificity Trial

The specificity trial was conducted from May to November 2011 at the Surrey Clinical Research Centre, Guildford, United Kingdom and included 151 healthy, BCG vaccinated adults. The most important exclusion criteria were history of TB or close contact to a person with active TB, a positive QFT-IT at inclusion, immunosuppressive treatment, congenital and/or acquired immune deficiency or pregnancy. C-Tb and PPD were injected by Mantoux technique and the diameter of any induration measured after 2–3 days. All systemic and local adverse events occurring within 28 days were recorded. Laboratory safety parameters of haematology and biochemistry and a 2^nd^ QFT-IT were performed on day 28. An IFNγ response occurring at this time point was regarded an immune reaction to C-Tb/PPD and an additional blood sample was collected after 6 months for a repeated measurement to investigate the duration of the response.

### Sample Size Calculation

The sample size of 12 (24) in the dose finding trial was mainly based on practical considerations. However, a group of 12 (24) volunteers gave 72% (92%) chance of discovering an unacceptable adverse reaction appearing at a true frequency of 10%.

The specificity was estimated as a relative frequency in a binomial distribution. With N patients the variance of the estimated specificity (û) is Var(û) = u(1–u)/N, where u is the (unknown) true specificity. For a fixed value of N Var(û) obtains its maximum when u = 0.5. With N = 150 this maximum is 0.25/N = 0.0017 corresponding to a standard deviation SD(û) of 0.041. As the true specificity is expected to be higher than 0.5 the standard deviation of 0.041 is a worst case scenario. With a more realistic value for the true specificity of 0.8 the corresponding standard deviation becomes 0.033, which was considered an acceptable precision of the estimate.

### Data Safety Monitoring Board

A Data Safety Monitoring Board consisting of four independent senior clinicians was established for the dose-finding trial to evaluate adverse reactions prior to proceeding to the next strength of C-Tb.

### Ethics Statement

The trials were conducted according to “Note for guidance on good clinical practice”, CPMP/ICH/135/95, ICH topic E6 and CPMP/ICH/377/95 E2A and in accordance with the ethical principles of the edition of the Declaration of Helsinki, adopted at the 59th World Medical Association General Assembly, Seoul, in October 2008. For both trials written informed consent was obtained prior to inclusion. The dose-finding trial was approved by The Royal Marsden Research Ethics Committee (No 09/H0801/90) and The Medicines and Healthcare Products Regulatory Agency (MHRA; No. 17282/0204/001-0001). The specificity trial was approved by the NRES Committee London – East & City (No 10/H0703/109) and by MHRA (No 17282/0205/001-0001). The trials were filed in the NIH clinical trials database NCT01033929 and NCT01241188.

## Results

**Figure 1:**
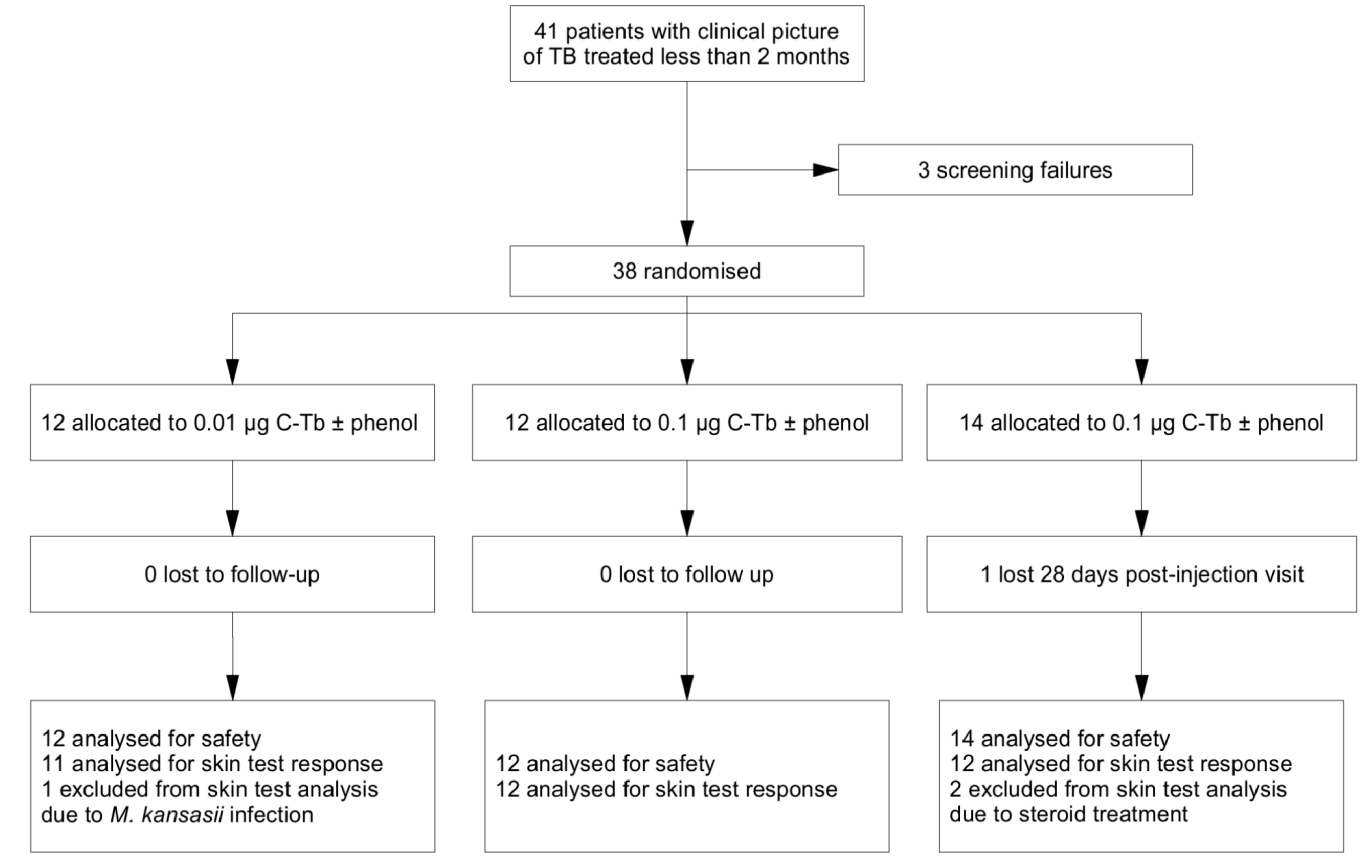
Flow diagram of dose-finding trial. TB patients received C-Tb (unpreserved and phenol preserved) in either arm. 3/11 (0.01 µg C-Tb) and 13/24 (0.1 µg C-Tb) had a culture or PCR confirmed TB at inclusion, the rest were smear (2/11 and 2/24) or IGRA (6/11 and 9/24) positive.

The dose-finding trial included 38 TB patients (23 males) with mean age of 33 years (18–60). All patients were included in the safety analyses and 35 patients were included in analysis of skin test reactions (Figure 1). 16 patients (46%) had a culture or PCR confirmed TB at inclusion, 4 (11%) were included based on a positive smear, 8 (23%) on a positive T-SPOT®.*TB* and 7 (20%) on a positive QFT-IT. One patient retrospectively failed the inclusion criteria as sputum culture revealed *M. kansasii* instead of *M. tuberculosis* infection. This patient showed no response to 0.01 µg C-Tb. Two patients were inappropriately enrolled due to immune suppressive medication (steroid treatment) and were subsequently replaced. 20 patients (53%) had a history of BCG vaccination and 18 (47%) had received a TST 7–80 days prior to the study as part of the TB diagnostic process.

**Figure 2:**
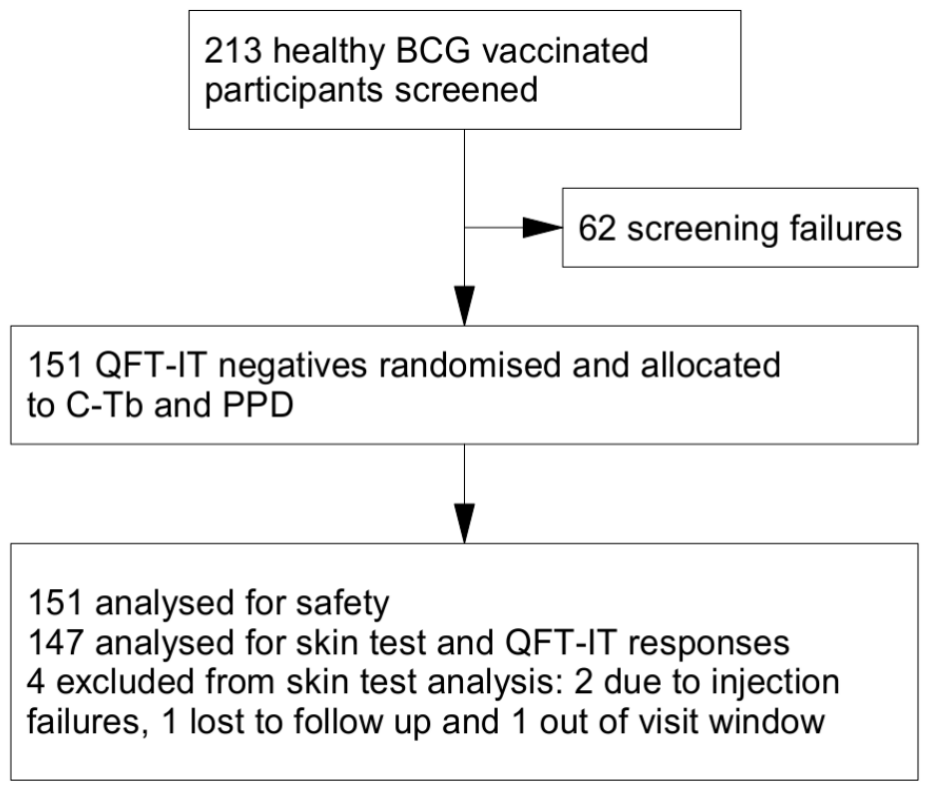
Flow diagram of specificity trial. BCG vaccinated, healthy, QFT-IT negative participants received 0.1 µg C-Tb and 2 TU PPD RT 23 SSI in either arm.

The specificity trial included 151 healthy, BCG vaccinated participants (59 males) with a mean age of 34 years (18–65). 135 were classified as white, 4 as black, 9 as Asian, and 3 as other. All patients were included in the safety analyses and 147 were included in analysis of skin test reactions (Figure 2).

### Protocol Deviations

In the dose finding trial the pain associated with the injection was assessed 1 h after the administration answering the question “How painful did you experience the injections?” scored according to the VAS scale. 16 patients in the 0.1 µg dose group were in error asked “How painful do you experience the pain now after 1 h?”.

### Dose Response of C-Tb in TB Patients

**Figure 3:**
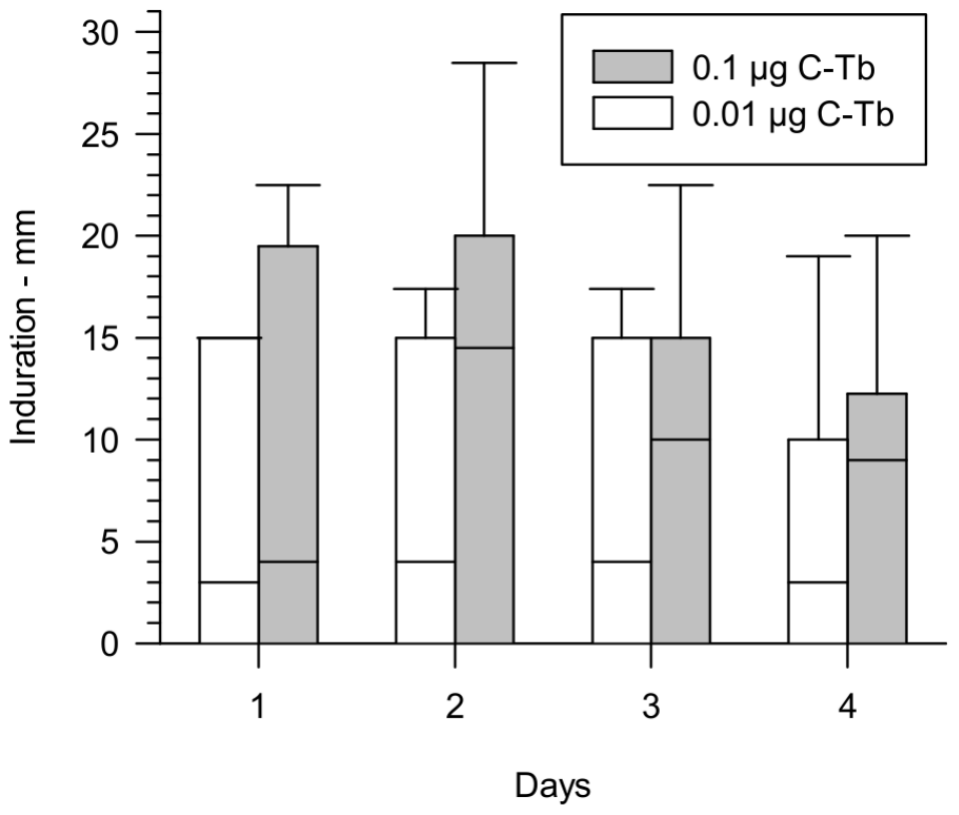
Time course of the induration response to preserved C-Tb in patients with active TB. The median induration response (line within boxes) is shown for the first 4 days after injection of TB patients with 0.01 µg (white; N = 11) or 0.1 µg C-Tb (grey; N = 24) in dose-finding trial. The boundaries of boxes are 25^th^ and 75^th^ percentiles. Error bar indicate 90^th^ percentile.

The induration response peaked after 2 days with a median response of 14.5 mm (0.1 µg C-Tb, Figure 3). The responses remained visible at day 4. TST performed prior to the present trial in 18 of the tuberculosis patients showed a median response of 15 mm (5–22 mm). The median responses with 0.01 µg C-Tb were below 5 mm. A dose of 0.1 µg C-Tb was chosen for succeeding trials to give a response similar in size to the one of PPD. Maximum responses with C-Tb ranged between 4–23 mm (0.01 µg) and 10–40 mm (0.1 µg). No skin reactions were apparent after 1 month in either dose group.

### Effect of Phenol Preservation on C-Tb in TB Patients

**Figure 4:**
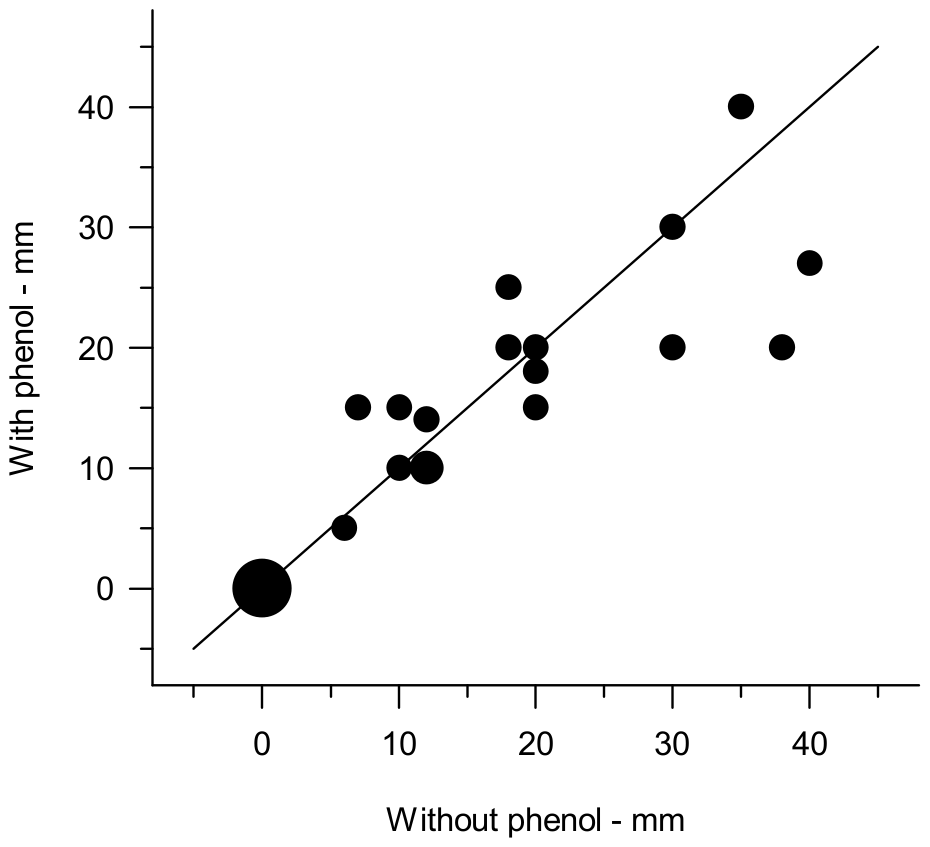
Induration 2 days after injection of unpreserved and phenol preserved C-Tb (0.1 µg) to TB patients. Line of identity is shown. Area of dot indicates number of patients with dot at (0.0) representing 7 participants. N = 24.

The size of induration was not affected by phenol (Figure 4; Wilcoxon’s Signed Rank: P>0.05). Phenol (0.5%) preservation of C-Tb did not influence the immediate pain associated with the injection. Thus, 10/22 patients experienced no difference in VAS score between the left and the right arm. Among the remaining 12, five experienced less pain with the phenol preserved formulation. The pain seemed to disappear within the first hour after the injection as reported by 13/16 patients.

### Comparison between C-Tb and QFT-IT Assay in TB Patients

**Figure 5:**
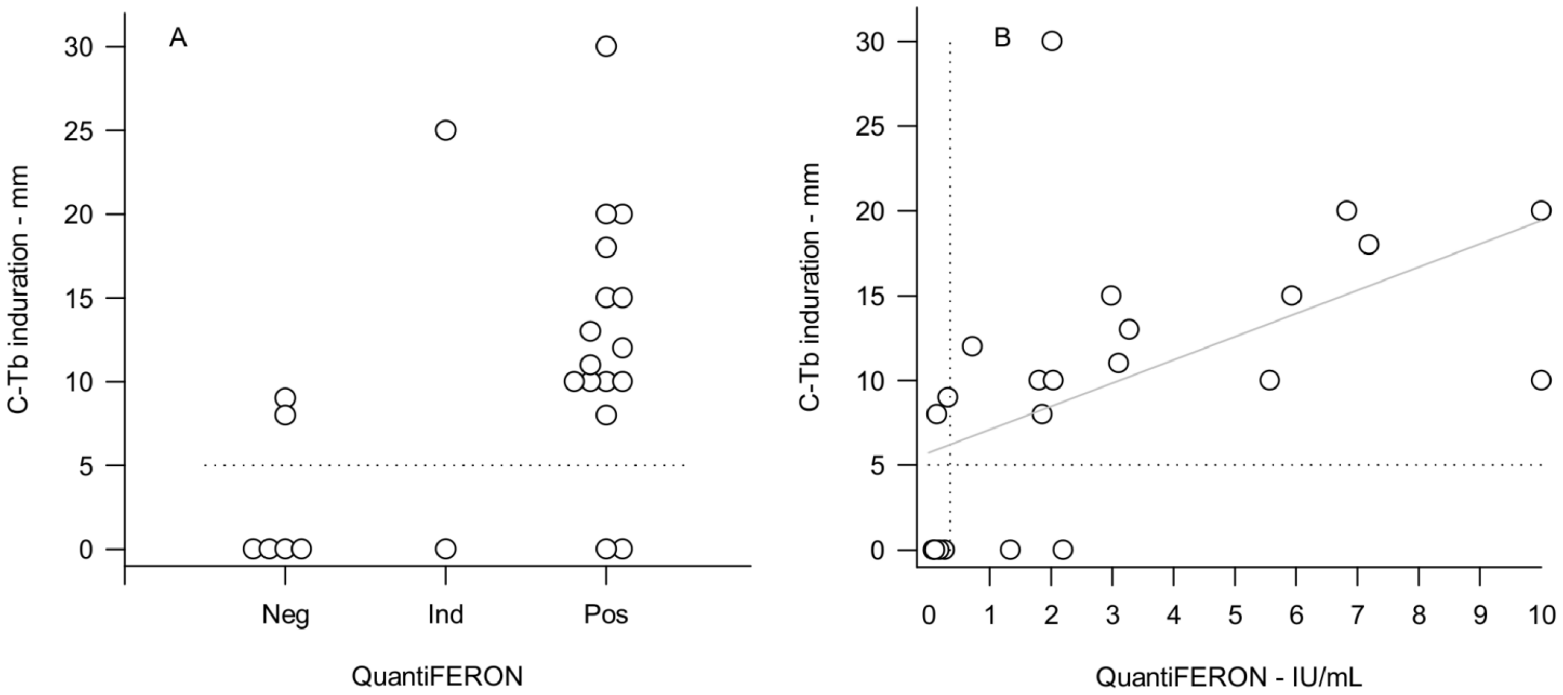
QFT-IT and C-Tb responses in patients with active TB. QFT-IT positive-negative result (panel A) and baseline concentration of IFNγ (panel B) at screening versus induration diameter 3 days after injection of 0.1 µg preserved C-Tb to TB patients (N = 24). Indeterminate (Ind) results are omitted in panel B. Dotted lines represents cut-off values. Regression line is shown in panel B.

In the dose finding trial C-Tb (0.1 µg) and QFT-IT showed concordant results in 18/22 patients (82%, Figure 5A). The magnitude of the responses were positively related (R = 0.54, Figure 5B) with a slope of the regression line of 1.4 mm·ml/IU significantly different from zero at the 5% level. In 3/22 cases inclusion was based on a positive QFT-IT result giving a bias if used in a comparison with C-Tb. In the remaining 19 cases where inclusion was based on other criteria, C-Tb and QFT-IT showed moderate agreement with concordant results in 15/19 cases (κ = 0.51). In four cases of lymph node (2) or pulmonary TB (2) the two test systems gave discordant results: two C-Tb positives (one lymph node, one pulmonary) tested negative with QFT-IT (0.13 IU/ml and 0.31 IU/ml), and two QFT-IT positives (1.33 IU/ml and 2.19 IU/ml) gave no induration with C-Tb. Three of those had culture confirmed TB, whereas one testing negative with C-Tb and positive with QFT-IT had a positive smear. The two indeterminate results with QFT-IT included with a positive T-SPOT®.*TB* had pleural or lymph node TB. Four patients tested negative with both tests: two included with a positive culture and two with a positive T-SPOT®.*TB*.

### Interaction between C-Tb/PPD and QFT-IT Assay in Unexposed, BCG-vaccinated Adults

To ensure a low risk of MTB infection all participants in the specificity trial were included as QFT-IT negative at entry (<0.35 IU/ml). At the end of the trial (26–30 days later) five had become QFT-IT positive and one had an indeterminate result (Table 2). Two of those had a baseline result of 0.024 and 0.064 IU/ml respectively, the rest were ≤0 IU/ml. In all five positives the responses were negative when retested after 6 months. Three of the five had an induration response to PPD of 9, 9 and 10 mm, but none had an induration response to C-Tb.

**Table 2:** IFNγ among responders to C-Tb/PPD 1 and 6 months after skin testing in specificity trial.

	QFT-IT (IU/ml)			Skin test (mm)	
Subject	Baseline	1 month	6 months	PPD RT 23	C-Tb
S565	0.064	0.746	0.119	0	0
S578	0.024	0.354	−0.021	10	0
S676	−0.042	13.911	−0.005	0	0
S700	−0.009	3.435	0.045	9	0
S702	0.000	0.832	0.000	9	0

### Specificity of C-Tb in Healthy BCG-vaccinated Participants

**Figure 6:**
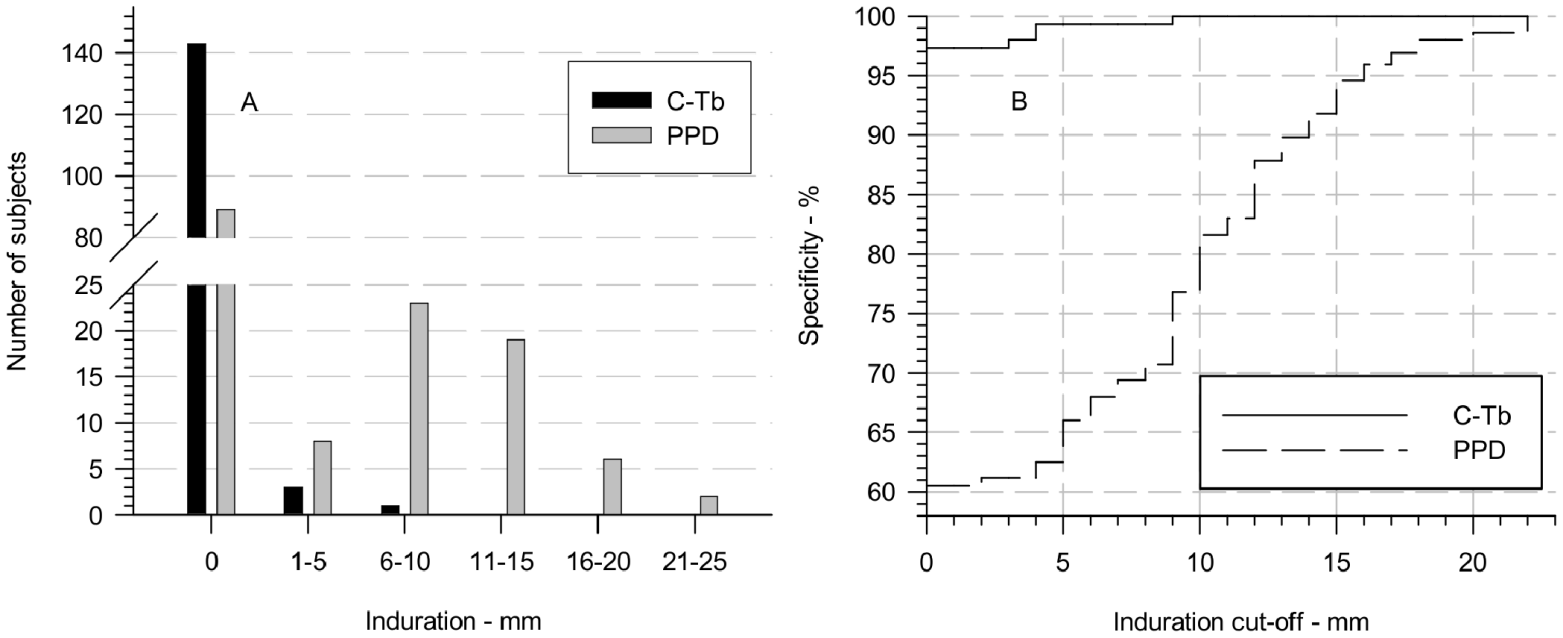
Distribution of induration responses and specificity of C-Tb and PPD in healthy BCG-vaccinated adults. The induration responses 2–3 days after testing with 0.1 µg C-Tb (black bars) and 2 TU PPD RT 23 SSI (grey bars) are shown in panel A. The resulting specificity of C-Tb (solid line) and PPD RT 23 SSI (broken line) as a function of cut-off is shown in panel B. N: 147.

The specificity of C-Tb and PPD was defined as the relative frequency of induration responses below a given cut-off measured two to three days after injection to participants with no known exposure to MTB. Of the 147 participants in the per-protocol population included with a negative QFT-IT result, 143 participants (97.3%) showed no induration to C-Tb (Figure 6 A). One participant had an induration of 3 mm, two of 4 mm, and one of 9 mm. None of the four participants showing an induration to C-Tb had a response to PPD and they all remained QFT-IT negative. An optimal cut-off for C-Tb will be based on the specificity trial reported here and a sensitivity trial in TB patients. Preliminary results indicate the cut-off of C-Tb to be 5 mm. However, any induration will correspond to a specificity of 97.3% (95% CI 93.2–99.3%) and 5 mm to a specificity of 99.3% (95% CI 96.3–100.0%; Figure 6B) for C-Tb. The specificity of PPD was 62.6% [95% CI 54.2–70.4%] using a cut-off of 5 mm, increasing to 76.9% [69.2–83.4%] and 91.8% [86.2–95.7%] using cut-offs of 10 and 15 mm respectively.

### Adverse Reactions to C-Tb in TB Patients

No C-Tb-related serious adverse events in tuberculosis patients occurred during the 28 days trial period. 31 of 38 experienced one or more adverse events of which the most common were itching and pain at the injection site (Table 3). These reactions were more frequent with the high dose of C-Tb.

**Table 3:** Local adverse reactions within 28 d after injection of unpreserved and phenol preserved C-Tb.

	0.01 µg C-Tb^1^		0.1 µg C-Tb^2^	
Local reaction	Unpreserved	Preserved	Unpreserved	Preserved
Itch	6 (50%)	3 (25%)	17 (65%)	16 (61%)
Pain	2 (17%)	1 (8%)	8 (31%)	6 (23%)

1 N = 12.

2 N = 26.

Patients in dose-finding trial were in treatment for active TB.

All injection site reactions categorised as at least possibly related to C-Tb recovered within 28 days except from hyperpigmentations reported by two patients. Systemic events with 0.01 µg C-Tb (12 patients tested) included moderate night sweats (1), moderate rash on neck (1), and ‘feeling feverish’(2). Systemic events with 0.1 µg C-Tb (26 patients tested) included ‘feeling feverish’ (2), headache (1). One patient reported rash/warm/itchy/slightly swollen/pain. All systemic events categorised as at least possibly related to C-Tb recovered within 28 days, and are also compatible with underlying tuberculosis and treatment. No clinically significantly abnormal laboratory results related to C-Tb were recorded.

### Adverse Reactions to C-Tb in Unexposed, BCG-vaccinated Adults

No serious adverse events or events of severe intensity occurred during the specificity trial. Among 151 participants 48 (32%) experienced an adverse injection site reaction at the C-Tb injection site, versus 31 (21%) at the PPD injection site (p = 0.036). All C-Tb adverse injection site reactions were of mild intensity.

Haematoma at the C-Tb site was the most common, accounting for 38 of the 48 reported adverse reactions. Seven participants (4.6%) reported a systemic adverse reaction assessed as possibly related to administration of the two skin tests. All systemic adverse events were of mild or moderate intensity. No clinically significantly abnormal laboratory results were recorded.

## Discussion

Using treated TB patients as surrogate for a latent infection we previously showed proof of principle for the C-Tb test using only rdESAT-6 [21]. It was found that 0.1 µg rdESAT-6 gave an induration similar in size to 2 TU PPD RT 23 SSI. To increase sensitivity rCFP-10 was subsequently included in C-Tb [22]. In a first-in-man trial C-Tb showed no signs of false positive reactions (no sensitisation) if healthy participants were tested twice 6 weeks apart corresponding to the incubation period of TB [20]. In MTB infected guinea pigs C-Tb showed the same dose response as previously described with rdESAT-6 (data not shown) [19]. Yet, as a safety precaution the initial dose of C-Tb was reduced to 0.01 µg in the dose finding trial described here because this study took place in TB patients with a risk of more adverse reactions and stronger responsiveness than in treated cases. PPD was not included as direct comparator as it might already have been applied during diagnostic workup giving a risk of a booster response [13]. However, indurations with 0.1 µg C-Tb were similar to those reported previously for PPD [12], [13]. When used in standard doses in patients with active TB, PPD may induce painful responses with ulceration. It was therefore reassuring that in this population of active TB cases, C-Tb was associated with few, minor adverse reactions, of which itching and pain were the most frequent and probably inevitable parts of the diagnostic immune response.

For optimal cost effectiveness C-Tb could be distributed in multi-dose vials, so C-Tb was preserved with 0.5% phenol to comply with the European Pharmacopoeia on antimicrobial efficacy. It is encouraging therefore that phenol did not seem to give rise to unspecific indurations or affect adverse reactions in patients with active tuberculosis tested with C-Tb. This concurs with two studies done in 2000 investigating unpreserved and phenol preserved PPD RT 23. The studies included 209 healthy participants and 55 TB patients in Lithuania and 319 healthy participants in Sweden. Similar immune responses were observed, but VAS scores showed more pronounced immediate pain with the unpreserved preparation (SSI unpublished data). In the present study the numbers were too low to verify a positive influence of phenol on the injection site pain. This may partly be due to the fact that 16/38 patients were asked a differently phrased question about the pain during the conduct of the trial.

In the specificity trial four of the 147 QFT-IT negative, healthy participants had an induration response with C-Tb. As there is no gold standard to identify latent TB it cannot be ruled out that some of the responders may be true C-Tb positives. The participant with a 9 mm C-Tb response worked as an ambulance technician, and although he did not give a specific history of contact with a known TB case, one could speculate that this occupation may expose him to an increased risk of contact with a contagious case of TB. Nonetheless, our data suggest a specificity of C-Tb of 99% at a cut-off 5 mm. This is similar to the specificities reported for the blood-based IGRAs [9], [16]. In comparison a specificity of 92% was seen with PPD RT 23 using a cut-off of 15 mm to compensate for non-specific contribution to the induration response by prior BCG vaccination. Because QFT-IT was part of the inclusion criteria to ensure a low risk of MTB infection, a direct comparison of C-Tb and QFT-IT was not possible due to bias.

Based on the prevalence of childhood BCG immunization different regions employ different cut-offs for PPD, so it is evident that use of a single cut-off for C-Tb irrespective of BCG status will simplify the interpretation of a skin reaction.

C-Tb and QFT-IT takes advantage of the same antigens, and the trials described here indicate a similar performance of the two tests. However, in four cases in the dose-finding study C-Tb and QFT-IT showed discordant results in patients confirmed by culture or smear. Discordance could not be explained by the site of infection as both groups were represented by one case of lymph node and one case of pulmonary TB. Part may be related to the definition of positive test result as one testing positive with C-Tb showed an IFNγ result of 0.31 IU/ml slightly below the cut-off for QFT-IT. Another reason could be difference in incubation time, overnight for QFT-IT vs. 2–3 days for C-Tb, which could affect the sensitivity. The degree of discordance between C-Tb and QFT-IT of 18% was of the same magnitude as previously reported for QFT-IT and T-SPOT®.*TB* [23]. Four TB patients tested negative with C-Tb and QFT-IT. Genetic factors may be involved in their lack of reactivity [24].

As skin testing and IGRAs may both be used in clinical practice we sought to investigate the effect of C-Tb and PPD injections on the QFT-IT. In the specificity trial five participants developed a temporary QFT-IT response. It was not possible to discriminate if this was due to boosting or a false positive reaction.

By combining the high specificity of the costly and technically complex IFNγ release assays with the low tech procedure of tuberculin and a single cut-off, C-Tb may become a valuable tool for the detection of MTB infection by point-of-care staff. Two phase III trials have been initiated to investigate the performance of C-Tb in groups who are known to be at higher risk of developing tuberculosis including close contacts to pulmonary TB cases, children below 5 years of age and HIV-infected.

## Supporting Information

Checklist S1 **CONSORT Checklist.**
(DOC)

Protocol S1 **Trial Protocol.**
(PDF)

Protocol S2 **Trial Protocol.**
(PDF)
