# Supplementary material for: Randomised Clinical Trial Investigating the Specificity of a Novel Skin Test (C-Tb) for Diagnosis of M. tuberculosis Infection
Source: PLoS One. 2013 May 14;8(5):e64215. doi: 10.1371/journal.pone.0064215 (PMC3653866; doi:10.1371/journal.pone.0064215)
Supplement: Protocol S1 — Trial Protocol. (PDF) [file pone.0064215.s002.pdf]

PROTOCOL

FINAL version

Title:

A safety and dose finding trial of the diagnostic test C-Tb, when given intradermally by the Mantoux technique to adult patients recently diagnosed with active TB

Trial Code: TESEC-02

EudraCT Number: 2009-012984-33

Trial phase: I

Date of this version:

02.10.2009

Study Director:

Pernille Nyholm Tingskov
Department of Vaccine Development
Statens Serum Institut
Denmark

Confidentiality:
This document contains confidential information.
The contents may not be used, divulged or published without prior written consent of Statens Serum Institut.
This information cannot be used for any other purpose than the conduct and evaluation
of the clinical investigation by the investigator(s), regulatory authorities and members of the ethics committee.

1 Synopsis

**Name of sponsor:**<br>Statens Serum Institut		
**Finished product:** C-Tb		
**Name of active ingredient:**<br>Recombinant dimer of 6 kDa early secretory antigen target (rdESAT-6) and recombinant 10 kDa culture filtrate protein (rCFP-10)		
**Title of the trial:**	A safety and dose finding trial of the diagnostic test C-Tb, when given intradermally by the Mantoux technique to adult patients recently diagnosed with active TB.	
**Investigator:**	Professor David JM. Lewis	
**Trial centre:**	Centre for Infection at St George's University of London, London, SW17 0RE, United Kingdom	
**Country:**	United Kingdom	
**Trial period:**	January 2010 (FPFV) to Q4 2010 (LPLV)	
**Trial code /<br>EUDRACT number:**	TESEC-02 / 2009-012984-33	
**Trial phase:**	Phase Ib	
**Primary objective:**	To assess the safety of two dose levels of C-Tb (0.01 and 0.1 µg/0.1 mL) when administered intradermally by the Mantoux technique to patients in the acute phase of treatment against active TB.	
**Secondary objectives:**	1. To assess the immune response of two doses (0.01 and 0.1 µg/0.1 mL) of C-Tb from the size of induration.<br>2. To assess the safety of unpreserved C-Tb and C-Tb preserved with 0.5% phenol.<br>3. To assess the pain associated with the injection of unpreserved C-Tb and C-Tb preserved with 0.5% phenol using the VAS scale.	
**Safety variables:**	1. Local adverse reactions at the injection sites within 28 days after application of the tests. Induration < 50 mm and erythema < 80 mm will be regarded as effect variables and should therefore not be categorized as local adverse events. However the occurrence of an induration ≥ 50 mm and/or erythema ≥ 80 mm will be regarded as undesired reactions to the investigational product and must be regarded as local adverse reactions.<br>2. All adverse events occurring within 28 days after application of the tests.	
**Immune response variables:**	The diameter of induration at the injection sites measured transversely to the long axis of the forearm at 24, 48, 72 and 96 hours after application of the agents.	

Trial design:

This clinical trial is a single centre phase Ib open dose adjustment study with respect to the dose of C-Tb combined with a double blind randomised, split-body comparison of unpreserved C-Tb and C-Tb preserved with 0.5% phenol (each patient receives the unpreserved version in one arm and the preserved version in the other arm).

The w:w ratio of rdESAT-6 and rCFP-10 (C-Tb) is 1:1 in all test solutions. For example a dose level of 0.01 µg C-Tb refers to a test solution consisting of 0.005 µg rdESAT-6 and 0.005 µg rCFP-10 per 0.1 mL.

Patients in treatment for active tuberculosis will be included in the trial in blocks of 12:

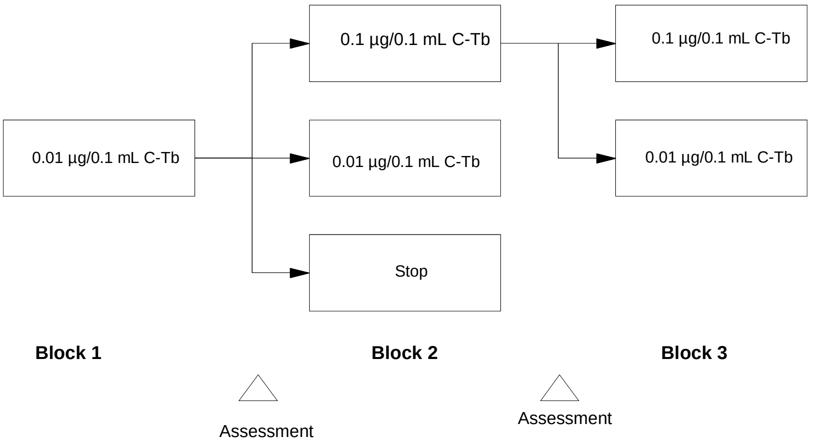

The first 12 patients (Block 1) will receive a low dose of 0.01 µg/0.1 mL C-Tb without phenol in the RIGHT or LEFT arm and 0.01 µg/0.1 mL C-Tb with phenol in the opposite arm, in a double blind manner. Follow-up visits will take place after 24, 48, 72, 96 hours and 28 days.

A safety and an immune response assessment done 96 hours after testing the last patient in Block 1 may lead to three different scenarios in Block 2 (i-iii):

i) If the 0.01 µg/0.1 mL C-Tb used in Block 1 is assessed NOT to be safe the trial will stop. Only 12 patients will have received 0.01 µg/0.1 mL C-Tb.

ii) If the 0.01 µg/0.1 mL C-Tb used in Block 1 is safe and gives an adequate immune response the next 12 patients (Block 2) will receive the same low dose of 0.01 µg/0.1 mL C-Tb with/without phenol in the RIGHT or LEFT arm. Follow-up visits will take place after 24, 48, 72, 96 hours and 28 days as described for Block 1. The trial will be terminated with 24 patients having received 0.01 µg/0.1 mL C-Tb.

iii) If the 0.01 µg/0.1 mL C-Tb is safe but does NOT give an adequate immune response the 12 patients allocated to Block 2 will receive a high dose of 0.1 µg/0.1 mL C-Tb with/without phenol in the RIGHT or LEFT arm. Follow-up visits will as described above take place after 24, 48, 72, 96 hours and 28 days.

A safety and an immune response assessment done 96 hours after testing the last patient in Block 2 scenario iii) may lead to two different scenarios in Block 3 (a-b):

	a) If the high dose of C-Tb is safe and gives an adequate immune response the last 12 patients (Block 3) will receive a high dose of 0.1 µg/0.1 mL C-Tb with/without phenol in the RIGHT or LEFT arm. Follow-up visits will take place after 24, 48, 72, 96 hours and 28 days as described previously. The trial will be terminated with a total number of 12 patients included to receive the low dose of 0.0.1 µg/0.1 mL C-Tb and 24 patients included to receive the high dose of 0.1 µg/0.1 mL C-Tb.<br><br>b) If the high dose of C-Tb gives an adequate immune response but is NOT safe the 12 patients allocated to Block 3 will receive a low dose of 0.01 µg/0.1 mL C-Tb with/without phenol in the RIGHT or LEFT arm. Follow-up visits will take place after 24, 48, 72, 96 hours and 28 days. The trial will be terminated with a total number of 24 patients having received the low dose of 0.01 µg/0.1 mL C-Tb and 12 patients having received the high dose of 0.1 µg/0.1 mL C-Tb.<br><br>The safety evaluation after administration of each dose level is performed by the principal investigator. A pre-defined Data Safety Monitoring Board of experts will be involved in the safety and the immune response assessments before deciding which of the above described scenarios to follow further on in the clinical trial.<br><br>The patients will be monitored closely for adverse events for 1 hour after administration of the skin tests (day 0). The following visits take place on days 1, 2, 3, 4, and 28.<br><br>For safety reasons there will be at least 1 hour between the injections of C-Tb to successive patients.<br><br>Digital photos of the injection sites are taken as part of the assessment at every visit. All injection site reactions and induration/erythema diameters are measured by experienced study staff according to standard operating procedures.<br><br>Diaries are given to the patients for the recording of adverse events and concomitant medication after the skin test administration. At Visits 3, 4, 5, 6 and 7 adverse events and concomitant medications are assessed and recorded in the CRF by the investigator/study nurse
**Trial population:**	Female/male adults (between 18 - 65 years of age) without HIV who are diagnosed and in treatment for acute TB infection.
**Number of patients:**	A total of 12, 24 or 36 patients allocated to either of two dose levels in blocks of 12.
**Inclusion criteria:**	**The patient:**<br>1. Has signed an informed consent<br>2. Is willing and likely to comply with the trial procedures<br>3. Has been diagnosed with active TB and has been in treatment ≤ 60 days at the time of inclusion<br>4. a) has 1 documented positive sputum smear microscopy result or<br>b) has positive culture or<br>c) has a positive PCR result for tuberculosis or<br>d) has a compatible clinical picture of TB with the intention to treat<br>5. Has a positive T-spot assay or a QuantiFERON®-TB Gold In Tube test<br>6. Is prepared to grant authorized persons access to their medical records

**Exclusion criteria:**	**The patient:**<br>1. Has been in treatment with a product which is likely to modify the immune response within 3 months prior to the day of inclusion (e.g., immunoglobulin, systemic corticosteroids, methotrexate, azathioprine, cyclosporine or blood products)<br>2. Has been vaccinated with a live vaccine within 6 weeks prior to the day of inclusion (e.g. BCG, MMR, yellow fever, oral typhoid vaccines)<br>3. Has a known congenital or acquired immune deficiency<br>4. Has a disease affecting the lymphoid organs (e.g., Hodgkin's disease, lymphoma, leukaemia, sarcoidosis)<br>5. Is infected with HIV<br>6. Has severe scarring, burn, rash, eczema, psoriasis, or any other skin disease at or near the injection sites<br>7. Has a condition where blood drawings pose more than minimal risk for the patient, such as haemophilia, other coagulation disorders, or significantly impaired venous access<br>8. Is actively participating in another clinical trial<br>9. Is pregnant according to urine pregnancy test at inclusion<br>10. Has a condition which in the opinion of the investigator is not suitable for participation in the study
**Investigational diagnostic test:**	**Investigational product C-Tb (rdESAT-6 + rCFP-10):**<br>rdESAT-6........ 0.05/0.5µg<br>rCFP-10 .... 0.05/0.5µg<br>Disodium hydrogen phosphate dihydrate........1.4 mg<br>Potassium dihydrogen phosphate........0.2 mg<br>Potassium chloride........0.2 mg<br>Sodium chloride........8.0 mg<br>Polysorbate 20 ....0.1 µL<br>Phenol........ 0.0/0.5 %<br>Water for injections .... up to 1 mL
**Dosage and route of administration:**	Each patient will in each arm receive one injection containing the same dose of C-Tb (0.01 µg/0.1 mL or 0.1 µg/0.1 mL) but either unpreserved or preserved with 0.5 % phenol according to a randomisation code.<br><br>The two injections of C-Tb will be administered intradermally by the Mantoux technique into the flexor surface of the RIGHT/LEFT forearm at the junction of the upper third with the lower two-thirds. A 1 ml syringe fitted with a short bevelled needle (insulin needle) is used for the injection of the test.
**Statistical methods:**	Descriptive methods.

2 Table of Contents

1 Synopsis....2
2 Table of Contents....6
3 List of Abbreviations and Definitions....9
4 Signature page....11
5 Relevant Addresses....12
6 Introduction....14
7 Trial objectives....16
7.1 Primary objectives....16
7.2 Secondary objectives....16
7.3 Safety variables....17
7.4 Immune response variables....17
8 Investigational Plan....17
8.1 Overall design....17
8.2 Study population....19
8.3 Number of subjects....19
8.4 Recruitment....20
8.5 Inclusion criteria....20
8.6 Exclusion criteria....20
8.6.1 Predetermined reasons for discontinuation....21
8.6.2 Permanent contraindications....21
8.6.3 Temporary contraindications....22
9 Investigational Products....22
9.1 Composition....22
9.2 Treatments administered....22
9.2.1 Doses and administration....23
9.2.2 Packaging and labelling....24
9.2.3 Storage information....24
9.2.4 Transport of trial vaccines....24
9.2.5 Randomisation procedure....25
9.2.6 Blinding and unblinding....26
9.2.7 Treatment compliance....26
9.2.8 Drug accountability....26

9.2.9 Precautions and overdosing 27
9.2.10 Concomitant medication 27
9.3 Investigational events 28
9.3.1 Safety assessments 29
9.3.2 Immune response assessments 29
9.3.3 Collection and handling of blood samples 30
9.3.4 Time schedule 31
10 Ethical Aspects 31
10.1 Payment of volunteers 32
10.2 Data Safety Monitoring Board....32
11 Adverse Events 33
11.1 Definitions and terms....33
11.2 Standard reporting of adverse events....34
11.3 Investigator's immediate reporting of serious adverse events to SSI 36
11.4 SSI's expedited reporting of SUSARs to CA and EC 36
11.5 Annual reporting of suspected serious adverse reactions to CA and EC....38
12 Data Management and Statistical Analysis 38
12.1 General considerations....38
12.2 Data management 38
12.3 Clean file procedures 39
12.4 Analysis populations....39
12.5 Statistical methods 39
12.6 Sample size determinations 40
12.7 Interim analysis....40
13 Good Clinical Practice considerations 40
13.1 Declaration of Helsinki....40
13.2 Subject information and informed consent....40
13.3 Independent ethics committee submission and approval....41
13.4 Competent authority submission and approval....41
13.5 Subject data protection 41
13.6 Investigator's responsibilities 42
13.7 Curricula vitae and log(s) of staff 42
13.8 Indemnity statement....43
13.9 Training 43

13.10 Monitoring 43
13.11 Audit and inspection 44
13.12 Definition and archiving of source data 44
13.13 Definition and archiving of essential documents 44
14 Agreement and financial settlement 45
15 Insurance 45
16 Confidentiality and disclosure 45
17 Changes to the protocol 46
18 References 47

Appendices

Appendix 1: Declaration of Helsinki

Appendix 2: Written Information

Appendix 3: Informed Consent Form

Appendix 4: Indemnity Statement

Appendix 5: CIOMS form

Appendix 6: Insurance certificate

3 List of Abbreviations and Definitions

ADR	Adverse Drug Reaction
AE	Adverse Event
AFB	Acid fast bacteria
ALP	Alkaline Phosphatase
ALT	Alanine aminotransferase
AR	Adverse Reaction
AST	Aspartate aminotransferase
BCG	Bacille Calmette-Guérin
CA	Competent Authority
CFP-10	10 kDa Culture Filtrate Protein
CI	Confidence Interval
CIOMS	Council for International Organizations of Medical Sciences
CRA	Clinical Research Associate
CRF	Case Record Form
CTA	Clinical Trial Application
C-Tb	rdESAT-6 + rCFP10
DOB	Date of Birth
DTH	Delayed Type Hypersensitivity
DSMB	Data safety monitoring board
DSMP	Data safety monitoring plan
EC	Ethics Committee
ESAT-6	6 kDa Early Secretory Antigenic Target
FPFV	First Patient First Visit
GCP	Good clinical practice
GMP	Good manufacturing practice
HIV	Human Immunodeficiency Virus
IB	Investigator’s Brochure
ICH-GCP	The International Conference on Harmonisation of Good Clinical Practice
IMPD	Investigational medicinal product dossier
IF	Investigator’s file
IFN-γ	Interferon gamma
LPLV	Last Patient Last Visit
MHRA	Medicines and Healthcare products Regulatory Agency
MTb	Mycobacterium tuberculosis
NCS	Not clinically significant

Ph. Eur	The European Pharmacopoeia
PPD	Purified Protein Derivative
PPD RT 23	Purified Protein Derivative Rinsed Tuberculin (batch) 23
QFT	QuantiFERON®-TB Gold In Tube test
QP	Qualified Person
rCFP-10	Recombinant 10kDa Culture Filtrate Protein
rdESAT-6	Recombinant dimer of 6 kDa Early Secreted Antigen Target
RBC	Red blood cell count
REC	Research Ethics Committee
RGN	Registered General Nurse
SAE	Serious Adverse Event
Sensitivity	The probability that a test result is positive given the subject has the disease [33]
SGUL	St George’s, University of London
SPC	Summary of Product Characteristics
Specificity	The probability that a test result is negative given the subject does not have the disease [33]
SSI	Statens Serum Institut, Denmark
SSAR	Serious Suspected Adverse Reaction
SAR	Suspected adverse (drug) reaction
SUSAR	Suspected Unexpected Serious Adverse (drug) Reaction
TB	Tuberculosis
TDL	The Doctor’s Laboratory
TMF	Trial master file
TST	Tuberculin Skin Test
VAS	Visual Analogue Scale
WBC	White blood cell count
WHO	World Health Organisation

4 Signature page

Study Director, SSI:	Pernille Nyholm Tingskov	
		____________________ (date & signature)
Principal Investigator:	Prof. David JM. Lewis	
		____________________ (date & signature)
Project Manager:	Henrik Aggerbeck	
		____________________ (date & signature)
Trial statistician, SSI:	Jens Henrik Badsberg	
		____________________ (date & signature)
Medically responsible, SSI:	Trine R. Nielsen	
		____________________ (date & signature)
Sponsor's representative, SSI:	Ingrid Kromann	
		____________________ (date & signature)

5 Relevant Addresses

Principal Investigator:	Prof. David JM Lewis, MD<br>Infectious Diseases<br>St George's, Cranmer Terrace<br>London, SW17 0RE,<br>United Kingdom	Tel:<br>Fax:<br>e-mail:	+44 20 87255826<br>+44 20 87253487<br>sgjf300@sgul.ac.uk
Coordinating Nurse:	Ms Rafaela Giemza RGN<br>Infectious Diseases<br>St George's, Cranmer Terrace<br>London, SW17 0RE,<br>United Kingdom	Tel:<br>Fax:<br>e-mail:	+44 20 87252316<br>+44 20 87250170<br>rgiemza@sgul.ac.uk
Project Manager, SSI:	Henrik Aggerbeck<br>Statens Serum Institut<br>Department of<br>Vaccine Development<br>5 Artillerivej<br>DK-2300 Copenhagen S	Tel:<br>Fax:<br>e-mail:	+45 3268 3486<br>+45 3268 3872<br>hea@ssi.dk
Monitor, SSI:	Christine Dam Bendiksen<br>Statens Serum Institut<br>Department of<br>Vaccine Development<br>5 Artillerivej<br>DK-2300 Copenhagen S	Tel:<br>Fax:<br>e-mail:	+45 3268 3846<br>+45 3268 3872<br>cdb@ssi.dk
Study Director, SSI:	Pernille Tingskov<br>Statens Serum Institut<br>Department of<br>Vaccine Development<br>5 Artillerivej<br>DK-2300 Copenhagen S	Tel:<br>Fax:<br>e-mail:	+45 3268 3416<br>+45 3268 3872<br>pnt@ssi.dk
Medically responsible, SSI:	Trine R. Nielsen<br>Statens Serum Institut<br>Department of Regulatory and<br>Medical Affairs<br>5 Artillerivej<br>DK-2300 Copenhagen S	Tel:<br>Fax:<br>e-mail:	+45 3268 3436<br>+45 3268 3973<br>trn@ssi.dk

Sponsor's representative, SSI:	Ingrid Kromann<br>Statens Serum Institut<br>Department of Vaccine Development<br>5 Artillerivej<br>DK-2300 Copenhagen S	Tel: +45 3268 8252<br>Fax: +45 3268 3872<br>e-mail: ikr@ssi.dk
Trial statistician, SSI:	Jens Henrik Badsberg<br>Statens Serum Institut<br>Biostatistics Unit<br>5 Artillerivej<br>DK-2300 Copenhagen S	Tel: +45 3268<br>Fax: +45 3268 3886<br>e-mail: jhb@ssi.dk

Emergency telephone numbers and e-mail addresses:

Study Director, SSI:	Pernille Nyholm Tingskov	Office: +45 3268 3416<br>Mobile: +45 5176 1222<br>Private: +45 3537 8720
Project Manager, SSI:	Henrik Aggerbeck	Office: +45 3268 3486<br>Mobile: +45<br>Private: +45
Monitor, SSI:	Christine Dam Bendiksen	Office: +45 3268 3846<br>Mobile: +45 2030 3263<br>Private: +45 3534 2636
Medically responsible, SSI:	Trine R. Nielsen	Office: +45 3268 3436<br>Mobile: +45 2680 3003
Serious Adverse Event Notification to SSI:		Fax: +45 3268 3973<br>Mobile: +45 2567 0914<br>e-mail: clin.trial@ssi.dk

6 Introduction

Tuberculosis continues to be a major cause of morbidity and mortality throughout the world. The disease is caused by infection with *Mycobacterium tuberculosis* (MTb), an intracellular pathogen with one of the most devastating global impacts. WHO estimates that 9.3 million new cases of tuberculosis occurred in 2007 with an estimated 1.3 million deaths in HIV negative people and an additional 456 000 deaths among HIV-positive people [1].

One of the 'Millennium Development Goals' from the United Nations Development Programme [2] is to halt and begin to reverse the incidence of TB by 2015. One of the critical issues in achieving this goal is to control the disease by preventing infection and treating the disease as early as possible [3]. Fast and accurate diagnostics has a very important role in this; however in many clinical situations this is not possible as the existing diagnostic methods have serious limitations [5]. Worldwide the majority of TB patients are still diagnosed with tools developed in the 19th Century such as the Tuberculin Skin Test (TST) using Purified Protein Derivative (PPD) and sputum microscopy [3, 6, 7]. The drawback of PPD is the fact that its protein components are known to be shared by many non-tuberculous/environmental mycobacterium families commonly found in developing countries, as well as by the BCG vaccine strains. This significantly decreases the specificity of the TST, since individuals exposed to non-tuberculous mycobacteria or vaccinated with BCG respond immunologically to PPD as well as those who are infected with the tuberculous families (*M. tuberculosis, M. bovis, or M. africanum*) [8]. MTb recovered from human specimens by microbacteriel culture is today still the gold standard for diagnosing active TB, however the growth of MTb may take from 2 weeks to 2 months and clinicians therefore need to rely on other methods to diagnose patients and initialize treatment [3, 4].
Weaknesses of today's diagnostic tools lead to treatment of patients not infected with MTb, waste of resources and complications from the side affects in anti TB treatment [3]. The development of robust diagnostic tests for detecting latent and active TB is therefore urgently needed.

It has been discovered that the tuberculous bacteria contains a number of specific proteins/antigens that are not present in BCG or most environmental mycobacteria. The ESAT-6 (6 kD Early Secreted Antigen Target) protein and CFP-10 (10 kDa culture filtrate protein) were identified from a *M. tuberculosis* culture filtrate [9, 10]. Both antigens, ESAT-6 and CFP-10 are expressed by the tuberculous mycobacterium families (*M. tuberculosis, M. bovis, and M. africanum*) but not by any of the BCG-strains and by few of the atypical mycobacteria (e.g., *M. kansasii*, *M. marinum*, *M. szulgai*) [8, 10].

Based on this knowledge new in-vitro diagnostic tests have been developed in the form of T-cell based interferon-γ-assays. These assays use *Mycobacterium tuberculosis* - specific antigens (e.g. ESAT-6, CFP-10) and show that T-cells of individuals infected with *M. tuberculosis* produce interferon-γ when they are exposed to these antigens [11]. The tests show very promising abilities in detecting latent infection with *M. tuberculosis* and were shown to be able to discriminate between patients infected by *M. tuberculosis* and BCG vaccinated individuals [10, 12, 13, 14, 15, 24, 25].

The impact of these assays in resource limited countries will however be small due to cost, complexity and the lack of local laboratory infrastructure. [3].

A new tuberculin like skin test named C-Tb, using the rdESAT-6 and rCFP-10 antigens, has been developed as a diagnostic test for detecting *M. tuberculosis* infection in man, by SSI, Denmark, and has shown to induce local delayed type hypersensitivity (DTH) skin reactions when injected intradermally to guinea pigs infected with *M. tuberculosis* [10, 16, 17].

In 2005/2006 the rdESAT-6 reagent (alone) synthesised in *Lactococcus lactis*, was administered to humans for the first time in a phase Ia clinical trial conducted at Leiden University Medical College, the Netherlands. In the trial the skin test was given at 3 different doses (0.01 µg, 0.1 µg and 1 µg) to a group of previously treated TB-patients with a simultaneously administered tuberculin skin test in the opposite arm. The response due to the immunity induced by a cleared infection was investigated.
In the group of treated TB patients 0.1 µg rdESAT-6 induced a skin induration similar in size to that of 2 T.U. Tuberculin (PPD RT 23 SSI). The response to 0.01 µg rdESAT-6 was slightly lower and the response to 1.0 µg rdESAT-6 was slightly higher than the response to PPD. Local adverse reactions were seen in the groups of 0.01µg and 0.1µg rdESAT-6. When increasing the dose to 1µg, responses were seen in most volunteers and there was a higher frequency and intensity of local adverse reactions. There were also general complaints in this group e.g. headache, however, with uncertain relationship to the trial product. [20, 21].

In March 2007, SSI decided to include rCFP-10 to increase the sensitivity of the skin test reagent without jeopardizing its specificity [10]. Non-clinical pharmacology trials at SSI demonstrated in guinea pigs that rCFP-10 mixed with rdESAT-6 in the ratio 1:1 was optimal. The product was named C-Tb.

Reece et al [18] recently suggested that CFP-10 may trigger “Tuberculin shock”. He found that 5/10 guinea pigs died 6-36 hours after the skin testing with CFP-10, if the test was done 6 weeks after infection. No deaths were observed if the skin testing was done after 4 weeks of infection or earlier. A more likely explanation is that the testing and the deaths were coincidental as the guinea pigs after 6 weeks of infection were at a late stage of disease. In a repeat study, No. F1137 [19], done at SSI with an infection period of 6 weeks before testing 3/30 (10%) animals died or were about to die from tuberculosis even before skin testing. The same number of animals died after skin testing with rCFP-10 or PPD. It is likely that any immunological reagent at this time of disease will be able to induce a shock-like syndrome in the animals.

Non-clinical toxicity studies performed in 2008 concluded that repeated subcutaneous injections of 10µg C-Tb were safe in rats.

In 2008/2009 SSI conducted a phase Ia clinical trial at Rigshospitalet, Copenhagen investigating the safety and the risk of sensitisation after two repeated injections of 0.01 µg or 0.1 µg C-Tb in healthy adults. Few mild adverse events were reported in this first in man clinical trial with C-Tb.

Individuals with active TB may give a stronger response than treated TB patients and the aim of the present phase Ib clinical trial is to determine a safe human dose of C-Tb, which induces a distinct induration in truly TB infected patients.

It is expected that the patients only will be subjected to minimal risks of local and systemic adverse reactions following the intradermal injection of two simultaneous doses of either 0.01 µg or 0.1 µg C-Tb by the Mantoux injection technique. This is based on the non-clinical pharmacology and toxicity studies and two phase I clinical trials with rdESAT-6 (alone) [20, 21, 22, 23] and one phase I trial with C-Tb.

It is required that C-Tb is preserved if marketed in multi-dose vials. To compare the safety of unpreserved and preserved C-Tb each patient will in the present study receive two injections of C-Tb, one in each arm of either unpreserved or preserved with 0.5% phenol. A phenol concentration of 0.5% has been found adequate according to the Ph. Eur. test for the efficacy of antimicrobial preservation. In (unpublished) SSI clinical trials in Sweden and Lithuania 4 different formulations of 2 T.U. Tuberkulin PPD were investigated (unpreserved or preserved with 0.01% chinosol, 0.3% phenol, 0.5% phenol). The studies showed that the preservatives tested did not affect the immune response (induration). However, the phenol preserved formulations were less irritative than the unpreserved and the chinosol preserved formulations. The trials comprised 583 volunteers including 55 patients with TB.

Based on the above studies regarding the use of a phenol concentration of 0.5% it is not expected that the preserved formulation of C-Tb poses an additional risk for the patients.

This protocol has been written in accordance with ICH GCP Topic E 6 [26], ICH Topic E 2A [27] and the EC directives 2001/20/EC [28] and 2005/28/EC [29] of the European Parliament (including underlying guidance).

7 Trial objectives

7.1 Primary objectives

- To assess the safety of two doses of C-Tb (0.01 and 0.1 µg/0.1 mL) when administered intradermally by the Mantoux technique to patients in the acute phase of treatment against active TB.

7.2 Secondary objectives

- To assess the immune response of two doses (0.01 and 0.1 µg/0.1 mL) of C-Tb from the size of induration.

- To assess the safety of unpreserved C-Tb and C-Tb preserved with 0.5% phenol (local reactions).

- To assess the pain associated with the injection of unpreserved C-Tb and C-Tb preserved with 0.5% phenol using the VAS scale.

7.3 Safety variables

- Local adverse reactions at the injection sites within 28 days after application of the tests

  Induration < 50 mm and erythema < 80 mm will be regarded as effect variables and should therefore not be categorized as local adverse events. However the occurrence of an induration ≥ 50 mm and/or erythema ≥ 80 mm will be regarded as undesired reactions to the investigational product and must be regarded as local adverse reactions.

- All adverse events occurring within 28 days after application of the tests

7.4 Immune response variables

- The diameter of induration at the injection sites measured transversely to the long axis of the forearm at 24, 48, 72 and 96 hours after application of the agents.

8 Investigational Plan

8.1 Overall design

This clinical trial is a single centre phase Ib open dose adjustment study with respect to the dose of C-Tb combined with a double blind randomised, split-body comparison of unpreserved C-Tb and C-Tb preserved with 0.5% phenol (each patient receives the unpreserved version in one arm and the preserved version in the other arm).

The trial will be conducted at the Centre for Infection at St George's, University of London, Cranmer Terrace, London, SW17 0RE, United Kingdom, under the responsibility of principal investigator: Professor David JM Lewis.

The primary objective of the trial is to assess the safety of C-Tb at two different doses of 0.01 and 0.1 µg when given to patients in treatment for an active TB infection. In addition, the immune response of the two doses of C-Tb will be evaluated together with the assessment of the safety of unpreserved C-Tb and C-Tb preserved with 0.5% phenol.

Patients in treatment for active tuberculosis will be included in the trial in blocks of 12 as follows:

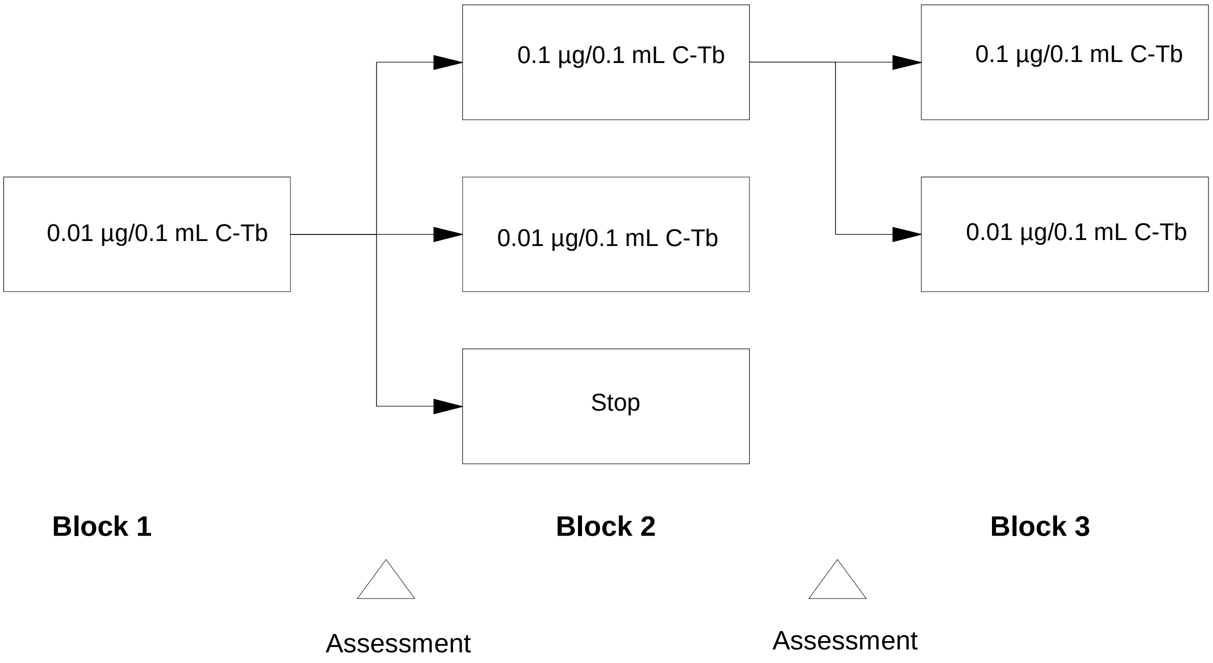

The first 12 patients (Block 1) will receive 0.01 µg/0.1 mL C-Tb without phenol in the RIGHT or LEFT arm and 0.01 µg/0.1 mL C-Tb with phenol in the opposite arm, in a double blind manner. Follow-up visits will take place after 24, 48, 72, 96 hours and 28 days.

A safety and immune response assessment done 96 hours after testing the last patient in Block 1 may lead to three different scenarios in Block 2 (i-iii):

i) If the 0.01 µg/0.1 mL C-Tb used in Block 1 is assessed NOT to be safe the trial will stop. Only 12 patients will have received 0.01 µg/0.1 mL C-Tb.

ii) If the 0.01 µg/0.1 mL C-Tb used in Block 1 is safe and gives an adequate immune response the next 12 patients (Block 2) will receive the same dose of 0.01 µg/0.1 mL C-Tb with/without phenol in the RIGHT or LEFT arm. Follow-up visits will take place after 24, 48, 72, 96 hours and 28 days as described for Block 1. The trial will then be terminated with 24 patients having received 0.01 µg/0.1 mL C-Tb.

iii) If the 0.01 µg/0.1 mL C-Tb is safe but does NOT give an adequate immune response the 12 patients allocated to Block 2 will receive 0.1 µg/0.1 mL C-Tb with/without phenol in the RIGHT or LEFT arm. Follow-up visits will as described above take place after 24, 48, 72, 96 hours and 28 days.

A safety and an immune response assessment done 96 hours after testing the last patient in Block 2 scenario iii) may lead to two different scenarios in Block 3 (a-b):

a) If the 0.1 µg/0.1 mL dose of C-Tb is safe and gives an adequate immune response the last 12 patients (Block 3) will receive the same dose with/without phenol in the RIGHT or LEFT arm. Follow-up visits will take place after 24, 48, 72, 96 hours and 28 days as described previously. The trial will then be terminated with 12 patients having received the low dose of 0.0.1 µg/0.1 mL C-Tb and 24 patients having received the high dose of 0.1 µg/0.1 mL C-Tb.

b) If the high dose of C-Tb gives an adequate immune response but is NOT safe the 12 patients allocated to Block 3 will receive 0.01 µg/0.1 mL C-Tb with/without phenol in the RIGHT or LEFT arm. Follow-up visits will take place after 24, 48, 72, 96 hours and 28 days. The trial will then be terminated with 24 patients having received the low dose of 0.01 µg/0.1 mL C-Tb and 12 patients having received the high dose of 0.1 µg/0.1 mL C-Tb.

The safety evaluation after administration of each dose level is performed by the principal investigator. A pre-defined Data Safety Monitoring Board of experts will be involved in the safety and the immune response assessments before deciding which of the above described scenarios to follow in the trial.

Each trial patient will be followed 28 days after application of the test products and is expected to complete a total of 7 trial visits, as follows:

**Visit 1**	-	Screening Visit
**Visit 2**	**Day 0**	Inclusion Visit and Injection of skin tests (0-28 days after screening)
**Visit 3**	**Day 1**	24-hour Assessment Visit (± 4 hours)
**Visit 4**	**Day 2**	48-hour Assessment Visit (± 4 hours)
**Visit 5**	**Day 3**	72-hour Assessment Visit (± 4 hours)
**Visit 6**	**Day 4**	96-hour Assessment Visit (± 4 hours)
**Visit 7**	**Day 28**	Final 28 day Visit (performed at St George’s) (± 2 days)

To increase compliance the trial nurse(s) of the TESEC-02 may perform the 24, 48, 72 and 96 hour-assessment visits at the patient’s home. The screening, inclusion and final visits (visit 1, 2 and 7) will, however, for safety reasons always take place at the Centre for Infection, St George's, University of London. For practical reasons the Day 0 visits should always take place on Mondays.

8.2 Study population

Female and male adults (between 18 - 65 years of age) without HIV who are newly diagnosed and in treatment for acute TB infection.

8.3 Number of subjects

A total of 12, 24 or 36 adult patients allocated to either of the two doses in blocks of 12.

8.4 Recruitment

Recruitment will primarily take place in TB centres in the South West area of London both from patients newly diagnosed and treated for TB and from the pool of patients already in TB treatment (≤ 60 days at the time of inclusion) and followed in the clinics. Posters will be placed in the clinic areas. In case of low recruitment from these clinics the area of recruitment may be expanded. The outpatient clinics will briefly inform the patients of the TESEC-02 trial and if patients give their consent they will be referred to the trial. The outpatient clinics will not be participating in the trial in any other way and once the patients have been referred, the trial staff at the Centre for Infection at St George's, University of London will take over all trial related procedures including inclusion of trial participants.

8.5 Inclusion criteria

The patient:

1. Has signed an informed consent
2. Is willing and likely to comply with the trial procedures
3. Has been diagnosed with active TB and has been in treatment ≤ 60 days at the time of inclusion
4. a) has 1 documented positive sputum smear microscopy result or

   b) has positive culture or

   c) has a positive PCR result for tuberculosis or

   d) has a compatible clinical picture of TB with the intention to treat
5. Has a positive T-spot assay or a QuantiFERON®-TB Gold In Tube test
6. Is prepared to grant authorized persons access to their medical records

8.6 Exclusion criteria

The patient:

1. Has been in treatment with a product which is likely to modify the immune response within 3 months prior to the day of inclusion (e.g., immunoglobulin, systemic corticosteroids, methotrexate, azathioprine, cyclosporine or blood products)
2. Has been vaccinated with a live vaccine within 6 weeks prior to the day of inclusion (e.g. BCG, MMR, yellow fever, oral typhoid vaccines)
3. Has a known congenital or acquired immune deficiency

4. Has a disease affecting the lymphoid organs (e.g., Hodgkin's disease, lymphoma, leukaemia, sarcoidosis

5. Is infected with HIV

6. Has severe scarring, burn, rash, eczema, psoriasis, or any other skin disease at or near the injection sites

7. Has a condition where blood drawings pose more than minimal risk for the patient, such as haemophilia, other coagulation disorders, or significantly impaired venous access

8. Is actively participating in another clinical trial

9. Is pregnant according to urine pregnancy test at inclusion

10. Has a condition which in the opinion of the investigator is not suitable for participation in the study

8.6.1 Predetermined reasons for discontinuation

Trial patients are free to withdraw from the trial whenever they desire without giving a reason for this.

In this trial there will be no pre-defined medical events/conditions, which could lead to the withdrawal of a patient, as the treatments (i.e. test products) investigated in the trial, are administered simultaneously and only once at Visit 2 (Day 0). At the following visits (Visit 3, day 1 to Visit 7, day 28), only follow-up procedures in relation to safety and the immune response assessments are performed, and the procedures of these visits will not expose a trial patient to any further risk.

A co-investigator or the principal investigator can at any time consult the Data Safety Monitoring Board for discussion of safety issues that might arise during the trial, or for advice about actions to be taken concerning a particular individual or the trial as a whole. Such meetings will normally be arranged as teleconferences. For more details see section 10.2.

The trial can be terminated at any time if the sponsor or the principal investigator concludes that the trial poses an unacceptable threat to the patients.

If, for any reason, a patient wishes to discontinue her/his participation in the trial, or if, according to principal investigator's judgement, she/he must be withdrawn from the trial, the date and reason (if possible) for withdrawal must be recorded in the CRF.

8.6.2 Permanent contraindications

Pregnant and/or breastfeeding female patients will be excluded from participation in the trial. Other permanent contraindications are listed as exclusion criteria in section 8.6.

8.6.3 Temporary contraindications

It will be the responsibility of the investigator to judge from a medical evaluation if a patient's condition on the day of the injections should lead to the postponing of the visit.

FEVER (≥ 38.3 ºC) on the day of the injections must lead to the postponing of the injections.

9 Investigational Products

The investigational product C-Tb, is manufactured at SSI in Denmark according to Good Manufacturing Practice (GMP) standards.

The w:w ratio of rdESAT-6 and rCFP-10 is 1:1 in all test solutions. For example a dose of 0.01 µg C-Tb refers to a test solution consisting of 0.005 µg rdESAT-6 and 0.005 µg rCFP-10 per 0.1 mL.

9.1 Composition

Investigational product: C-Tb

rdESAT-6..0.05/0.5 µg
rCFP-10...0.05/0.5 µg
Disodium hydrogen phosphate dihydrate.................................1.4 mg
Potassium dihydrogen phosphate..0.2 mg
Potassium chloride..0.2 mg
Sodium chloride...8.0 mg
Polysorbate 20..0.1 µL
Phenol...0.0/0.5 %
Water for injection...up to 1 mL

9.2 Treatments administered

The C-Tb reagent is formulated as a solution for injection in the following strengths:

- 0.01 µg C-Tb / 0.1 mL ± phenol
- 0.1 µg C-Tb / 0.1 mL ± phenol

C-Tb is dissolved in phosphate buffered saline containing 0.01% Polysorbate 20 and with a pH adjusted between 6.9 and 7.9. It appears as a clear, colourless solution.

The test solution is filled into 3 mL glass vials with perforable rubber stoppers and aluminium caps with white and red plastic 'flip-off' tops indicating the concentrations 0.01 and 0.1µg C-Tb, respectively. Each vial is intended for administration of 0.1 mL to one patient.

Each patient will in each arm receive one injection containing the same dose of C-Tb (0.01 µg/0.1 mL or 0.1 µg/0.1 mL) but either unpreserved or preserved with 0.5 % phenol according to a randomisation code.

9.2.1 Doses and administration

The C-Tb ± phenol skin test is administered by the Mantoux technique by staff with training and experience in the Mantoux testing technique.

The subject number given to a patient eligible for inclusion must match the subject number on the inner and outer labels of the investigational products to be injected.

It will remain unknown for the patient, the staff at the investigational site and the staff at the sponsor's site (SSI) in which arm the C-Tb +phenol skin test agent and the C-Tb -phenol skin test agent is injected, until the code will be revealed at the time of reaching 'clean file' for the whole trial.

Administration of the C-Tb ±phenol skin test agents:

- The test products should preferably be taken out of the refrigerator half an hour before each injection is performed.
- Before the administration the "Use before" date must be checked.
- The tests may only be administered if they appear as clear and colourless solutions.
- Disinfection of the injection sites before the injections is not necessary. If the injection sites are disinfected anyway, they must be completely dry before injection of the test products.
- The two vials in the patient package are clearly marked with the arm they should be applied to. For standardisation reasons the vial for RIGHT arm should always be used first.
- Use a sterile 1.0 mL disposable syringe fitted with a short-bevelled needle 26 gauge for the administration of 0.1 mL of the test solutions by the Mantoux technique.
- Stretch the skin slightly and hold the needle almost parallel to the skin with the bevelled side upwards. The needle should be inserted about 2 mm into the flexor surface of the RIGHT/LEFT forearm at the junction of the upper third with the lower two-thirds. The needle should be visible through the epidermis before SLOWLY inserting 0.1 mL of the test solution intradermally. The appearance of a small papule of 8-10 mm in diameter after the injection indicates correct injection technique. The papule disappears after approximately 10 minutes.

If one or both of the injections fail, i.e. do not result in a papule (as described above), this must be recorded in the CRF together with the reason. A failed injection must under no circumstances be

repeated in the same patient. In case one of the injections fails in a given patient, he/she should (if possible) for safety concerns stay in the trial for the follow-up visits. The patient should in this case be replaced by a new patient who will be given the next screening number available.

The syringe and the needle must be discarded as a single unit after injection in a labelled, puncture-proof container. The vial must be kept together with the cardboard box until drug accountability documents have been completed and approved by the monitor. This will normally take place at the termination monitoring visit.

9.2.2 Packaging and labelling

The packaging and labelling is performed at SSI according to Good Manufacturing Practice (GMP) standards. The C-Tb tests without phenol and the C-Tb tests with phenol cannot visually be distinguished from each other and the solutions look identical when drawn up in syringes as part of the test administration procedure.

Both vials contain the same concentration of C-Tb. One of them is preserved with phenol, the other is unpreserved. The label states in which arm the product should be injected.

9.2.3 Storage information

The stability of C-Tb is followed during the clinical trial and is expected to be minimum 12 months if stored in a refrigerator at + 2ºC to + 8ºC.

The vials should be kept in the cardboard boxes until administered. The “Use before” date is printed on the “outer” labels on the cardboard boxes. The test products are stored in a fridge at the Centre for Infection, St George’s, University of London. The refrigerator where the test products are stored is equipped with a temperature surveillance system and an alarm system. Staff at the Centre for Infection, St. George’s University of London, is responsible for the monitoring of storage conditions. Principal investigator Professor David Lewis is responsible for the dispensing of the trial product. The dispensed test solutions will be recorded in a dispensing log.

In case the refrigerator, where the test items are stored, breaks down, the test items should be moved to another refrigerator as soon as possible. The reason and time of when the test items are moved to another location must be registered in the temperature log of the test items.

In case of significant storage condition deviations, as judged by the principal investigator, the items subject to the deviations may not be used and the SSI clinical trial monitor should be contacted as soon as possible for advice. The SSI clinical monitor will seek information from relevant parties at SSI, in order to decide whether the test items, which were subject to the deviations, should be destructed or whether they can still be used in the trial.

9.2.4 Transport of trial vaccines

The C-Tb skin test agents will be packed at Statens Serum Institut and sent by courier. Temperature loggers will be packed with the trial products prior to shipping for monitoring of the temperature

conditions during the transport from Statens Serum Institut to the storage facilities at the SGUL pharmacy. The temperature loggers will be collected by SGUL upon arrival to the storage facilities, and sent back to SSI according to detailed instructions given by Statens Serum Institut.

9.2.5 Randomisation procedure

The study comprises 2 doses of C-Tb (0.01 and 0.1µg / 0.1 mL), each in two differerent formulations, one unpreserved and one preserved with 0.5% phenol.

Each patient receives both formulations in a split body design, the unpreserved is injected in one arm and the phenol preserved injected in the other arm.

The study is open with respect to the dose of C-Tb. But in order to allow an unbiased evaluation of the phenol preservation the phenol version is randomly allocated to the right or left arm.

The randomisation is performed by a statistician at the Biostatistics Unit at SSI. The statistician performing the randomisation will not in any way participate in the data management or the statistical analysis of data from the trial.

For each dose 36 test kits (3 blocks) with 2 vials each (one with phenol and one without phenol) are prepared. By means of a random permutation in a statistical analysis program (SAS), the subject numbers are randomly allocated to the 2 “phenol-groups” in blocks of 12 patients, 6 of them receiving the phenol formulation in the right arm and 6 receiving the phenol formulation in the left arm. The SAS program also prepares packing lists for the test kits, individual labels for the vials and the cardboard boxes, as well as emergency envelopes for each patient, making it possible, if necessary, to reveal the actual allocation of the phenol formulation without unblinding the whole trial.

Screening Numbers:
All patients screened will be allocated the lowest available screening number at visit 1. The available screening numbers are S501 to S999.

Subject Numbers:
At the investigational site, the physical randomisation procedure consists in allocation of the lowest available subject number to a new eligible patient at Visit 2, and administration of the skin tests with the corresponding number.

36 test kits, each with 1 vial of **0.01** µg/0.1 ml C-Tb with phenol + 1 vial of C-Tb without phenol will be labelled with subject numbers **101-136** and 36 test kits, each with 1 vial of **0.1** µg/0.1 ml C-Tb with phenol + 1 vial of C-Tb without phenol will be labelled with subject numbers **201-236**.

The 12 extra subject numbers are needed because an injection may be unsuccessful so that a patient will need to be replaced.

9.2.6 Blinding and unblinding

The C-Tb is a solution for injection and appears as ‘a clear and colourless liquid’. The cardboard boxes as well as the vials are labelled with the doses of C-Tb, but without any identification of the identity of the agent (C-Tb preserved with 0.5 % phenol or unpreserved C-Tb), see section 9.2.2.

The identity of the injections given to a particular patient’s right or left arm (C-Tb preserved with 0.5 % phenol or unpreserved C-Tb) can however be revealed by breaking a sealed emergency code envelope. There will be one set of emergency envelopes at the trial site kept under the responsibility of the principal investigator, and there will be one set in Department of Regulatory and Medical Affairs, SSI, kept under the responsibility of the Medically Responsible, SSI.

In the event of a medical emergency, the code may be broken by the investigator ONLY if the information given in the emergency code envelope is of relevance for the further treatment of the patient. In this case, the code must be kept STRICTLY CONFIDENTIAL, and must ONLY be revealed to investigational site staff directly involved in the medical emergency on a need to know basis.

Department of Regulatory and Medical Affairs, SSI may break the code ONLY if necessary to comply with serious adverse reaction reporting requirements of the competent authority or ethics committee (see section 11.4 and 11.5). In this case, the code must be kept STRICTLY CONFIDENTIAL and must under no circumstances be revealed to any person not directly involved in the reporting, including the principal investigator, the study director, the trial statistician or any person working with laboratory or statistical analysis of the trial data and interpretation of the trial results.

If the code is broken, the time, date, reason for breaking it, and, finally, the signature of the person breaking it, must be recorded on the emergency code envelope. The broken emergency code envelope must be kept together with the patient’s case record form if broken at the investigational site, or according to internal procedures at SSI if broken in Department of Regulatory and Medical Affairs, SSI.

The study director at SSI must be informed within 72 hours after breaking the code/envelope (without being informed about the actual code).

9.2.7 Treatment compliance

As the test products are injected by study staff during the study visits under controlled conditions, treatment compliance procedures are not relevant in the present clinical trial.

9.2.8 Drug accountability

All used and unused vials must be kept at the Centre for Infection, St George's, University of London until the SSI monitor has performed the final check of drug accountability as part of the termination monitoring visit.

It is the responsibility of the principal investigator to make sure that all vials are accounted for during the clinical trial and it is the responsibility of the SSI monitor to check this at the routine monitoring visits.

Upon completion of the termination monitoring visit, the SSI monitor may give permission that the vials are destructed at St George's, University of London or that the vials are returned to SSI for destruction. All dispensed test products will be recorded in a dispensing log.

9.2.9 Precautions and overdosing

Anaphylactic reactions are rarely seen in relation to tuberculin testing. Such reactions have not been seen in the previous phase Ia with C-Tb; however the necessary treatment for an anaphylactic reaction must be accessible.

A vasovagal reaction to the Mantoux injection can occur. The risk will be minimised by ensuring that the patient is seated on a reclining chair prior to administration of the skin tests. Should a vasovagal reaction occur the patient can be positioned lying down and the pulse and blood pressure being recorded until recovery. Should this event occur it will be documented in the CRF.

9.2.10 Concomitant medication

Concomitant medication considered necessary for the patient during the course of the trial should be recorded in the concomitant medication pages of the case record form (CRF) of the patient.

Decisions on withdrawal will be at the discretion of the investigator. See section 8.6 for medications and live vaccines that are listed under exclusion criteria.

9.3 Investigational events

Visit No.	1[1]	2	3	4	5	6	7	7x
Trial day	0-28 days	0	1	2	3	4	28	Term. Visit [2]
Information of patient	×							
Signing of Consent Form<br>- allocate Screening No.	×							
In-/exclusion criteria	×	×						
Demography	×							
Medical history	×							
Full medical examination[3]	×						×	×
Blood samples (central lab.)[4]	×						×	×
Blood sample for QuantiFERON®-TB Gold[5]	×							
Pregnancy testing (female volunteers, only)		×						
Inclusion/randomisation<br>- allocate Randomisation No.		×						
Skin testing with C-Tb (0.01µg and 0.1µg) ± phenol		×						
Pain assessment after injections[6]		×						
Hand out diaries						×		
Collect diaries							x	
Concomitant medication	×	×	×	×	×	×	×	×
Adverse events		×	×	×	×	×	×	×
Reaction measurement and digital photo of reaction			×	×	×	×	×	×
Trial completion							×	×

1) The screening visit (Visit No. 1) takes place up to 28 days before the Inclusion visit (Visit No. 2)
2) A termination visit will be performed if a patient is withdrawn prior to visit 7 (day 28)
3) General medical examination
4) Blood samples for: RBC, haemoglobin, haematocrit, differential WBC, platelets, AST, ALT, alkaline phosphatase, albumin, bilirubin, creatinine, glucose, potassium, sodium and HIV (HIV only at inclusion visit)
5) Blood sample for measuring *in-vitro* Interferon-γ response with the QuantiFERON®-TB Gold In Tube test
6) Pain assessment by the patient using a Visual Analogue Scale (VAS). Evaluated 1 hour after injections

9.3.1 Safety assessments

To assess the safety of C-Tb each patient will, when given the two injections, be under observation for 1 hour at the Department of Infectious Diseases, St George's, University of London. Immediate adverse events, if any will be recorded in the CRF. A record of the pain experienced by the patient due to the injections will be obtained and recorded in the CRF 1 hour after the injections. The pain from each injection will be assessed by the patient using the Visual Analogue Scale (VAS) [44].

The VAS scale ruler used in this trial is produced by Schlenker Enterprises LTD. On one side it is graded from “no pain” indicated by a smiling face to “worst possible pain” indicated by a sad face to visualize the meaning of the text. On the other side the ruler is graded from 0 mm to 100 mm. A slide marks the grade of pain equally on both sides. The ungraded side will be presented to the volunteer in a vertical position with “no pain” (smiling face) at the bottom, and he will be asked to move the line to a position corresponding to the pain experienced. The investigator will then read the evaluation of the pain for each arm in mm on the other side of the scale and record the mm in the CRF.

There will be 5 follow up visits following the injections of the skin tests on day 1, 2, 3, 4 and 28, respectively. All patients will be given a diary on day 4 where all adverse events (local and systemic) experienced up to the final day 28 should be recorded. All adverse events will be assessed by the investigator and if serious reported, as specified in Section 11.3. A detailed description of any local or systemic adverse events will be made in the CRF.

In case of local adverse events, additional images, if applicable, will be made of the injection site(s) at the time of occurrence. The course of any adverse events will be documented by sequential descriptions and, if applicable, by making digital images until the events have completely disappeared. Induration ≥ 50 mm and erythema ≥ 80 mm will be regarded as local adverse reactions.

This trial is the first trial where C-Tb will be investigated in patients diagnosed with TB. Due to the main diagnosis of TB many patients are expected to have symptoms that can be difficult to distinguish from systemic adverse events due to the investigational product, if any. However, all systemic adverse events regarded as related or non related to the C-Tb, will be reported in the CRFs.

9.3.2 Immune response assessments

The result of the classical tuberculin skin test is by consensus expressed as millimetres induration and therefore only induration will be regarded as the result of C-Tb testing. Redness will, however, also be measured in the present study, but only be regarded as an exploratory value.

Delayed-type hypersensitivity (DTH) reactions will be measured by an experienced member of the study team.

On the day of the injections, the injection sites will be marked with four dots on the flexor surface of the forearms at the junction of the upper third with the lower two-thirds with a coloured marker pen at a distance of at least 3 cm from the injection sites. This is to avoid later confusion about the precise location of the injection sites of the skin tests and to allow orientation on the digital images.

Induration is measured by palpation of the injection sites from lateral to central on all sides.

Redness is the visible red or pink discoloration of the skin around the injection sites. The border is assessed visually.

Digital images of the injection sites will be made for ALL patients at visit 3, 4, 5, 6 and 7.
Next to the arm, a label will be placed indicating the subject number, placement (LEFT/RIGHT) and visit. A ruler with millimetre indications will be placed at least 3 cm from the injection site.

C-Tb is intended used for identifying tuberculosis infection by the formation of an erythema and an induration at the injection site 48 h - 96 h after the injection (positive reaction). In the present study the aim is to determine the dose of C-Tb, which will induce a distinct skin response in TB patients. However a too strong response with an induration ≥ 50 mm and/or an erythema ≥ 80 mm will be regarded as local adverse reactions.

The preservation of C-Tb with phenol is not expected to influence the size of the skin reaction (section 6).

9.3.3 Collection and handling of blood samples

Blood samples collected by veno puncture will be drawn by experienced medical personnel according to standard procedures.

HIV test:

5 mL of clotted blood will be collected at the screening visit.

Blood samples for laboratory safety tests:

4 mL blood in EDTA tubes (PURPLE TOP) for blood count and 3.5 mL blood in coagulation tubes (YELLOW TOP) for chemistry will be collected at each sampling occasion for measuring RBC, haemoglobin, haematocrit, differential WBC, platelets, AST, ALT, alkaline phosphatase, albumin, bilirubin, creatinine, glucose, potassium and sodium

Blood samples QuantiFERON®-TB Gold In tube test:

Standard test tubes provided by the manufacturer are used, handled, stored, and analysed according to the manufacturer's instructions. Blood samples are processed on the day they are taken. 2 mL blood is drawn into the QuantiFERON®-TB Gold tubes provided by the manufacturer for measuring in-vitro Interferon-γ response.

T- SPOT assay (if relevant):

8 mL blood is drawn for measuring the in-vitro Interferon-γ response.

The accredited laboratory TDL (The Doctor's Laboratory) will be used in the trial for all analysis performed (e.g. safety tests, QuantiFERON). TDL will collect all samples and send the results to the principal investigator by email and post. TDL will use a study number and DOB, as identifiers to secure the confidentiality of the patients.

Laboratory parameters outside of normal range judged by investigator to be non-clinically significant should be marked in the print outs from TDL with "NCS" together with the date and finally initialized by investigator.

9.3.4 Time schedule

The documents to the Ethics Committee (EC) and to the Competent Authority (MHRA) are planned to be submitted in October 2009.

The first patient's first visit (FPFV) is planned to be in Q1 2010.

The last patient's last visit (LPLV) is planned to be Q2 2010.

10 Ethical Aspects

Tuberculosis continues to be a major cause of morbidity and mortality throughout the World, being one of the most important fatal infections of human beings. WHO estimates that 9.3 million new cases of tuberculosis occurred in 2007 with an estimated 1.3 million deaths in HIV negative people and an additional 456 000 deaths among HIV-positive people. [1].

To control the disease, fast and accurate diagnosis is very important. The new diagnostic tool to be investigated in this phase I clinical trial is a traditional tuberculin like skin test but in contrary to the classical tuberculin test it contains two new antigens, rdESAT-6 and rCFP-10, as the active ingredients in C-Tb. This trial is the first C-Tb trial with patients diagnosed with TB. C-Tb has previously been tested in healthy adult volunteers.

Participation in the trial is voluntary, and the patient may choose to leave the trial at any time without any specific reason.

Before inclusion in the trial, all patients will be informed (verbally and in writing, see Appendix 2) that there is a risk of unexpected adverse reactions, and that this risk has to be taken into account when considering participation. Detailed information about all trial procedures and the inconveniences they might pose on the patient will be explained as well. The patient will be informed that he or she is welcome to bring a third party to receive the information. Informed consent will be obtained from all patients (se Appendix 3).

The trial has been designed as a dose-adjusting clinical trial to minimise the risks the patients are exposed to. Furthermore, there will be at least one hour between the injections of C-TB to different patients. All patients will be observed for one hour after the injections by experienced study staff.

A Data Safety Monitoring Board will follow the progress of the clinical trial closely. The data safety monitoring board will be asked for advice when progressing from block 1 to 2 and again from block 2 to block 3, if relevant.

As the trial is a phase I trial, the patients should not expect any benefits from participation, except, for the benefit of the intensive general health status investigations that will be performed during the trial.

The total amount of blood needed from each patient will be approximately 30 mL. Blood samples will be coded before they are sent for laboratory analyses, and only the principal investigator and the study staff will have access to information that may link laboratory results with personal identification.

Based on the non-clinical pharmacology and toxicity studies, two phase I clinical trials with rdESAT-6 (alone) and one phase I trial with rdESAT-6 mixed with rCFP-10 (C-Tb), it is expected that the administration of C-Tb by the Mantoux injection technique in doses between 0.01 µg and 0.1 µg only will expose the patients to minimal risks, such as local reversible adverse reactions at the injection sites. C-Tb is not likely to give rise to systemic adverse reactions.

In conclusion, it is anticipated that there is a reasonable balance between the risks and the inconveniences to which the patients will be exposed and the benefits they might obtain as a result of the increased medical attention which is part of the clinical trial, i.e. medical examinations and laboratory investigations.

10.1 Payment of volunteers

In recognition of the time involved in participating in the study, patients will be compensated with 60 £ for each visit to the hospital, and 40 £ for each home visit which equals a total of 340 £ for the completion of all scheduled visits.

10.2 Data Safety Monitoring Board

The clinical trial is expected to expose the trial patients to minimal risks only. The principal investigator takes the responsibility for the safety of the patients in the daily clinical practice. A Data Safety Monitoring Board will follow the progress of the trial and be available for clinical advice. In addition, the Data Safety Monitoring Board will be asked for advice in the assessment of safety and the immune response data when proceeding from block 1 to block 2 and again in proceeding from block 2 to block 3, if relevant (see section 8.1 for details on the trial design).

The Data Safety Monitoring Board consists of:

Dr. (to be appointed),
Dr. (to be appointed),
Dr. (to be appointed),
Principal investigator Professor David JM Lewis (St George),
Responsible Medical Advisor Trine R. Nielsen (SSI)
Study director Pernille Nyholm Tingskov (SSI).

In a brief written statement the principal investigator will before each dose change give a review of all safety and immune response information and arrive at a recommendation as to which scenario to proceed to in block 2 and again to block 3, if relevant. This brief written statement will be provided by e-mail to the investigator in the trial, to the Data Safety Monitoring Board members and the study director as soon as possible.

The study director will be responsible for informing Trine R. Nielsen (medically responsible, SSI), Henrik Aggerbeck (project manager, SSI), Ingrid Kromann (sponsor's representative, SSI) and Jens Henrik Badsberg (statistically responsible, SSI).

Before proceeding to the next block, the principal investigator must ensure that all members of the DSMB have received copies of the assessment and have replied with their recommendations. The final decision of which scenario to follow will be confirmed by a written statement, which will be dated and signed by the Principal Investigator, Professor David JM Lewis, and by the sponsors representative, Director of Vaccine Development, SSI Ingrid Kromann.

The original statements will be filed in the trial master file at SSI and a copy in the investigator's file at Centre for Infection, St George's, University of London.

11 Adverse Events

This section reviews the procedures for recording and reporting of adverse events in the trial. Relevant definitions and terms are listed. Furthermore, the procedures for immediate reporting of serious adverse events to SSI and for expedited reporting to the competent authorities and to the local ethics committee are described

11.1 Definitions and terms

All definitions in the following are in accordance with the ICH E2A guideline [27].

Adverse Event (AE)
Any untoward medical occurrence in a patient or a volunteer participating in a clinical investigation and receiving a pharmaceutical product, which does not necessarily have a causal relationship with this product.

Adverse Reaction/Adverse Drug Reaction/Suspected Adverse Reaction (AR/ADR/SAR)
Any untoward and unintended response to an investigational product related to any dose

administered. The terms ‘Adverse Reaction’, ‘Adverse Drug Reaction and ‘Suspected Adverse Reaction’ are the same thing (in practice), and imply that there is a suspected relationship between the event and the trial product. In practice this means that there is evidence or arguments that suggest a causal relationship, i.e. a relationship cannot be ruled out.
For further details, see ICH Topic 2A and EU directive 2001/20/EC.

Seriousness criteria
A serious adverse event or reaction is any untoward medical occurrence that at any dose:

- results in death
- is life-threatening
- requires in-patient hospitalisation or prolongation of hospitalisation
- results in persistent or significant disability/incapacity
- is an important medical condition

NOTE: The term ‘life-threatening’ in the above definition refers to an event during which the patient was at risk of death at the time of the event, it does not refer to an event which hypothetically could have caused death, had it been more severe.

If a SAR (suspected adverse reaction) is serious it is a ‘serious suspected adverse reaction’ (SSAR).

For further details, see ICH Topic 2A and EU directive 2001/20/EC.

Unexpected adverse reaction
An adverse drug reaction, the nature or severity of which is **not** consistent with the applicable product information (e.g., the Investigator’s Brochure).

If a SSAR (serious suspected adverse reaction) is unexpected it is a ‘Suspected Unexpected Serious Adverse Reaction’ (SUSAR).
For further details, see ICH Topic 2A and EU directive 2001/20/EC.

11.2 Standard reporting of adverse events

The investigator is responsible for the recording of all reported adverse events in the Adverse Event Forms of the CRFs during the visits. The investigator must use the following terms:

The **causal relationship** between an adverse event and the trial tests is assessed using the following terms:

- Not related
- Possible

- Probable
- Certain

The **intensity** of an adverse event is assessed using the following terms:

- Mild (i.e. easily tolerated)
- Moderate (i.e. sufficient to interfere with daily activities)
- Severe (i.e. sufficient to prevent normal activity)

Furthermore, for adverse events that are included in the FDA Guidance for Industry, September 2007 [35], the intensity will as far as possible be rated according to the more specific rating system suggested in this guidance document.

If an adverse event is assessed as Potentially life threatening= grade 4, according to [35], this implies that the adverse event is serious and that expedited reporting to SSI by use of the CIOMS form is required, as described in section 11.3 of this protocol.

The **outcome** of an adverse event is assessed using the following terms:

- Fatal
- Not yet recovered
- Recovered with sequelae
- Recovered without sequelae
- Unknown

NOTE: If an adverse event is still ongoing at the last visit, it must be followed by the investigator until it has resolved or stabilised.

The **seriousness** of an adverse event is assessed by answering the following questions:

- Did the event result in death?
- Has the event been or is the event life-threatening?
- Has the event required inpatient hospitalisation or prolonging of hospitalisation?
- Has the event resulted in significant or persistent disability or incapacity?
- Is the event medically important

To ensure no confusion or misunderstanding of the difference between the terms "serious" and "severe," which are not synonymous, the following note of clarification is provided:

The term "severe" is often used to describe the intensity (severity) of a specific event (as in mild, moderate, or severe myocardial infarction); the event itself, however, may be of relatively minor medical significance (such as severe headache). This is not the same as "serious," which is based on patient/event outcome or action criteria usually associated with events that pose a threat to a patient's life or functioning. Seriousness (not severity) serves as a guide for defining regulatory reporting obligations

11.3 Investigator's immediate reporting of serious adverse events to SSI

If a serious adverse event/reaction (see definitions in section 11.1) occurs, the investigator is responsible for completing a CIOMS from (see template in Appendix 5), in addition to the adverse event form in the CRF [42].

The completed CIOMS form must be sent to the Department of Regulatory & Medical Affairs at SSI as soon as possible and at the latest 72 hours after his/her first knowledge of the serious adverse reaction/event.

The completed CIOMS from must be sent ***by fax*** to the following SSI address:

Statens Serum Institut
Department of Regulatory & Medical Affairs
Fax: +45 32 68 39 73
Att.: Medical Affairs

The initial report should be followed by follow-up reports (using the same form) if additional important information becomes available.

11.4 SSI's expedited reporting of SUSARs to CA and EC

The time from SSI's first knowledge of a suspected unexpected serious adverse reaction, SUSAR (see definitions in section 11.1 and 11.4) until expedited reporting to CA/EC is as follows [40, 43]:

- For SUSARs resulting in death or life-threatening events, notification no later than 7 calendar days after SSI's first knowledge of the event and reporting after an additional 8 calendar days

- For other SUSARs reporting no later than 15 calendar days after SSI's first knowledge of the event (notification prior to this is not required)

The Department of Regulatory & Medical Affairs, SSI will be responsible for reporting by email, mail or fax of **unblinded** SUSAR reports according to the above timelines to the competent authority (MHRA).

Reports scanned as PDF documents can be e-mailed to pharmacovigilance@mhra.gsi.gov.uk, clearly stating that the attachment is a ‘UK clinical trial SUSAR’.

Paper UK clinical trial SUSAR reports can be sent to MHRA’s centralised scanning facility:

Data Capture Solutions Ltd
345 Edinburgh Avenue
Slough
Berkshire UK
SL1 4TU
Great Britain

UK SUSAR reports can still be reported by fax to the MHRA using the following number(+ 44 020 7084 2443). This facility is slowly being phased out, however the date of its discontinuation is still unknown.

The Department of Regulatory and Medical Affairs, SSI will in addition be responsible for the reporting by ordinary mail of **unblinded** SUSAR reports according to the above timelines to the ethics committee (EC):

Wandsworth Ethics Committee
South London REC Office
St. George’s University of London
Room 1.14, 1st floor, Jenner Wing
Tooting, London
SW17 0RE
Great Britain

A NRES Safety Report Form enclosing the CIOMS forms should be used when reorting SUSARs to the EC [43].

Procedures for unblinding are described in section 9.2.6.

The Department of Regulatory and Medical Affairs, SSI will be responsible for distributing **blinded** copies of SUSAR reports according to the above timelines to the following parties:

- the principal investigator
- site responsible investigator(s)
- the study director at SSI

The **blinded** SUSAR reports will be filed in the trial master file by SSI and in the investigator’s file by the investigator/monitor.

11.5 Annual reporting of suspected serious adverse reactions to CA and EC

SSI is responsible for submitting annual safety update reports on **unblinded** SUSARs and SSARs to the ethics committees and the competent authority (MHRA) as electronic documents on disc to the following:

Wandsworth Ethics Committee
South London REC Office
St. George's University of London
Room 1.14, 1st floor, Jenner Wing
Tooting, London
SW17 0RE
Great Britain

Information Processing Unit, Area 6
Medicines & Healthcare products Regulatory Agency
Market Towers
1 Nine Elms Lane
Vauxhall
London SW85NQ
Great Britain

Annual Safety Reports should be prepared at yearly intervals from the date of the CTA approval and be submitted within 60 days. If the clinical trial is ended before the first yearly Annual Safety Report should have been prepared, a safety report shall be prepared within 90 days of the end of the trial, together with the end of trial notification.

The Department of Regulatory and Medical Affairs, SSI will be responsible for distributing blinded copies of these annual safety reports to the principal investigator, site responsible investigator(s) and the study director at SSI.

The blinded SUSAR reports will be filed in the trial master file by SSI and in the investigator's file by the investigator/monitor.

12 Data Management and Statistical Analysis

12.1 General considerations

The Biostatistics Unit at SSI will be responsible for the data management and the statistical analysis of the trial data.

12.2 Data management

Clinical data is collected in the CRFs. The subject diaries are considered supportive for the investigators when filling in the CRFs, i.e. only relevant information from the diaries will be transferred

to the CRFs by the investigator. The diaries will not be sent to the sponsor's site but will remain filed at the investigator's site and filed with the investigator's copy of the CRFs.

SSI will receive laboratory data as signed printouts from the investigator. The printouts are identified by subject number and date of blood sampling.

Data from the CRFs and from laboratory records will be entered into SAS data sets and checked for consistency and plausibility by custom-made SAS programs. All ambiguous or implausible data items will be resolved by data queries to the investigator. When these findings have been resolved the SAS data sets will be copied to CDs, an audit trail established for the SAS data sets, and printouts of the datasets are proofread against the CRFs and laboratory printouts for the SAS data sets.

12.3 Clean file procedures

After correction of any errors found in the proofreading process, the SAS data sets reach clean file status. The SAS data sets will then be copied to CDs. Two identical sets of CDs will be made. One CD will be archived in the trial master file. The other CD will be archived at the Biostatistics unit.

The statistical analysis reported in the integrated clinical trial report will be based on the clean file data.

12.4 Analysis populations

All subjects who have received at least one injection with a test substance will be included in the safety analysis. All subjects who have received injections with both test substances by the Mantoux technique correctly (that is, none of the two injections fails - as described in section 9.2.1) will be included in the comparison between the preserved and unpreserved formulation - with all available data.

12.5 Statistical methods

1. Dose adjustment: After each block (group of 12 patients) dose adjustment, if any, is decided from an overall clinical assessment of the safety parameters and immune responses in the previous patients (no statistical analysis is involved).

2. Induration and erythema: After the study is finished and the blinding of unpreserved/ preserved versions at C-Tb is broken the 0.01 µg and 0.1 µg are compared with two-sample Wilcoxon tests per reading time (24, 48, 72, 96 hours) of the average of the left and right arm responses. The results will be illustrated by histograms. The preserved and the unpreserved versions are compared by signed rank sum tests per dose and type of reading. The results will be illustrated by scatter plots.

3. The optimal reading time will be determined as the earliest time where maximum induration is achieved (inspection of profile plots per dose and phenol/no phenol).

4. Pain on injection: The VAS-scores of preserved and the unpreserved versions are compared by Wilcoxon signed rank sum test per dose. The results will be illustrated by scatter plots.

5. Laboratory values: The pre-trial blood values will be compared with the day 28 values by scatter plots per dose.

6. Adverse events: All adverse events will be listed.

12.6 Sample size determinations

The sample size of 12 (24) subjects in each concentration of C-Tb is mainly based on practical considerations. However, a group of 12 (24) volunteers gives 72 % (92 %) chance of discovering an unacceptable adverse reaction appearing at a true frequency of 10 %. By design, 24 subjects will be included in the final dose group.

12.7 Interim analysis

No interim statistical analyses are planed.

13 Good Clinical Practice considerations

The ICH guidelines, ICH Topic E 6 [26] and ICH Topic E 2A [27], the EC directives 2001/20/EC [28] and 2005/28/EC [29] of the European Parliament (including underlying guidance) were followed when writing this protocol.

13.1 Declaration of Helsinki

This clinical trial will be conducted in accordance with the principles of the Declaration of Helsinki (see Appendix 1).

13.2 Subject information and informed consent

TB outpatient clinics in the South West area of London (e.g. Mayday clinic in Croydon and the TB clinics at Kingston, St. Helen and St. George’s) will briefly inform potential volunteers of the TESEC-02 trial. If patients give their consent they will be referred to the trial. Study staff at the Centre for Infection at St George's, University of London will hereafter take over all trial related procedures.

Patients are informed about the trial both verbally and in writing. The information is provided by trained staff in an objective way, describing both advantages and disadvantages of participation in the trial. The patient will be informed that he or she is welcome to bring a third party to receive the information and will be given reasonable time (min 24 hours) to consider whether he/she wishes to participate.

The written information and the informed consent form [37] are approved by the relevant ethics committee (EC) and competent authority (CA) before use. Documents are enclosed in Appendices 2 and 3.

13.3 Independent ethics committee submission and approval

An application file will be submitted for approval to both Ethics Committee and the R&D (Joint Research) Office for host site approval [38, 39, 41], and the inclusion of patients will only start AFTER the trial has been approved by both instances:

Wandsworth Ethics Committee
South London REC Office
St. George's University of London
Room 1.14, 1st floor, Jenner Wing
Tooting, London
SW17 0RE
Great Britain

Joint Research Office
St. George's Healthcare
Ground Floor, Hunter Wing
Tooting, London
SW17 0RE
Great Britain

13.4 Competent authority submission and approval

An application file will be submitted for approval to the regulatory authorities in England [40, 41]:

Medicines & Healthcare products Regulatory Agency, MHRA
Information Processing Unit, Area 6
Market Towers
1 Nine Elms Lane
London, SW8 5NQ
Great Britain

Only after approval by both the above mentioned Ethics Committees and the MHRA the clinical trial will be initiated.

13.5 Subject data protection

The principal investigator is responsible for keeping a list (a screening log) of all trial patients. All patients who are subject to at least one trial intervention such as for example the extended medical examination or parts of it at the screening visit should be listed in the screening log. For screening failures, the reason for exclusion should be given in the screening log.

The screening log must identify the patients by: screening number, subject number (i.e. only given to patients who are included/have received the C-Tb skin tests), full name and last address known, sex, and date of birth. At the end of the clinical trial the screening log must be signed off by the principal investigator for correctness. This is done as part of the termination monitoring visit.

In the CRF, only screening number, subject number and date of birth must be recorded. It must always be possible to trace a CRF to the screening log.

The patients must be informed about and agree to the following:

- After the end of the clinical trial (i.e., after the date of the last visit of the last subject), the principal investigator will store the screening log for at least 15 years.

- After the end of the clinical trial, the screening log must be accessible by EC or CA for at least 15 years if needed in connection with an inspection..

13.6 Investigator's responsibilities

By signing this protocol, the principal investigator and the site responsible investigator(s) take the overall responsibility for the conduct of the clinical trial in accordance with the protocol, good clinical practice (GCP) [26, 27, 28, 29] and any relevant national regulations, for the complete and accurate recording of all data, including all reported adverse events in the CRFs, and for immediate reporting of serious adverse events to SSI, according to the procedures described in section 11 of this protocol.

The delegation of responsibilities to subinvestigators/nurses/important staff members must be specified in writing in the log of staff, or a similar trial document according to instructions, see the following section.

13.7 Curricula vitae and log(s) of staff

Investigational site(s):
Before initiation of the trial, current dated and signed curricula vitae in English for all investigators and staff members with significant responsibilities in the trial must be collected.

The log of staff of ALL staff members at the investigational site(s) must be completed with responsibilities (incl. start date and end date of responsibilities), signature and initials (written by her/him self) by the staff member.

The monitor must go through the log of staff with the responsible investigator, for completeness and correctness at the trial initiation visit. The responsible investigator will document this by signing off the log of staff with signature, date and initials.

During the active phase of the trial, the monitor must regularly check that the log of staff remains complete and correct at all times and resolve any outstanding issues.

Sponsor's site(s):
At the sponsor's site current, dated and signed curricula vitae in English for the study director and monitor(s) etc. involved in the trial must be available. Furthermore, ALL staff members involved in the trial must be listed in a log of staff.

For SSI staff members who may make recordings in the CRFs (according to SSI SOPs) or other trial documents, initials must be recorded in the log off staff.

13.8 Indemnity statement

Prior to the inclusion of the first patient in the clinical trial, SSI will present a signed Indemnity Statement to all investigators participating in the clinical trial (see Appendix 4).

13.9 Training

The principal investigator is overall responsible for, that all staff members at the trial site are adequately qualified before the initiation of the trial.

The study director will be responsible for performing training sessions covering general GCP issues as: safety procedures, reporting of Suspected Unexpected Serious Adverse Reactions (SUSARs), information of patients, recording in CRFs, handling of test products, randomisation procedures, blinding and unblinding procedures etc. If training is needed in relation to the Mantoux testing technique, tuberculosis, etc., the principal investigator will perform these sessions. All performed sessions will be documented in the CVs of the investigators and the staff members performing the Mantoux testing etc.

Previous GCP qualifications and experience must be stated in the current dated and signed CVs of the study staff. For investigators and nurses (or other staff members with significant responsibilities in the trial) who have no previous documented training in GCP, GCP certificates will be issued by study director upon satisfactory participation in the above listed GCP training session(s).

13.10 Monitoring

The clinical trial monitor from SSI will make regular visits to the investigational site at St. George’s University. Together with the study staff at the site, the monitor will, for example, check the following:

- That the protocol is being followed
- That facilities and staffing remain acceptable
- That the CRFs are being correctly filled in
- That the CRFs are in accordance with source data
- That the clinical supplies (especially the test products) can be accounted for
- That the test products have been transported according to requirements and are stored properly at the investigational site
- That the Investigator’s File is being kept in proper order

All visits to the investigational site are documented. Any query is discussed and resolved with the investigator or relevant study staff. The monitor will work according to a monitoring plan approved by the study director and will follow SSI's GCP SOPs for GCP-monitoring.

13.11 Audit and inspection

The investigator must give access to personnel from SSI for the conduct of audits at the investigational site. Auditors and inspectors from CA, EC or other relevant authorities must at all times be allowed access to conduct inspections as well and must be given access to all trial related documents, including the Investigator's File and the patients' personal medical records, if applicable.

13.12 Definition and archiving of source data

Source data is defined as all information in original records and certified copies of original records necessary for the reconstruction and evaluation of the clinical trial. A document, which identifies the source data in this clinical trial, will be prepared and signed by responsible investigator before initiation of the trial. In the following examples of source data are listed:

- EC approval documents
- Approval from the MHRA
- Signed informed consent forms
- The screening log
- Investigator and nurse notes (i.e. for data either not recorded in the CRF or for data not recorded directly in the CRF)
- Medical records/journals
- Print outs of laboratory results
- The CRF (for data recorded directly in the CRF)
- Patient diaries
- Test products inventory and accountability logs
- Test products storage condition log(s)

13.13 Definition and archiving of essential documents

It is the responsibility of the principal investigator and SSI to maintain the essential documents as described in the ICH guidelines for at least 15 years after the termination of the trial. In the present clinical trial termination is defined as the last patient's last visit.

The completeness of the TMF and the IF are verified by the monitor at trial initiation, during the active phase of the trial and, finally, at the end of the trial as part of the trial termination. At the trial termination visit at the investigational site(s), the monitor goes through the archiving requirements with the responsible investigator and will assure that the archiving requirements are met.

14 Agreement and financial settlement

The agreement between the Investigational institution (SGUL) and SSI must be signed prior to inclusion of the first patient in the clinical trial. The agreement must clearly state the rights and obligations of the parties concerned and include a detailed financial settlement.

Furthermore, a written agreement should be made between the principal investigator and the accredited laboratory TDL (The Doctor's Laboratory), regarding the laboratory analysis in the trial.

15 Insurance

SSI is the sponsor and manufacturer of the test products to be administered in this clinical trial. SSI carries a product liability insurance under a worldwide liability programme written by the New Hampshire Insurance Company, through insurance brokers Marsh A/S, Teknikerbyen 25, DK-2830 Virum, Denmark, as part of the worldwide Marsh insurance broker group. The policy covers claims arising from injury/injuries caused by trial medication used in clinical trials sponsored by the company, if the trial medication has been used in accordance with the instructions given in the protocol. The insurance certificate is enclosed in Appendix 6.

16 Confidentiality and disclosure

All CRFs, information and results generated by SSI, as well as information on product development, patented or not, including patent applications and manufacturing processes not previously published, are considered confidential and shall remain the sole property of SSI.

An integrated statistical and clinical study report (CSR) will be prepared by SSI in co-operation with the principal investigator. A summary of the CSR will be submitted to the CA no later than one year after the end of the trial (the end is defined as the last patient's last visit).

No data from the clinical trial may be published, presented or communicated, except to competent authorities or ethics committees, prior to the release of the internal clinical trial report, unless approved by SSI in writing. All investigators agree not to discuss externally or publish any result from the trial without the possibility of SSI to give comments.

In the event of the publication of the trial results in a scientific journal the names of the authors and their order of appearance will be as specified in the agreement between the investigational institution/site (SGUL) and SSI.

17 Changes to the protocol

The clinical trial procedures may be changed if the principal investigator and the study director agree to the changes. If the changes are substantial, both the Ethics Committee and Competent Authorities must approve the changes before they can be implemented. All substantial changes must be documented by protocol amendments and if necessary rewritten full protocols.

18 References

[1] World Health Organisation. Global Tuberculosis Control- epidemiology, strategy, financing. WHO Report 2009, (WHO/HTM/TB/2009.411)

[2] http://www.undp.org/mdg/

[3] TB diagnosis - a challenge to science and policy, EAGLES European Action on Global Life Sciences. November 2008

[4] Lange, C; Pai, M; Drobniewski, F and Miglior, G. B. Interferon-γ release assays for the diagnosis of active tuberculosis: sensible or silly. Eur Respir J 2009: 1250-1253

[5] Pai Madhukar, Kalantri Shriprakash, Dheda Keertan. New tools and emerging technologies for the diagnosis of tuberculosis: Part 1. Latent tuberculosis. Expert Rev. Mol. Diagn. 6(3), (2006). 2006 Future Drugs Ltd.

[6] Snider DE. Tuberculin test. Am Rev Respir Dis 1982; 125: 108-18

[7] Huebner RE, Schein MF, Bass JB. The tuberculin skin test. Clin Infect Dis 1993; 17: 968-75

[8] Andersen P, Munck ME, Pollock JM, Doherty TM. Specific immune-based diagnosis of tuberculosis. Lancet 2000; 356: 1099-104

[9] Andersen P, Andersen AB, Sorensen AL, Nagai S. Recall of long-lived immunity to Mycobacterium tuberculosis infection in mice. J Immunol 1995; 154: 3359-72

[10] A. H van Pinxteren, Laurens et al. Diagnosis of Tuberculosis Based on Two Specific Antigens ESAT-6 and CFP10.Clinical and Diagnostic Laboratory Immunology, Mar 2000, p. 155-160

[11] Pai Madhukar, Riley Lee W, Colford John M Jr. Interferon-γ assays in the immunodiagnosis of tuberculosis: a systemic review. The Lancet, Infectious Diseases Vol 4 December 2004.

[12] Ulrichs T, Munk ME, Mollenkopf H, et al. Differential T-cell responses to Mycobacterium tuberculosis ESAT-6 in tuberculosis patients and healthy donors. Eur J Immunol 1998; 28: 3949-58

[13] Ravn P, Demissie A, Eguale T, et al. Human T-cell responses to the ESAT-6 antigen from Mycobacterium tuberculosis. J Infect Dis 1999; 179: 637-45

[14] Johnson PD, Stuart RL, Grayson ML, Olden D, Clancy A, Ravn P, Andersen P, Britton WJ, Rothel JS. Tuberculin-purified protein derivative-, MPT-64-, and ESAT-6-stimulated gamma interferon responses in medical students before and after M. bovis BCG vaccination and in patients with tuberculosis. Clin Diagn Lab Immunol 1999; 6: 934-37

[15] Lein AD, von Reyn CF, Ravn P, Horsburgh CR et al.Cellular immune responses to ESAT-6 discriminate between patients with pulmonary disease due to M.avium complex and those with pulmonary disease due to M. tuberculosis.Clin Diagn Lab Immunol 1999; 6: 606-609

[16] Skjøt, R.L.V., T. Oettinger, I Rosenkrands, P. Ravn, I. Brock, S. Jacobsen, P. Andersen. Comparative evaluation of low-molecular-mass T-cell antigens from Mycobacterium tuberculosis identifies members of the ESAT-6 family as immunodominant. Infect. Immun. 2000, 68: 214-220

[17] Elhay MJ, Oettinger T, Andersen P. Delayed type hypersensitivity responses to ESAT-6 and MPT-64 from Mycobacterium tuberculosis in the guinea pig. Infect Immune 1998; 66: 3454-6.

[18] Reece S.T., Stride N., Ovendale P., Reed S.G. et al. Skin test performed with highly purified Mycobacterium tuberculosis recombinant protein triggers tuberculin schock in infected guinea pigs. Infection and Immunity, June 2005, p. 3301-3306.

[19] Internal SSI Report F1137

[20] Arend, Sandra M et al. Double blind randomized Phase 1 study comparing rdESAT-6 to tuberculin as skin test reagent in the diagnosis of tuberculosis infection. Tuberculosis (2007), doi: 10.1016/j.tube.2007.11.004.

[21] Thierry-Carstensen B, Aggerbeck H and Jensen A.M. A phase I (first in man), double blind, “within subject” randomized and controlled, dose-adjusting clinical trial on the safety and the diagnostic potential of the rdESAT-6 skin test in the diagnosis of tuberculosis. Internal clinical study report date of issue 04-01-2008.

[22] Lillebæk T, Bergstedt W, Tingskov PN, Thierry-Carstensen B, Aggerbeck H, Hoff ST, Weldingh K, Andernsen P, Søborg B, Thomen VO, Andersen AB. Risk of sensitization in healthy adults following repeated administration of rdESAT-6 skin test reagent by the Mantoux injection technique. Tuberculosis 2009;89:158-62

[23] Tingskov P N, Thierry-Carstensen B, Aggerbeck, Jensen A. A phase I clinical trial to assess the risk of sensitisation in healthy adults following repeated administration of rdESAT-6 by the Mantoux injection technique. Internal clinical study report date of issue 03-04-2009.

[25] Aggerbeck H, Madsen S.M. Safety of ESAT-6. Elsevier, Tuberculosis (2006) 86; 363-373

[26] Note for guidance on good clinical practice. ICH Topic E 6. (CPMP/135/95)

[27] Note for guidance on clinical safety data management: definitions and standards for expedited reporting ICH Topic E 2 A (CPMP/ICH/377/95)

[28] Directive 2001/20/EC of the European Parliament and the council of 4 April 2001 on the approximation of the laws, regulations and administrative provision of the Member States relating

to the implementation of good clinical practice in the conduct of clinical trials on medicinal products for human use. Official Journal L 121, 01/05/2001 P.0034 – 0044.

[29] Directive 2005/28/EC of the European Parliament of 8 April 2005 laying down principles and detailed guidelines for good clinical practice as regards investigational medicinal products for human use, as well as the requirements for authorisation of the manufacturing or importation of such products.

[30] Note for guidance on pre-clinical safety evaluation of biotechnology derived pharmaceuticals. ICH Topic S 6. (CPMP/ICH/302/95)

[31] Note for guidance on non-clinical safety studies for the conduct of human clinical trials for pharmaceuticals. ICH Topic M 3 (M).CPMP/ICH/286/95, modification)

[32] Topic S 7A. Safety pharmacology studies for human pharmaceuticals (CPMP/ICH/539/00) March 2000

[33] Points to Consider on the Evaluation of Diagnostic Agents. Committee for Proprietary Medicinal Products (CPMP/EWP/1119/98)

[34] Guideline on strategies to identify and mitigate tasks for first-in-human clinical trials with investigational medicinal products. Doc. Ref. EMEA/CHMP/SWP/28367/07; London 19 July 2007

[35] Guidance for Industry: Toxicity grading scale for healthy adult and adolescent volunteers enrolled in preventive vaccine clinical trials, FDA September 2007

[36] Renshaw P. Lightbody K.L, Veverka V et al. Structure and function of the complex formed by the tuberculosis virulence factors CFP-10 and ESAT-6. The EMBO Journal, 2005, 24, 2491-2498

[37] Information sheets & Consent forms – Guidance for researchers and reviewers, Version 3.2 May 2007 (NRES)

[38] Guidance for applicants to the National Research Ethics Service, 2007 (NRES)

[39] Information sheet 5: Research Ethics, 2007, Wandsworth Teaching PCT

[40] MHRA Homepage; Clinical trial authorisations, www.mhra.gov.uk

[41] IRAS – Integrated Research Application System, www.myresearchproject.org.uk

[42] Detailed guidance on the collection, verification and presentation of adverse reaction reports arising from clinical trials on medicinal products for human use, Eudralex Vol. 10 Chapter II, rev2 2006.04

[43] NRES Homepage; Safety reports for CTIMPs, www.nres.npsa.nhs.uk

[44] Jensen Mark P et al. Interpretation of Visual Analogue Scale Ratings and Change Scores: A reanalysis of Two Clinical Trials of Postoperative Pain. The Journal of Pain, vol 4, No 7, 2003: pp 407-414

WORLD MEDICAL ASSOCIATION DECLARATION OF HELSINKI
Ethical Principles for Medical Research Involving Human Subjects

Adopted by the 18th WMA General Assembly, Helsinki, Finland, June 1964, and amended by the:
29th WMA General Assembly, Tokyo, Japan, October 1975
35th WMA General Assembly, Venice, Italy, October 1983
41st WMA General Assembly, Hong Kong, September 1989
48th WMA General Assembly, Somerset West, Republic of South Africa, October 1996
52nd WMA General Assembly, Edinburgh, Scotland, October 2000
53th WMA General Assembly, Washington 2002 (Note of Clarification on paragraph 29 added)
55th WMA General Assembly, Tokyo 2004 (Note of Clarification on Paragraph 30 added)
59th WMA General Assembly, Seoul, October 2008

A. INTRODUCTION

1. The World Medical Association (WMA) has developed the Declaration of Helsinki as a statement of ethical principles for medical research involving human subjects**,** including research on identifiable human material and data.

   The Declaration is intended to be read as a whole and each of its constituent paragraphs should not be applied without consideration of all other relevant paragraphs.

2. Although the Declaration is addressed primarily to physicians, the WMA encourages other participants in medical research involving human subjects to adopt these principles.

3. It is the duty of the physician to promote and safeguard the health of patients, including those who are involved in medical research. The physician's knowledge and conscienceare dedicated to the fulfilment of this duty.

4. The Declaration of Geneva of the WMA binds the physician with the words, “The health of my patient will be my first consideration,” and the International Code of Medical Ethics declares that, “A physician shall act in the patient's best interest when providing medical care.”

5. Medical progress is based on research that ultimately must include studies involving human subjects. Populations that are underrepresented in medical research should be provided appropriate access to participation in research.

6. In medical research involving human subjects, the well-being of the individual research subject must take precedence over all other interests.

7. The primary purpose of medical research involving human subjects is to understand the causes, development and effects of diseases and improve preventive, diagnostic and therapeutic interventions (methods, procedures and treatments). Even the best current interventions must be evaluated continually through research for their safety, effectiveness, efficiency, accessibility and quality.

8. In medical practice and in medical research, most interventions involve risks and burdens.

9. Medical research is subject to ethical standards that promote respect for all human subjects and protect their health and rights. Some research populations are particularly vulnerable and need special protection. These include those who cannot give or refuse consent for themselves and those who may be vulnerable to coercion or undue influence.

10. Physicians should consider the ethical, legal and regulatory norms and standards for research involving human subjects in their own countries as well as applicable international norms and standards. No national or international ethical, legal or regulatory requirement should reduce or eliminate any of the protections for research subjects set forth in this Declaration.

B. PRINCIPLES FOR ALL MEDICAL RESEARCH

11. It is the duty of physicians who participate in medical research to protect the life, health, dignity, integrity, right to self-determination, privacy, and confidentiality of personal information of research subjects.

12. Medical research involving human subjects must conform to generally accepted scientific principles, be based on a thorough knowledge of the scientific literature, other relevant

sources of information, and adequate laboratory and, as appropriate, animal experimentation. The welfare of animals used for research must be respected.

13. Appropriate caution must be exercised in the conduct of medical research that may harm the environment.

14. The design and performance of each research study involving human subjects must be clearly described in a research protocol. The protocol should contain a statement of the ethical considerations involved and should indicate how the principles in this Declaration have been addressed. The protocol should include information regarding funding, sponsors, institutional affiliations, other potential conflicts of interest, incentives for subjects and provisions for treating and/or compensating subjects who are harmed as a consequence of participation in the research study. The protocol should describe arrangements for post-study access by study subjects to interventions identified as beneficial in the study or access to other appropriate care or benefits.

15. The research protocol must be submitted for consideration, comment, guidance and approval to a research ethics committee before the study begins. This committee must be independent of the researcher, the sponsor and any other undue influence. It must take into consideration the laws and regulations of the country or countries in which the research is to be performed as well as applicable international norms and standards but these must not be allowed to reduce or eliminate any of the protections for research subjects set forth in this Declaration. The committee must have the right to monitor ongoing studies. The researcher must provide monitoring information to the committee, especially information about any serious adverse events. No change to the protocol may be made without consideration and approval by the committee.

16. Medical research involving human subjects must be conducted only by individuals with the appropriate scientific training and qualifications. Research on patients or healthy volunteers requires the supervision of a competent and appropriately qualified physician or other health care professional. The responsibility for the protection of research subjects must always rest with the physician or other health care professional and never the research subjects, even though they have given consent.

17. Medical research involving a disadvantaged or vulnerable population or community is only justified if the research is responsive to the health needs and priorities of this population or community and if there is a reasonable likelihood that this population or community stands to benefit from the results of the research.

18. Every medical research study involving human subjects must be preceded by careful assessment of predictable risks and burdens to the individuals and communities involved in the research in comparison with foreseeable benefits to them and to other individuals or communities affected by the condition under investigation.

19. Every clinical trial must be registered in a publicly accessible database before recruitment of the first subject.

20. Physicians may not participate in a research study involving human subjects unless they are confident that the risks involved have been adequately assessed and can be satisfactorily managed. Physicians must immediately stop a study when the risks are found to outweigh the potential benefits or when there is conclusive proof of positive and beneficial results.

21. Medical research involving human subjects may only be conducted if the importance of the objective outweighs the inherent risks and burdens to the research subjects.

22. Participation by competent individuals as subjects in medical research must be voluntary. Although it may be appropriate to consult family members or community leaders, no competent individual may be enrolled in a research study unless he or she freely agrees.

23. Every precaution must be taken to protect the privacy of research subjects and the confidentiality of their personal information and to minimize the impact of the study on their physical, mental and social integrity.

24. In medical research involving competent human subjects, each potential subject must be adequately informed of the aims, methods, sources of funding, any possible conflicts of interest, institutional affiliations of the researcher, the anticipated benefits and potential risks of the study and the discomfort

it may entail, and any other relevant aspects of the study. The potential subject must be informed of the right to refuse to participate in the study or to withdraw consent to participate at any time without reprisal. Special attention should be given to the specific information needs of individual potential subjects as well as to the methods used to deliver the information. After ensuring that the potential subject has understood the information, the physician or another appropriately qualified individual must then seek the potential subject's freely-given informed consent, preferably in writing. If the consent cannot be expressed in writing, the non-written consent must be formally documented and witnessed.

25. For medical research using identifiable human material or data, physicians must normally seek consent for the collection, analysis, storage and/or reuse. There may be situations where consent would be impossible or impractical to obtain for such research or would pose a threat to the validity of the research. In such situations the research may be done only after consideration and approval of a research ethics committee.

26. When seeking informed consent for participation in a research study the physician should be particularly cautious if the potential subject is in a dependent relationship with the physician or may consent under duress. In such situations the informed consent should be sought by an appropriately qualified individual who is completely independent of this relationship.

27. For a potential research subject who is incompetent, the physician must seek informed consent from the legally authorized representative. These individuals must not be included in a research study that has no likelihood of benefit for them unless it is intended to promote the health of the population represented by the potential subject, the research cannot instead be performed with competent persons, and the research entails only minimal risk and minimal burden.

28. When a potential research subject who is deemed incompetent is able to give assent to decisions about participation in research, the physician must seek that assent in addition to the consent of the legally authorized representative. The potential subject's dissent should be respected.

29. Research involving subjects who are physically or mentally incapable of giving consent, for example, unconscious patients, may be done only if the physical or mental condition that prevents giving informed consent is a necessary characteristic of the research population. In such circumstances the physician should seek informed consent from the legally authorized representative. If no such representative is available and if the research cannot be delayed, the study may proceed without informed consent provided that the specific reasons for involving subjects with a condition that renders them unable to give informed consent have been stated in the research protocol and the study has been approved by a research ethics committee. Consent to remain in the research should be obtained as soon as possible from the subject or a legally authorized representative.

30. Authors, editors and publishers all have ethical obligations with regard to the publication of the results of research. Authors have a duty to make publicly available the results of their research on human subjects and are accountable for the completeness and accuracy of their reports. They should adhere to accepted guidelines for ethical reporting. Negative and inconclusive as well as positive results should be published or otherwise made publicly available. Sources of funding, institutional affiliations and conflicts of interest should be declared in the publication. Reports of research not in accordance with the principles of this Declaration should not be accepted for publication.

C. ADDITIONAL PRINCIPLES FOR MEDICAL RESEARCH COMBINED WITH MEDICAL CARE

31. The physician may combine medical research with medical care only to the extent that the research is justified by its potential preventive, diagnostic or therapeutic value and if the physician has good reason to believe that participation in the research study will not adversely affect the health of the patients who serve as research subjects.

32. The benefits, risks, burdens and effectiveness of a new intervention must be tested against those of the best current proven intervention, except in the following circumstances:
    - The use of placebo, or no treatment, is acceptable in studies where no current proven intervention exists; or
    - Where for compelling and scientifically sound methodological reasons the use of

placebo is necessary to determine the efficacy or safety of an intervention and the patients who receive placebo or no treatment will not be subject to any risk of serious or irreversible harm. Extreme care must be taken to avoid abuse of this option.

33. At the conclusion of the study, patients entered into the study are entitled to be informed about the outcome of the study and to share any benefits that result from it, for example, access to interventions identified as beneficial in the study or to other appropriate care or benefits.

34. The physician must fully inform the patient which aspects of the care are related to the research. The refusal of a patient to participate in a study or the patient’s decision to withdraw from the study must never interfere with the patient-physician relationship.

35. In the treatment of a patient, where proven interventions do not exist or have been ineffective, the physician, after seeking expert advice, with informed consent from the patient or a legally authorized representative, may use an unproven intervention if in the physician's judgement it offers hope of saving life, re-establishing health or alleviating suffering. Where possible, this intervention should be made the object of research, designed to evaluate its safety and efficacy. In all cases, new information should be recorded and, where appropriate, made publicly available.

St George's
University of London
Cranmer Terrace
London SW17 0RE
Switchboard
+44 (0)20 8672 9944
www.sgul.ac.uk

STATENS
SERUM
INSTITUT

PATIENT INFORMATION SHEET

"A safety and dose finding trial of the diagnostic test named C-Tb, when given to adult patients recently diagnosed with active tuberculosis"

PART 1

1 Invitation

We would like to invite you to participate in this research study, to help us develop a new skin test for the diagnosis of tuberculosis (TB). Before you decide, you need to understand why the research is being done, and what it would involve for you. Please take time to read the following information carefully. Talk to others about the study if you wish.

Part 1 will tell you the purpose of this study and what will happen to you if you take part and part 2 will give you more detailed information about the conduct of the study.

2 What is the purpose of the study?

Tuberculosis continues to be the single most important bacterial infection worldwide, with 9 million new cases every year. Worldwide the majority of patients with tuberculosis are still diagnosed with tools used for more than half a century, such as the Tuberculin Skin Test (TST) using the Purified Protein Derivative (PPD). The tuberculin skin test has, however, the disadvantage of not being able to distinguish between patients really infected with TB, patients already treated for TB, or patients vaccinated against TB (with the BCG vaccine - Bacillus Calmette Guérin vaccine). Based on this information a new, more specific skin test has been developed by Statens Serum Institut in Denmark.

The new skin test, named C-Tb, has in a clinical study conducted in Denmark, already safely been injected to 40 healthy adults. The aim of this study is to test the C-Tb skin test in adults diagnosed with TB to determine the safety, immune response and dose level of this new diagnostic test.

3 Why have I been invited?

In this study we will include 12-36 patients between 18-65 years of age who have been diagnosed with TB and who have not received treatment for TB for more than 2 months. You have been invited to participate as we believe you meet these criteria.

St George's
University of London
Cranmer Terrace
London SW17 0RE
Switchboard
+44 (0)20 8672 9944
www.sgul.ac.uk

STATENS
SERUM
INSTITUT

4 Do I have to take part?

Participating in this study is entirely voluntary, and it is up to you to decide if you would like to take part. We will describe the study and go through this information sheet, which we will then give to you. We will then ask you to sign a consent form to show you have agreed to take part. You are free to withdraw at any time, without giving a reason. This will not affect the standard of care you receive.

5 What will happen to me if I take part?

The study will continue for approximately one year. Your participation in the study will be approximately 1 month.

You will be included in the study if you choose to sign the informed consent. Experienced clinical staff will take blood samples from you for a number of routine laboratory tests. As a defective immune system can interfere with your response to the skin tests, we would like to take a sample of your blood for an HIV test. Before doing so you will have a discussion with a trained nurse, who will also give you the result. You may of course withdraw from the study without having the HIV test. All results are confidential and will not be revealed to anyone except at your specific request.

A number of specific tests have already been taken to diagnose you with tuberculosis, however to fulfil the inclusion criteria of this study extra tuberculosis diagnostic tests (blood testing) may be necessary. You will at all times be informed of which tests may be relevant in your case

A doctor will record your medical history and examine you (this involves listening to your heart and lungs, palpating your abdomen and examining your skin).

On the day of skin testing all female volunteers will be tested for pregnancy.

At this early stage of development we do not know which dose of the new C-Tb skin test will be best for the diagnosing of tuberculosis. To find out, we need to compare two different dose levels of 0.01microgram and 0.1 microgram of C-Tb. You will only receive one dose level according to the group you are recruited to. The lowest dose will be tested first and only after all controls for safety of the participants are in order, the next dose level will be given. To try to make the two dose groups as equal as possible, each patient is put into a group by chance (randomly).

On the day of skin testing you will be given two injections of the C-Tb skin test - one in each forearm -of either 0.01 micrograms or 0.1 micrograms of C-Tb, depending on which group you are placed in. The skin tests will be injected just below the skin. The two skin tests will only differ in the presence of a preservative (0.5% phenol) to compare the immune response and safety of an added preservative. This study is 'double blind' in this regards, which means that neither you nor your doctor/nurse will know which arm is injected with the C-Tb skin test preserved with phenol and in which arm the unpreserved C-Tb is injected. If your doctor needs to find out he/she can do so. For safety reasons you will be asked to stay at the clinic for 1 hour after the skin test injections. Before leaving the clinic you will be asked if you experience any side-effects and if the injection of the skin test caused any pain to you.

St George's
University of London
Cranmer Terrace
London SW17 0RE
Switchboard
+44 (0)20 8672 9944
www.sgul.ac.uk

STATENS
SERUM
INSTITUT

The following four days, you can choose to come to St George's University of London or a nurse will visit you at your home, to examine and photograph the injection site. The photographs taken will only be of the injection site to compare the skin test reactions, if any, in each arm, and will be treated with confidentiality.

On the fourth visit the nurse will give you a diary for you to take home and document any side effects that may occur or any medication you take during the next 24 days. The 28th day (the final day) after the injections a new medical examination will be performed and new blood samples will be drawn for a number of routine safety laboratory tests.

6 Expenses and payment

You will receive a compensation of £60 for each visit to the hospital, and £40 for each home visit. The compensation will be given to you after you have completed all scheduled visits in the trial. If you withdraw from the study before you have had all visits, you will receive an amount equalling the visits you have completed.

7 What will I have to do?

This study does not require any specific lifestyle restrictions other than ensuring that you make yourself available for all study visits and comply with the instructions provided to you. You can continue your treatment for tuberculosis as your physician has prescribed.

On day four after the skin testing you will be given a diary, in which you should write down all medications you take and all experienced side effects for the next 24 days. This is very important for the quality of the research. At the final examination you will give the diary to the nurse, and the study is completed.

It is recommendable that neither you nor your partner becomes pregnant during the course of the study, as the effects of the C-Tb product on an unborn child are not yet known.

8 What is the drug that is being tested?

The two proteins ESAT-6 and CFP-10 have been identified as parts of the tuberculosis bacteria. In this new skin test we use recombinant proteins, which mean that the proteins have been changed so they can be made artificially to ensure that this skin test only diagnoses persons really infected with tuberculosis. No harmful part of the bacteria is included.

The drug is tested in two doses, 0.01 and 0.1 µg and is given as an injection just below the skin, using the same method (Mantoux test) as the tuberculin skin test (TST)

9 What are the possible disadvantages and risks of taking part?

The efficacy of the new skin test is expressed by redness and induration (swelling). Therefore a skin test reaction is expected, similar to the reactions you may have experienced by a previous tuberculin test.

St George's
University of London
Cranmer Terrace
London SW17 0RE
Switchboard
+44 (0)20 8672 9944
www.sgul.ac.uk

STATENS SERUM INSTITUT

Pregnancy

The effects of the C-Tb skin tests on an unborn child are not known as no studies have been performed on pregnant women or animals. Although extremely unlikely, there is always a theoretical possibility that the C-Tb product may harm an unborn child by some unexpected mechanism. For this reason it is routine practice to exclude pregnant women, women who plan to become pregnant or nursing mothers from these early trials until more information has been obtained. Women who could become pregnant during the study will be tested for pregnancy before the injections of the skin tests and will be recommended to use an effective contraceptive during the course of the study. Any woman who finds or suspects that she has become pregnant while participating in the study should inform the research physician immediately.

Procedures

Blood sampling can sometimes cause bruising and soreness of the arms. Very rarely a blockage of a vein or a small nerve injury can occur, which can cause numbness and pain. Normally, such problems resolve with time.

Please do not hesitate to ask the research physician if you should have any questions regarding the study procedures or possible side effects

10 What are the side effects of any treatment received when taking part?

Based on previous clinical studies we do believe that the injection of the C-Tb skin tests in doses between 0.01 µg and 0.1 µg only will expose you to minimal risks, such as e.g. undesired large redness and swelling reactions at the injection sites. However as C-Tb is a new drug, there might be unknown side effects.

11 What are the possible benefits of taking part?

There are no direct benefits of taking part in the study, however new, more effective, and more cost-efficient diagnostic tools can only be developed through research. We would therefore like to thank you for considering participation in this research study. Ultimately, the information we get from this study will help us improve a future diagnostic skin test, making it easier to detect TB in infected patients.

12 What happens when the study stops?

After the termination visit, your medical information will be used to determine the safety and the best dose for the new product, and whether it is safe to include a preservative in the test. At the end of the study a report is written and a research article on the conclusions of the study will be published. Your personal details will not be disclosed in any of these publications.

13 What if there is a problem?

Any complaint about the way you have been dealt with during the study or any possible harm you might suffer will be addressed. The detailed information on this is given in Part 2.

St George's
University of London
Cranmer Terrace
London SW17 0RE
Switchboard
+44 (0)20 8672 9944
www.sgul.ac.uk

STATENS
SERUM
INSTITUT

14 Will my taking part in this study be kept confidential?

Yes. We will follow ethical and legal practice and all information about you will be handled in confidence. The details are included in Part 2.

If the information in Part 1 has interested you and you are considering participation, please read the additional information in Part 2 before making any decision.

St George's
University of London
Cranmer Terrace
London SW17 0RE
Switchboard
+44 (0)20 8672 9944
www.sgul.ac.uk

STATENS SERUM INSTITUT

PART 2

15 What if relevant new information becomes available?

Sometimes we get new information about the drug being studied. If this happens, your research doctor/nurse will tell you and discuss whether you should continue in the study. If you decide not to carry on, you are free to do so. If you decide to continue in the study we may ask you to sign an updated consent form.

If the study is stopped for any other reason, we will tell you and arrange your continuing care.

16 What will happen if I don't want to carry on with the study?

If you withdraw from the study we will need to use the data collected up to your withdrawal. No blood samples will be stored after they have been analysed. A final medical examination will be offered to you.

17 What if there is a problem?

17.1 Complaints

If you have a concern about any aspect of this study, you should ask to speak to the researchers who will do their best to answer your questions (Elka Giemza 020 8725 2316). If you remain unhappy and wish to complain formally, you can do this through the St George's University of London Joint Research Office, details of which can also be obtained from any of the researchers or directly from the St George's web site: www.sgul.ac.uk.

17.2 Harm

If you experience any injuries caused by the study medication, and the study medication has been used in accordance with the study protocol, you will be covered by the sponsor's (Statens Serum Institut, Denmark) "no-fault" product liability insurance under a worldwide liability programme written by the New Hampshire Insurance Company.

If the harm done to you is due to negligence then you may have grounds for a legal action for compensation against St George's University of London, but you may have to pay your own legal costs. The normal National Health Service complaints mechanisms will still be available to you (if appropriate).

18 Will my taking part in this study be kept confidential?

If you choose to join the study, some parts of your medical records and the data collected for the study by the study nurse and the medical doctor will be looked at by authorised persons from the company sponsoring the research (Statens Serum Institut, Denmark), and by representatives of regulatory and ethical authorities to check that the study is being carried out correctly. All will have a duty of confidentiality to you as a research participant and we will do our best to meet this duty.

St George's
University of London
Cranmer Terrace
London SW17 0RE
Switchboard
+44 (0)20 8672 9944
www.sgul.ac.uk

STATENS
SERUM
INSTITUT

After the study is completed your data will be securely stored for 15 years at St. Georges University of London, as advised by the competent authorities in both England and Denmark

19 Involvement of the General Practitioner/Family doctor (GP)

With your permission, we will write to your GP informing them of your participation in the study. We will also send this information sheet so that s/he can understand the principles in the study.

20 What will happen to any samples I give?

The blood samples taken in the study will be analysed at the accredited doctor's laboratory named TDL. All blood samples will be handled with confidentiality and will be destroyed after being analysed.

21 Will any genetic tests be done?

No, no genetic tests will be done.

22 What will happen to the results of the research study?

After the study is completed, the data has been collected and all statistical analyses have been done, the results will be published in a scientific journal.

23 Who is organising and funding the research?

The research study is organised and funded by Statens Serum Institut, Denmark, a non for profit, public institute owned by the Danish state and organised under the Ministry of Health in Denmark. The study is being run by St George's, University of London, and the principal research physician is Prof. David JM Lewis, Centre for Infection at St George's.

24 Who has reviewed the study?

This research is regulated by national and international guidelines. The study has been reviewed and approved by the national competent authorities, MHRA, and in addition all research in the UK is looked at by an independent group of people, called a Research Ethics Committee to protect your safety, rights, wellbeing and dignity. This study has been reviewed and given favourable opinion by Wandsworth Research Ethics Committee.

St George's
University of London
Cranmer Terrace
London SW17 0RE
Switchboard
+44 (0)20 8672 9944
www.sgul.ac.uk

STATENS
SERUM
INSTITUT

25 Further information and contact details

If you seek further information regarding research and research studies, and participating in such studies, we refer to the following internet pages:

- MRC Clinical Trials Unit - Advice for potential participants including lists of trials and questions that people may wish to ask researchers. http://www.ctu.mrc.ac.uk/about_clinical_trials.aspx
- The National Research Register (NRR) Archive - UK database of research projects https://portal.nihr.ac.uk/Pages/NRRArchive.aspx
- National Electronic Library for Health – Information about clinical trials, what they are and why we need them http://www.library.nhs.uk/trials

26 Contact for further information

You will receive a personal study card with full details on how to contact the study staff at any time.

Thank you for taking the time to read this information sheet.

If you consent to participating in the study, you will be given a copy of this information sheet and a copy of the signed consent form for you to keep.

St George's
University of London

Cranmer Terrace
London SW17 0RE
Switchboard
+44 (0)20 8672 9944
www.sgul.ac.uk

Eudract No./Trial Code:<br>**2009-012984-33/ TESEC-02**	Site:<br>**St George's , University of London**
Investigator responsible for site:<br>**Prof. David J M Lewis, MD**	Screening No. / Subject initials:<br>S _ _ _ / _ _ _

Consent Form

Study title:
"A safety and dose finding trial of the diagnostic test C-Tb, when given intradermally by the Mantoux technique to adult patients recently diagnosed with active TB"

**I acknowledge by my initials that:**	Participant initials
➢ I am eligible for free NHS treatment........	
➢ I confirm that I have read and understood the information sheet (version…) for the above study	
➢ I have had the opportunity to consider the information, ask questions, and have had these answered satisfactorily........	
➢ I understand that my participation is voluntary, and that I am free to withdraw at any time, without giving any reason and without my legal rights being affected	
➢ I understand that I will have an HIV test. If the result is positive, I will be excluded from the trial and a physician will advise me about the follow-up and the medical care necessary.	
➢ I understand that relevant sections of any of my medical notes and data collected during the study may be looked at by responsible individuals from the sponsoring company, Statens Serum Institut, DK, from regulatory authorities and/or the ethics committee........	
➢ I understand that the samples being taken are a gift and that they may be used at a later date for future studies but restricted to the analysis of immune responses to the components of the immunisation........	
➢ I agree to take part in the above study........	

Date: _ _ _ _ _ _ _ _   Participant's initials: _ _ _   Investigator/nurse's initials _ _ _

St George's
University of London

Cranmer Terrace
London SW17 0RE
Switchboard
+44 (0)20 8672 9944
www.sgul.ac.uk

.... Name of the participant	.... Date of signature
.... Signature of the participant	

Declaration of the member of the study staff taking consent:

With this I declare that I have informed the above person about the nature, aims, procedures and possible risks involved in participating in this research study. Further, I declare that I have handed over a copy of the Patient Information Sheet to the above person, and that I am prepared to answer all later questions from the participant to the best of my knowledge.

.... Name of the study staff taking consent	.... Date of signature
.... Signature of the study staff taking consent	

Date: | | | | | | | | Participant's initials: | | | Investigator/nurse's initials | | |

Indemnity Statement

Trial title: A safety and dose finding trial of the diagnostic test C-Tb, when given intradermally by the Mantoux technique to adult patients recently diagnosed with active TB

Trial code: TESEC-02

Dear Professor David JM Lewis,

You have kindly agreed to consider undertaking the above-mentioned clinical trial as investigator, in accordance with the protocol for the trial TESEC-02.

In the event that any recruited patient in the trial should suffer any personal injury resulting from the clinical trial, SSI agrees to indemnify the institution where the clinical trial is being undertaken, St George's, University of London, United Kingdom, and, through St George's, University of London, any of its employees or agents participating in the trial, against liability imposed by law, but not assumed voluntarily, and arising from the use of the test products in the trial, PROVIDED THAT:

1) SSI shall not indemnify against, nor have any obligation whatsoever as regards liability arising from or related to any error, omission, intentional wrongful act, or other negligence on the part of said institutions or persons, such as medical malpractice; and

2) Any such institution or person seeking indemnity
   a) has fully complied with the protocol for the trial, and

   b) has promptly notified SSI of any notice of any type of claim, or the likelihood of a claim, relating to the trial,

   c) as regards any claim, makes no statement, takes no action, nor makes any commitment affecting SSI's interests, without SSI's prior written consent, and further, provides all reasonable and necessary assistance to SSI in the defence of any claim, allowing SSI, at its cost and in its discretion, to take over the defence of any action and to have full control in handling the claim.

Please note that this letter is not a legal contract itself, but rather summarizes the main points of SSI's liability under its agreement with St George's, University of London.

Yours sincerely,

Statens Serum Institut
Ingrid Kromann
Head of the Department of Vaccine Development

SUSPECT ADVERSE REACTION REPORT

I. REACTION INFORMATION

1. Subject initials / number	1a. Country	2. Date of birth Day | Month | Year			2a. Age Years	3. Sex F/M	4-6 Reaction onset Day | Month | Year			8-12. Check all appropriate to adverse reaction
/	United Kingdom									☐ Patient died
7+13. Describe reaction(s) and identify reactions that are considered serious. Comment on relatedness and expectedness, intensity, outcome, whether vaccinations were discontinued.										☐ Involved or prolonged inpatient hospitalisation<br>☐ Involved persistent or significant disability or incapacity<br>☐ Life threatening<br>☐ Other medically important condition

II. SUSPECT DRUG(S) INFORMATION

14. Suspect drug(s) name(s) and batch number(s)			20. Did reaction abate after stopping drug? ☐ yes ☐ no ☒ NA
15. Daily dose given of suspect drug(s)<br>**1 dose (0.1 mL) x 2**	16. Route(s) and sites of administration		
17. Indication(s) for use<br>**Diagnosis of Tuberculosis**			21. Did reaction reappear after reintroduction? ☐ yes ☐ no ☒ NA
18. Date(s) of administration of suspect drug(s)		19. Therapy duration<br>N/A	

III. CONCOMITANT DRUG(S) AND HISTORY

22. List relevant past drug history and concomitant drug(s) and dates of administration
23. Other relevant medical history and concurrent conditions.

IV. MANUFACTURER INFORMATION

24a. Name and address of manufacturer<br>**Statens Serum Institut, Artillerivej 5, DK-2300 Copenhagen S, Denmark**		
24b. Manufacturer Control No.	24d. Report source<br>☒ study ☐ literature<br>☐ health professional	Date and signature of initial reporter:<br><br>Print name of investigator:
24c. Date received by manufacturer		
Date of this report	25a. Report type<br>☐ initial ☐ follow-up	Date and signature of reporter of this report:<br><br>Print name of reporter:

Use this page if you have further comments or if additional space is needed for any of the fields of the CIOMS FORM.
Please state No. of the field you continue on this page.

Field No.	Comments or continuation of text
	This field should always be complete<br>Subject initials:<br>Subject number:

AIG

CERTIFICATE OF INSURANCE

This is to certify that a Policy of Insurance as described below has been issued to the Policy Holder and the coverage thereby is as follows:

Master Policy Number: 20001156

Policy Holder: Statens Serum Institut and all their Subsidiary and/or Associated and/or Affiliated Companies as now exist or hereafter may be constituted, formed or acquired as their interests may appear

Type of Insurance: Public and Products Liability

Period of Indemnity: From 1st June 2009 to 31st May 2010 both days inclusive prevailing time at the address of the Insured

Master Limits of Liability: DKK 60,000,000 Combined Single Limit Personal Injury and Property Damage

DKK 60,000,000 Total Annual Aggregate for Products Liability

Retro Date: 1st June 1992

SIR (Self Insured: Retention) (a) United States of America:

DKK.5,000,000 per Occurrence

(b) Elsewhere in the world:

DKK 1,500,000 per Occurrence

Wording: GAP claims made form (amended)

Principal Operations: All operations of the Policy Holder including but not limited to:

(a) the provision of diagnostic services

(b) the manufacture sale and distribution of vaccines and other pharmaceutical products including blood products diagnostic agents and culture media

(c) Research and development

(d) Property Owners

Exclusions: Usual Policy exclusions plus

(a) exclusion of efficacy

(b) total pollution exclusion in respect of United States of America and Canada

Extensions:

(a) Blanket Contractual Liability

(b) Including Clinical Trials Cover

(c) Including Care Custody and Control subject to
i) a sub limit of DKK 10,000,000 any one occurrence and in the aggregate, and
ii) a Self Insured Retention of DKK 500,000

(d) Including Treatment and Processing subject to a Self Insured Retention of DKK 500,000

Nothing herein contained shall in any way be held or construed to vary, alter or waive any of the terms, conditions or provisions of the Policy.

The Policy document is only summarised by this Certificate.

For full information refer to the Policy.

SIGNED FOR AND ON BEHALF OF THE INSURER

DATE: 22 May 2009

AIG UK Limited

This insurance is underwritten by AIG UK Limited (FSA number: 202628) which is authorised and regulated by the Financial Services Authority. Registered in England: company number 1486260. Registered address: The AIG Building, 58 Fenchurch Street, London EC3M 4AB. This information can be checked by visiting the FSA website (www.fsa.gov.uk/register). AIG UK Limited is a member company of American International Group, Inc. (AIG) and a member of the Association of British Insurer
