# Supplementary material for: Randomised Clinical Trial Investigating the Specificity of a Novel Skin Test (C-Tb) for Diagnosis of M. tuberculosis Infection
Source: PLoS One. 2013 May 14;8(5):e64215. doi: 10.1371/journal.pone.0064215 (PMC3653866; doi:10.1371/journal.pone.0064215)
Supplement: Protocol S2 — Trial Protocol. (PDF) [file pone.0064215.s003.pdf]

PROTOCOL

FINAL

Title:

A phase IIa specificity trial of the diagnostic agent C-Tb, when given intradermally by the Mantoux technique to healthy volunteers previously vaccinated with BCG

Trial Code: TESEC-03

EudraCT Number: 2009-017296-17

Trial phase: II

Date of this version:

28-10-2010

Clinical Trial Manager:

Pernille Nyholm Tingskov
Department of Vaccine Development
Statens Serum Institut
Denmark

Confidentiality
This document contains confidential information.
The contents may not be used, divulged or published without prior written consent of Statens Serum Institut.
This information cannot be used for any other purpose than the conduct and evaluation
of the clinical investigation by the investigator(s), regulatory authorities and members of the ethics committee.

1 Synopsis

**Name of sponsor:**<br>Statens Serum Institut<br><br>**Finished product:** C-Tb<br><br>**Name of active ingredient:**<br>Recombinant dimer of 6 kDa early secretory antigen target (rdESAT-6) and recombinant 10 kDa culture filtrate protein (rCFP-10)		**TESEC-03**<br><br>**FINAL Version**

**Title of the trial:**	A phase IIa specificity trial of the diagnostic agent C-Tb, when given intradermally by the Mantoux technique to healthy volunteers previously vaccinated with BCG.
**Investigator:**	Principal Investigator:<br>Professor David JM Lewis<br>Site responsible investigator :<br>Dr Hubert Bland
**Trial centres:**	Clinical Research Centre University of Surrey
**Country:**	United Kingdom
**Trial period:**	Q1 2011 (FPFV) to Q4 2011 (LPLV)
**Trial code /<br>EUDRACT number:**	TESEC-03 / 2009-017296-17
**Trial phase:**	Phase IIa
**Trial objectives:**	**Primary objectives:**<br>• To assess the specificity of the C-Tb test as a function of the cut-off value (i.e., the smallest size of induration measured in mm resulting in a negative outcome of the C-Tb test) when the test is administered intradermally by the Mantoux technique to healthy BCG vaccinated adults<br>The specificity of the C-Tb test is defined as the relative frequency of subjects in a healthy population (i.e., no exposure to MTb) who have an induration responce < cut-off after a C-Tb test.<br>Similarly the sensitivity is defined as the relative frequency of patients with an induration response ≥ cut-off in TB patients.<br>An optimal cut-off point of being infected will be determined by combing the results from the present specificity study with those from a parallel sensitivity study in patients recently diagnosed with TB.<br>**Secondary objectives:**<br>• To compare the induration response of C-Tb with the induration response of 2 T.U. Tuberculin PPD RT 23 SSI<br>• To compare the induration response of C-Tb with the in-vitro IFN-γ response measured at screening and 28 days after the injections of the skin test agents using the QuantiFERON®-TB Gold In-Tube assay<br>• To record all adverse events occurring within 28 days after application of the agents.
**Endpoints:**	**Primary endpoint:**<br>• The diameter of induration at the injection sites measured transversely to the long axis of the forearm 2-3 days after application of the agents

**Name of sponsor:** Statens Serum Institut<br>**Finished product:** C-Tb<br>**Name of active ingredient:** Recombinant dimer of 6 kDa early secretory antigen target (rdESAT-6) and recombinant 10 kDa culture filtrate protein (rCFP-10)		**TESEC-03**<br><br>**FINAL Version**

	**Secondary endpoints:**<br>• Injection site adverse reactions within 28 days after application of the agents<br>• All adverse events occurring within 28 days after application of the agents<br>• Laboratory safety parameters of haematology and biochemistry<br>• *In vitro* IFN-γ response as measured by the QuantiFERON®-TB Gold In-Tube aasay from blood samples taken at screening and 28 days after the injections of the skin test agents.
**Trial design:**	The trial is designed to investigate the specificity of C-Tb under various definitions of cut-off in a double blind randomised, split-body study comparing 0.1 µg/0.1 mL C-Tb with the reference agent 2 T.U. Tuberculin PPD RT23 SSI. (Each volunteer receives the C-Tb agent in one arm and the 2 T.U. Tuberculin PPD in the other arm).<br>The C-Tb and 2 T.U. Tuberculin PPD agents are given concomitantly to each volunteer in the RIGHT AND LEFT forearm according to a double blind randomisation scheme.<br>The volunteers are monitored for adverse events the first hour after administration of the skin test agents (day 0). Follow up visits will take place on days 3 and 28.<br>QuantiFERON®-TB Gold In Tube test will be performed at inclusion (before administration of the skin test agents) and on day 28. If the test result turns positive during the trial the respective volunteers will be asked to return for a follow up visit 6 months after the last visit in the trial to exclude an infection with tuberculosis.
**Trial population:**	Female/male BCG vaccinated adults aged 18 - 65 with a negative IFN-γ result below 0.35 IU/mL at inclusion as measured by the QuantiFERON®-TB Gold In Tube assay.
**Number of volunteers:**	150 healthy male and female volunteers. Slightly more volunteers are expected to enter the trial in order to replace volunteers with a positive or indetermined IFN-γ result.
**Inclusion criteria:**	**The volunteer:**<br>1. Has signed an informed consent<br>2. Aged 18 to 65 years<br>3. Is known to be BCG vaccinated (documented in medical files and/or by the presence of a BCG scar)<br>4. Is healthy according to a medical examination and medical history at screening<br>5. Is willing and likely to comply with the trial procedures<br>6. Is prepared to grant authorized persons access to their medical records

**Name of sponsor:** Statens Serum Institut<br>**Finished product:** C-Tb<br>**Name of active ingredient:** Recombinant dimer of 6 kDa early secretory antigen target (rdESAT-6) and recombinant 10 kDa culture filtrate protein (rCFP-10)		**TESEC-03**<br><br>**FINAL Version**

Exclusion criteria:

The volunteer:

1. Has a history of tuberculosis or has been in close contact to a person with active tuberculosis within 5 years from trial inclusion
2. Has a positive QuantiFERON®-TB Gold In-Tube assay at inclusion
3. Laboratory parameters outside of normal range judged by site investigator to be clinically significant
4. Has within 3 months prior to the day of inclusion been in treatment with a product which is likely to modify the immune response (e.g., immunoglobulin, systemic corticosteroids, methotrexate, azathioprine, cyclosporine or blood products)
5. Has been vaccinated with a live vaccine within 6 weeks prior to the day of inclusion (e.g. MMR, yellow fever, oral typhoid vaccines)
6. Has been vaccinated with BCG < 6 months prior to the day of inclusion
7. Has been tuberculin (TST) tested < 6 months prior to the day of inclusion
8. Has a known congenital or acquired immune deficiency
9. Has an active disease affecting the lymphoid organs (e.g., Hodgkin's disease, lymphoma, leukaemia, sarcoidosis)
10. Is infected with HIV
11. Has a current skin condition which interferes with the reading of the skin tests e.g. tattoos, severe scarring, burns/sunburns, rash, eczema, psoriasis, or any other skin disease at or near the injection sites
12. Has a condition where blood drawings pose more than minimal risk for the volunteer, such as haemophilia, other coagulation disorders, or significantly impaired venous access
13. Currently participating in another clinical trial with an investigational or non investigational drug or device, or has participated in another clinical trial within the 3 months prior to dosing
14. Has participated in previous clinical trials investigating the ESAT-6 and/or CPP-10 antigens
15. Is pregnant, breast-feeding or intending to get pregnant
16. Is a female not willing to use effective barrier (including spermicidal gel), hormonal or intrauterine contraceptive measures
17. Has a history of alcohol, narcotic, benzodiazepine, or other substance abuse or dependence within the 12 months preceding Visit 1
18. Has a positive urine drug screen at Visit 1 and Visit 2 (i.e., amphetamines, barbiturates, benzodiazepines, cannabinoids, cocaine, or opiates)
19. Has a positive alcohol breath test at Visit 1 and Visit 2. [NOTE: subjects must be told to avoid consumption of alcoholic beverages for at least 24 hours prior to attending the Centre]
20. Has a condition which in the opinion of the investigator is not suitable for participation in the study

<table>
<tr><td>Name of sponsor:
Statens Serum Institut</td><td rowspan="3"></td><td rowspan="3">TESEC-03

FINAL Version</td></tr>
<tr><td>Finished product: C-Tb</td></tr>
<tr><td>Name of active ingredient:
Recombinant dimer of 6 kDa early secretory antigen target (rdESAT-6) and recombinant 10 kDa culture filtrate protein (rCFP-10)</td></tr>
<tr><td>Investigational diagnostic agent:</td><td colspan="2">Investigational product C-Tb (rdESAT-6 + rCFP-10):
rdESAT-6 .... 0.5 µg
rCFP-10 .... 0.5 µg
Disodium hydrogen phosphate dehydrate .... 1.4 mg
Potassium dihydrogen phosphate .... 0.2 mg
Potassium chloride .... 0.2 mg
Sodium chloride .... 8.0 mg
Polysorbate 20 .... 0.1 µL
Phenol .... 0.5 %
Water for injections .... up to 1 mL</td></tr>
<tr><td>Active reference diagnostic agent:</td><td colspan="2">2 T.U. Tuberculin PPD RT 23 SSI – 0.1 mL</td></tr>
<tr><td>Dosage and route of administration:</td><td colspan="2">0.1 mL of the investigational diagnostic agent (C-Tb) is administered to the RIGHT or LEFT forearm as indicated by the randomisation code and 0.1 mL of the reference agent (2 TU PPD) is administered to the opposite forearm.
The two injections will be administered intradermally by the Mantoux technique into the flexor surface of the RIGHT/LEFT forearm at the junction of the upper third with the lower two-thirds. A 1 mL syringe fitted with a short bevelled needle (insulin needle) is used for the injection of the skin test agents.
The w:w ratio of rdESAT-6 and rCFP-10 (C-Tb) is 1:1 in the solution. A dose of 0.1 µg C-Tb refers to a test solution consisting of 0.05 µg rdESAT-6 and 0.05 µg rCFP-10 per 0.1 mL.</td></tr>
<tr><td>Statistical methods:</td><td colspan="2">The main objective of the trial is to contribute information on the size of induration in a healthy BCG vaccinated population, which can be used to define an optimal cut-off point for a C-Tb skin test.
In the trial the specificity of C-Tb ($Spec_x$) as a function of x (the diameter of the induration in mm) will be estimated.
For each (integer) value of x, $Spec_x$ is estimated by the relative frequency of volunteers in the trial with a C-Tb induration $\leq$ x mm. 95% confidence intervals for $Spec_x$ are obtained as $Spec_x \pm 2 \times \sqrt{(Spec_x \times (1 - Spec_x) / n)}$, where n is the number of validly included volunteers.
As BCG vaccinated healthy subjects are not supposed to get an induration when exposed to C-Tb no formal statistical comparison of induration sizes between C-Tb and PPD will be made.
The subjects will have their PPD indurations classified according to UK standards for PPD cut-off points: 0-5 mm, 6-14 mm and 15+ mm (The Green Book).
PPD is considered negative if the induration is < 6 mm.
www.dh.gov.uk/en/Publichealth/Healthprotection/Immunisation/Greenbook/DH_4097254</td></tr>
</table>

2 Table of Contents

1 Synopsis.......... 2

2 Table of Contents.......... 6

3 List of Abbreviations and Definitions.......... 9

4 Signature page.......... 11

5 Relevant addresses.......... 12

6 Introduction.......... 15

7 Trial objectives.......... 18

7.1 Primary objectives.......... 18

7.2 Secondary objectives.......... 19

7.3 Primary endpoint.......... 19

7.4 Secondary endpoints.......... 19

8 Investigational plan.......... 19

8.1 Overall design.......... 19

8.2 Study population and organisation of the clinical trial.......... 20

8.3 Number of subjects.......... 21

8.4 Recruitment.......... 21

8.5 Inclusion criteria.......... 21

8.6 Exclusion criteria.......... 21

8.6.1 Predetermined reasons for discontinuation.......... 22

8.6.2 Permanent contraindications.......... 23

8.6.3 Temporary contraindications.......... 23

9 Investigational medicinal products.......... 23

9.1 Composition.......... 23

9.2 Treatments administered.......... 24

9.2.1 Doses and administration.......... 24

9.2.2 Packaging and labelling.......... 25

9.2.3 Transport of trial vaccines.......... 25

9.2.4 Storage information.......... 26

9.2.5 Randomisation procedure.......... 26

9.2.6 Blinding and unblinding.......... 27

9.2.7 Treatment compliance.......... 28

9.2.8 Drug accountability.......... 28

9.2.9 Precautions and overdosing.......... 28

9.2.10 Concomitant medication 29
9.3 Investigational events 30
9.3.1 Specificity assessment 31
9.3.2 Safety assessments 31
9.3.3 Collection and handling of blood samples 32
9.3.4 Time schedule 33
10 Ethical aspects 33
10.1 Payment of volunteers 34
10.2 Data Safety Monitoring Board 35
11 Adverse events 35
11.1 Definitions and terms 35
11.2 Standard reporting of adverse events 36
11.3 Investigator's immediate reporting of serious adverse events to SSI 37
11.4 SSI's expedited reporting of SUSARs to CA and EC 38
11.5 Annual reporting of suspected serious adverse reactions to CA and EC 39
12 Data management and statistical analysis 39
12.1 General considerations 39
12.2 Data management 40
12.3 Clean file procedures 40
12.4 Analysis populations 40
12.5 Statistical methods 41
12.6 Sample size determinations 41
12.7 Interim analysis 41
13 Good clinical practice considerations 42
13.1 Declaration of Helsinki 42
13.2 Subject information and informed consent 42
13.3 Independent ethics committee submission and approval 42
13.4 Competent authority submission and approval 42
13.5 Subject data protection 43
13.6 Investigator's responsibilities 43
13.7 Curricula vitae and log(s) of staff 43
13.8 Indemnity statement 44
13.9 Training 44
13.10 Monitoring 44
13.11 Audit and inspection 45

13.12 Definition and archiving of source data 45
13.13 Definition and archiving of essential documents 46
14 Agreement and financial settlement 46
15 Insurance 46
16 Confidentiality and disclosure 46
17 Changes to the protocol 47
18 References 47

Appendices

Appendix 1: Declaration of Helsinki
Appendix 2: Indemnity Statement
Appendix 3: CIOMS form
Appendix 4: Insurance certificate

3 List of Abbreviations and Definitions

ADR	Adverse Drug Reaction
AE	Adverse Event
AFB	Acid fast bacteria
ALP	Alkaline Phosphatase
ALT	Alanine aminotransferase
AR	Adverse Reaction
AST	Aspartate aminotransferase
BCG	Bacille Calmette-Guérin
CA	Competent Authority
CFP-10	10 kDa Culture Filtrate Protein
CI	Confidence Interval
CIOMS	Council for International Organizations of Medical Sciences
CRA	Clinical Research Associate
CRC	Clinical Research Centre
CRF	Case Record Form
CTA	Clinical Trial Application
C-Tb	rdESAT-6 + rCFP10
Cut-off	The smallest induration diameter (mm) resulting in a positive C-Tb test
DOB	Date of Birth
DTH	Delayed Type Hypersensitivity
DSMB	Data safety monitoring board
DSMP	Data safety monitoring plan
EC	Ethics Committee
ESAT-6	6 kDa Early Secretory Antigenic Target
FPFV	First Volunteer First Visit
GCP	Good Clinical Practice
GMP	Good Manufacturing Practice
HIV	Human Immunodeficiency Virus
IB	Investigators Brochure
ICH-GCP	The International Conference on Harmonisation of Good Clinical Practice
IMPD	Investigational medicinal product dossier
IF	Investigators File
IFN-γ	Interferon gamma
LPLV	Last Volunteer Last Visit
MHRA	Medicines and Healthcare products Regulatory Agency
MTb	*Mycobacterium tuberculosis*
NCS	Not clinically significant

Ph. Eur	The European Pharmacopoeia
PPD	Purified Protein Derivative
PPD RT 23	Purified Protein Derivative Rinsed Tuberculin (batch) 23
QFT	QuantiFERON®-TB Gold In Tube assay
QP	Qualified Person
QPPV	Qualified Person for Pharmacovigilance
rCFP-10	Recombinant 10kDa Culture Filtrate Protein
rdESAT-6	Recombinant dimer of 6 kDa Early Secreted Antigen Target
RBC	Red blood cell count
REC	Research Ethics Committee
RGN	Registered General Nurse
ROC	Receiver operating characteristic
SAE	Serious Adverse Event
SAR	Suspected adverse (drug) reaction
Sensitivity	The probability that a test result is positive given the subject has the disease [1]
SmPC	Summaries of Product Characteristics
Specificity	The probability that a test result is negative given the subject does not have the disease [1]
SSI	Statens Serum Institut, Denmark
SSAR	Serious Suspected Adverse Reaction
SUSAR	Suspected Unexpected Serious Adverse (drug) Reaction
TB	Tuberculosis
TDL	The Doctor's Laboratory
TMF	Trial master file
TST	Tuberculin Skin Test
WBC	White blood cell count
WHO	World Health Organisation

4 Signature page

Clinical Trial Manager, SSI:	Pernille Nyholm Tingskov	
		(date & signature)
Principal Investigator, University of Surrey:	David JM Lewis	
		(date & signature)
Site responsible investigator, University of Surrey:	Hubert Bland	
		(date & signature)
Project Manager, SSI:	Henrik Aggerbeck	
		(date & signature)
Trial statistician, SSI:	Anders Mørup Jensen	
		(date & signature)
Medically responsible, SSI:	Trine R. Nielsen	
		(date & signature)
Manager Clinical Trial Unit, SSI:	Erik Carp	
		(date & signature)
Sponsor's representative, SSI:	Ingrid Kromann	
		(date & signature)

5 Relevant addresses

Principal Investigator, University of Surrey:	Prof. David JM Lewis, MD<br>Division of Clinical Medicine<br>Faculty of Health & Medical Sciences<br>Surrey Clinical Research Centre<br>Egerton Road<br>University of Surrey<br>Guildford, Surrey GU2 7XP	Tel: +44 20 87255826<br>Fax: +44 20 87253487<br>e-mail: sgjf300@sgul.ac.uk
Director Clinical Research Centre, University of Surrey:	Julia Boyle BSc (Hons) PhD<br>Division of Clinical Medicine<br>Faculty of Health & Medical Sciences<br>Surrey Clinical Research Centre<br>Egerton Road<br>University of Surrey<br>Guildford, Surrey GU2 7XP	Tel: +44 (0)1483 689783<br>Fax: +44 (0)1483 689790<br>e-mail: J.Boyle@surrey.ac.uk
Site Responsible Investigator, Medical Director Clinical Research Centre, University of Surrey	Dr Hubert Bland, MBChB<br>Division of Clinical Medicine<br>Faculty of Health & Medical Sciences<br>Surrey Clinical Research Centre<br>Egerton Road<br>University of Surrey<br>Guildford, Surrey GU2 7XP	Tel: +44 (0)1483 689797<br>Fax: +44 (0)1483 689790<br>e-mail: h.bland@surrey.ac.uk
Study nurse Clinical Research Centre, University of Surrey	James Woolley<br>Division of Clinical Medicine<br>Faculty of Health & Medical Sciences<br>Surrey Clinical Research Centre<br>Egerton Road<br>University of Surrey<br>Guildford, Surrey GU2 7XP	Tel: +44 (0)1483 683724<br>Fax: +44 (0)1483 689790<br>e-mail: James.Woolley@surrey.ac.uk
Project Manager Clinical Research Centre, University of Surrey	Jo Arthur<br>Division of Clinical Medicine<br>Faculty of Health & Medical Sciences<br>Surrey Clinical Research Centre<br>Egerton Road<br>University of Surrey<br>Guildford, Surrey GU2 7XP	Tel: +44 (0)1483 689788<br>Fax: +44 (0)1483 689790<br>e-mail:j.arthur@surrey.ac.uk

Project Manager, SSI:	Henrik Aggerbeck<br>Statens Serum Institut<br>Department of<br>Vaccine Development<br>5 Artillerivej<br>DK-2300 Copenhagen S	Tel: +45 3268 3486<br>Fax: +45 3268 3872<br>e-mail: hea@ssi.dk
Monitor, SSI:	Christine Dam Bendiksen<br>Statens Serum Institut<br>Department of<br>Vaccine Development<br>5 Artillerivej<br>DK-2300 Copenhagen S	Tel: +45 3268 3846<br>Fax: +45 3268 3872<br>e-mail: cdb@ssi.dk
Clinical Trial Manager, SSI:	Pernille Nyholm Tingskov<br>Statens Serum Institut<br>Department of<br>Vaccine Development<br>5 Artillerivej<br>DK-2300 Copenhagen S	Tel: +45 3268 3416<br>Fax: +45 3268 3872<br>e-mail: pnt@ssi.dk
Trial statistician, SSI:	Anders Mørup Jensen<br>Statens Serum Institut<br>Biostatistics Unit<br>5 Artillerivej<br>DK-2300 Copenhagen S	Tel: +45 3268 3284<br>Fax: +45 3268 3886<br>e-mail: amo@ssi.dk
Medically responsible, SSI:	Trine R. Nielsen<br>Statens Serum Institut<br>Department of Regulatory &<br>Medical Affairs<br>5 Artillerivej<br>DK-2300 Copenhagen S	Tel: +45 3268 3436<br>Fax: +45 3268 3973<br>e-mail: trn@ssi.dk
QPPV, SSI	Michael Andersen Stevner<br>Statens Serum Institut<br>Department of Regulatory &<br>Medical Affairs<br>5 Artillerivej<br>DK-2300 Copenhagen S	Tel: +45 3268 3916<br>Fax: +45 3268 3973<br>e-mail: msr@ssi.dk
Sponsor's representative, SSI:	Ingrid Kromann<br>Statens Serum Institut<br>Department of<br>Vaccine Development<br>5 Artillerivej<br>DK-2300 Copenhagen S	Tel: +45 3268 8252<br>Fax: +45 3268 3872<br>e-mail: ikr@ssi.dk

Manager Clinical Trial Unit, SSI:	Erik Carp<br>Statens Serum Institut<br>Department of Vaccine Development<br>5 Artillerivej<br>DK-2300 Copenhagen S	Tel: +45 3268 8657<br>Fax: +45 3268 3872<br>e-mail: eca@ssi.dk

Emergency telephone numbers and e-mail addresses:

Clinical Trial Manager, SSI:	Pernille Nyholm Tingskov	Office: +45 3268 3416<br>Mobile: +45 5176 1222<br>Private: +45 3537 8720
Project Manager, SSI:	Henrik Aggerbeck	Office: +45 3268 3486<br>Mobile: +45 5192 6098
Monitor, SSI:	Christine Dam Bendiksen	Office: +45 3268 3846<br>Mobile: +45 2030 3263<br>Private: +45 3534 2636
Medically responsible, SSI:	Trine R. Nielsen	Office: +45 3268 3436<br>Mobile: +45 4131 8179
Serious Adverse Event Notification to SSI:<br><br>(Only if fax or phone is not possible):		Fax: +45 3268 3973<br>24-hour Mobile: +45 2567 0914<br><br>(e-mail: clin.trial@ssi.dk)

6 Introduction

Tuberculosis (TB) continues to be a major cause of morbidity and mortality throughout the world. The disease is caused by infection with *Mycobacterium tuberculosis* (MTb), an intracellular pathogen with one of the most devastating global impacts. WHO estimates that 9.4 million new cases of TB occurred in 2008 with an estimated 1.3 million deaths in HIV negative people and an additional 500 000 deaths among HIV-positive people [2, 3, 4].

One of the 'Millennium Development Goals' from the United Nations Development Programme [5] is to halt and begin to reverse the incidence of TB by 2015. One of the critical issues in achieving this goal is to control the disease by preventing infection and treating the disease as early as possible [6]. Fast and accurate diagnostics have a very important role in this; however in many clinical situations this is not possible as the existing diagnostic methods have serious limitations [7]. Worldwide the majority of TB patients are still diagnosed with tools developed in the 19th Century such as the Tuberculin Skin Test (TST) using Purified Protein Derivative (PPD) and sputum microscopy [6, 8, 9]. The drawback of PPD is the fact that its protein components are shared by many non-tuberculous/environmental mycobacterium families commonly found in developing countries, as well as by the Bacille Calmette-Guérin (BCG) vaccine strains. This significantly decreases the specificity of the TST, since individuals exposed to non-tuberculous mycobacteria or vaccinated with BCG respond immunologically to PPD as well as those who are infected with the tuberculous families (*M. tuberculosis*, *M. bovis*, or *M. africanum*) [10].

MTb recovered from human specimens by microbial culture is today still the gold standard for diagnosing active TB, however the growth of MTb may take from 2 weeks to 2 months and clinicians therefore need to rely on other methods to diagnose patients and initialize treatment [6, 11].

Weaknesses of today's diagnostic tools lead in general to untreated TB cases, to treatment of patients not infected with MTb, waste of resources and complications from the side effects of anti TB treatment [6]. The development of robust diagnostic tests for detecting latent and active TB is therefore urgently needed.

The tuberculous bacteria contain few specific proteins/antigens that are not present in BCG or most environmental mycobacteria. The ESAT-6 (6 kD Early Secreted Antigen Target) protein and CFP-10 (10 kDa culture filtrate protein) were identified from a *M. tuberculosis* culture filtrate [12, 13]. Both antigens, ESAT-6 and CFP-10 are expressed by the tuberculous mycobacterium families (*M. tuberculosis, M. bovis, and M. africanum*) but not by any of the BCG-strains and only by few of the atypical mycobacteria (e.g., *M. kansasii, M. marinum, M. szulgai*) [10, 13].

Based on this knowledge new *in-vitro* diagnostic tests have been developed in the form of T-cell based interferon-γ (IFN-γ) assays. These assays use MTb specific antigens (e.g. ESAT-6, CFP-10) and show that T-cells of individuals infected with MTb produce IFN-γ when they are exposed to these antigens [14]. The tests show very promising abilities in detecting latent

infection with MTb and were shown to be able to discriminate between patients infected by MTb and BCG vaccinated individuals [13, 15, 16, 17, 18, 19, 20]. The impact of these assays in resource limited countries will however be small due to cost, complexity and the lack of local laboratory infrastructure. [6].

A new tuberculin-like skin test named C-Tb, using a combination of rdESAT-6 and rCFP-10 antigens, has been developed by SSI, Denmark, as a diagnostic test for detecting *M. tuberculosis* infection in man. Following intradermal injection of C-Tb to MTb infected guinea pigs an immune response was formed at the injection site visualised as a cutaneous induration and erythema in TB infected animals. The response is known as local delayed type hypersensitivity (DTH) skin reactions [13, 21, 22]. Both antigens are produced in *Lactococcus lactis*.

The first in man trial with rdESAT-6 alone (TESAT-01) was completed in 2005/2006. It was a dose escalating phase Ia clinical trial conducted at Leiden University Medical College, the Netherlands with 4 doses of 0.01 µg, 0.1 µg, 1 µg and 10 µg administered to groups of healthy volunteers and later 3 doses of 0.01 µg, 0.1 µg and 1 µg were tested in groups of previously treated TB-patients. The aim of this first in man phase I trial was to investigate the safety and the diagnostic potential of rdESAT-6 and to compare it with 2 T.U. Tuberculin PPD RT 23 SSI. The immune response induced in previously treated TB patients was here used as a surrogate marker for a latent infection.

Twenty healthy adults were tested. The rdESAT-6 skin test seemed to be safe in healthy adults in the tested dose range (0.01 to 10 µg). Fifteen previous tuberculosis patients were tested. In this population, 0.01 and 0.1 µg rdESAT-6 seemed safe, whereas, administration of 1.0 µg seemed to be related to more intense and more frequent local reactions at the rdESAT-6 injection site. Only few systemic adverse events were reported, most of these were mild and unrelated to the test administrations.

Based on the results of this study, it was concluded that 0.01 µg and 0.1 µg rdESAT-6 were well tolerated and induced responses at 72± 24 hours of a similar size to 2 T.U. Tuberculin PPD RT 23 SSI. The trial indicated a human dose of rdESAT-6 to be in the range of 0.01 to 0.1 µg/0.1 mL [23, 24]. A dose of 0.1 µg rdESAT-6 was therefore selected for the next phase Ib clinical trial.

Pharmacology studies in guinea pigs showed a risk of sensitisation upon repeated injections of rdESAT-6 visualized by local induration and erythema at the injection site. To address this issue a phase Ib trial (TESAT-02) was conducted at Rigshospitalet, Copenhagen in 2007 to assess the risk of sensitisation upon repeated injections of a fixed dose level of 0.1 µg rdESAT-6 with time intervals of 28, 56 or 112 days, respectively.

31 healthy adults were tested. No serious adverse events occurred and only few adverse events were reported by investigator and all assessed as mild in intensity. The results of this study supported that 0.1 µg rdESAT-6 was safe and well tolerated as seen in the previous phase I study. The results showed a risk of inducing a false positive skin reaction if 0.1 µg rdESAT-6

was injected twice four weeks apart however this risk of sensitisation seemed to decrease with and increased time span between succeeding testing as suggested by previous animal studies [25, 26].

To increase the potential sensitivity of the skin test, experience with IFN-γ assays showed that combinations with two or more antigens were necessary for a sufficient sensitivity. As a consequence of these considerations SSI decided in March 2007 to include rCFP-10 to the rdESAT-6 skin test agent without jeopardizing its predicted specificity [13]. The product was named C-Tb. Non-clinical pharmacology studies at SSI confirmed in guinea pigs that the sensitivity increased when rdESAT-6s was mixed with rCFP-10 and similar dose response curves were observed with rdESAT-6 and rCFP-10.

In 2005 Reece et al [27] published that CFP-10 may trigger “Tuberculin shock”. He found that 5/10 guinea pigs died 6-36 hours after the skin testing with CFP-10, if the test was done 6 weeks after infection. No deaths were observed if the skin testing was done 4 weeks after infection or earlier. A more likely explanation is that the testing and the deaths were coincidental as the guinea pigs after 6 weeks of infection were at a late stage of disease, where mortality is not infrequent. In a repeat study, no. F1137 [28], conducted at SSI with an infection period of 6 weeks before testing 3/30 (10%) animals died or were about to die from TB even before skin testing. The same number of animals died after skin testing with rCFP-10 or PPD. It is likely that any immunological reagent at this time of disease will be able to induce a shock-like syndrome in an animal.

A bridging toxicity study performed in 2008 with C-Tb concluded that repeated subcutaneous injections of 10 µg C-Tb were safe in rats.

The first clinical trial with C-Tb (TESEC-01) was designed to investigate the safety and the risk of sensitisation upon repeated injections of C-Tb in healthy adult volunteers. C-Tb was investigated at two doses of 0.01 and 0.1 µg C-Tb. The two doses were injected twice 6 or 12 weeks apart to investigate a risk of sensitisation upon repeated testing. This phase Ia trial was conducted at Rigshospitalet, Copenhagen. No serious adverse events occurred and only few mild adverse events were reported in this first in man clinical trial with C-Tb. Further, it was demonstrated that a second injection of C-Tb can be injected after 6 weeks without inducing a false positive skin reaction. If the time from infection to testing is shorter than the incubation period of TB (=6 weeks) a false negative response to the skin test agents may occur. To rule out an infection during contact tracing and to allow an immune response to develop dual testing 6 weeks apart may therefore be of relevance to a future TB diagnostic skin test [29, 30].

A randomised phase Ib trial (TESEC-02) was initiated in Great Britain in Q1 2010 to determine a safe human dose of C-Tb. As there is no gold standard to identify latent infection, the study was done in individuals within 60 days of starting treatment for active TB. It was anticipated that individuals at an early stage of treatment for active disease may give stronger adverse reactions to C-Tb than individuals with a waning immunity from an infection cured several years before. To be able to market C-Tb in multi-dose vials it is a requirement that the product is preserved. Unpreserved and preserved (0.5 % phenol) formulations of C-Tb were randomly

injected into the left or right arm of each volunteer in a double blind safety and dose finding trial to allow a direct investigation if phenol affects the safety profile and the response of C-Tb.

This trial concluded that C-Tb preserved with 0.5% phenol was safe in patients with active TB and that a dose of 0.1 µg C-Tb gave a skin reaction of a desired size. It was found acceptable to proceed to phase II trials to determine an optimal cut-off point for C-Tb as sign of a TB infection and thus allow calculation of sensitivity and specificity.

The present phase IIa (TESEC-03) trial in a healthy BCG vaccinated population will collect data on the distribution of the induration response, if any, to C-Tb in this population. The specificity of the C-Tb test will be defined as the relative frequency of subjects in a healthy population (i.e., no exposure to MTb) who have an induration responce < cut-off after a C-Tb test.

A parallel phase IIb trial (TESEC-04) is planned in patients recently diagnosed with TB +/- HIV to collect data on the distribution of the induration response to C-Tb in these populations. The sensitivity is defined as the relative frequency of patients with an induration response ≥ cut-off in TB patients. The phase IIb clinical trial will be conducted in South Africa in parallel to this phase IIa trial in the United Kingdom.

The optimal cut-off point of being infected will be determined by combing the results from the present specificity study in BCG vaccinated with no previous exposure to MTb with those from a parallel sensitivity study in recently diagnosed TB infected individuals +/- HIV via a Receiver operating characteristic curve.

It is expected that the healthy BCG vaccinated volunteers to be enrolled in the present phase IIa trial will only be subjected to minimal risks of local and systemic adverse reactions following a single intradermal injection of 0.1 µg C-Tb by the Mantoux injection technique. This is based on the non-clinical pharmacology and toxicity studies, previous phase I clinical trials with rdESAT-6 (alone) [23, 24, 25, 26] and two phase I trials with C-Tb, respectively [29, 30].

This protocol has been written in accordance with ICH GCP Topic E 6 [31], ICH Topic E 2A [32] and the EC directives 2001/20/EC [33] and 2005/28/EC [34] of the European Parliament (including underlying guidance).

7 Trial objectives

7.1 Primary objectives

– To assess the specificity of the C-Tb test as a function of the cut-off value (i.e., the smallest size of induration measured in mm resulting in a positive outcome of the C-Tb test) when the test is administered intradermally by the Mantoux technique to healthy BCG vaccinated adults.

The specificity of the C-Tb test is defined as the relative frequency of subjects in a healthy population (i.e., no exposure to MTb) who have an induration response < cut-off after a C-Tb

test, whereas the sensitivity is defined as the relative frequency of patients with an induration response ≥ cut-off in TB patients.

7.2 Secondary objectives

- To compare the induration response of C-Tb with the induration response of 2 T.U. Tuberculin PPD RT 23 SSI

- To compare the induration response of C-Tb with the in-vitro IFN-γ response measured at screening and 28 days after the injections of the skin test agents using the QuantiFERON®-TB Gold In-Tube assay

- To record all adverse events occurring within 28 days after application of the agents

7.3 Primary endpoint

- The diameter of induration at the injection sites measured transversely to the long axis of the forearm 2-3 days after application of the agents

7.4 Secondary endpoints

- Injection site adverse reactions within 28 days after application of the agents

- All adverse events occurring within 28 days after application of the agents

- Laboratory safety parameters of haematology and biochemistry

- *In vitro* IFN-γ response as measured by the QuantiFERON®-TB Gold In-Tube assay from blood samples taken at screening and 28 days after the injections of the skin test agents

8 Investigational plan

8.1 Overall design

This clinical trial is a single-centre phase IIa study designed to investigate the specificity of C-Tb under various definitions of cut-off in a double blind randomised, split-body study comparing 0.1 µg/0.1 mL C-Tb with the reference agent 2 T.U. Tuberculin PPD RT 23 SSI (Each volunteer receives the C-Tb agent in one arm and the 2 T.U. Tuberculin PPD RT 23 SSI agent in the other arm). The two skin tests are administered in a double-blind manner to make topical readings of an induration, if any, as objective as possible.

The trial will be conducted at Surrey Clinical Research Centre, University of Surrey, Guildford, United Kingdom under the responsibility of Principal Investigator: Professor David JM Lewis.

The primary objective of the trial is to assess the specificity of the C-Tb skin test agent defined as the relative frequency of subjects in a healthy population (i.e., no exposure to MTb) who have an induration response < cut-off after a C-Tb test.

The C-Tb skin test is developed from MTb specific antigens (ESAT-6 and CFP-10) which are not expressed by any of the BCG-strains and only by a few of the atypical mycobacteria (e.g., *M. kansasii*, *M. marinum*, *M. szulgai*). No skin reaction (induration) is therefore expected at the injection sites with C-Tb. PPD, however, has protein components that are shared by the BCG vaccine strains and many non-tuberculous/ environmental mycobacteria. Some skin reactions are therefore expected to occur after testing with 2 T.U. Tuberculin PPD RT 23 SSI in a trial population of BCG vaccinated individuals.

Both erythema and induration, if any, will be documented in the case record forms (CRFs) of the volunteers.

In addition to the above, the immune response will be evaluated together with an assessment of the safety of the C-Tb skin test agent.

The volunteers are monitored closely for the occurrence of immediate adverse reactions during the first hour after the injections.

Each trial volunteer will be followed 28 days after the skin test injections and is expected to complete a total of 4 trial visits, as follows:

**Visit 1**	-	*Screening Visit* - comprising a medical examination and blood tests for clinically safety parameters and the QuantiFERON®-TB Gold In-Tube
**Visit 2**	**Day 0**	*Inclusion Visit and Injection of skin tests* (0-28 days after screening)
**Visit 3**	**Day 2-3**	*Assessment Visit*
**Visit 4**	**Day 28**	*Final 28 day Visit* - comprising a medical examination and blood tests for clinically safety parameters (± 2 days)

For practical reasons Visit 2 (Inclusion and injection) should always take place on a Monday, Tuesday, Wednesday or Friday (to allow follow-up 2 or 3 days after skin testing). For more details on the visits see Section 9.3.

QuantiFERON®-TB Gold In-Tube assay will be performed at the screening visit and on day 28. There is a small risk that this blood test can become positive during the trial due to the injected C-Tb skin test agent. Volunteers with a positive QFN test taken at the last visit (Visit 4) will therefore be offered an additional visit 6 months after the last visit in the trial to be re-tested with the QFN assay.

8.2 Study population and organisation of the clinical trial

Female/male BCG vaccinated adults aged 18 - 65 with an IFN-γ result below 0.35 IU/mL at inclusion.

All volunteers will be recruited by the Surrey Clinical Research Centre, University of Surrey, England.

More centres may be included.

8.3 Number of subjects

150 healthy BCG vaccinated male and female volunteers between 18 and 65 years of age.

At least 20 % of all included subjects must be < 40 years of age.

At least 20% of all included subjects must be ≥ 40 years of age.

8.4 Recruitment

Volunteers will be recruited by the Surrey Clinical Research Centre, University of Surrey. Recruitment will be via approved advertisements/posters in paper, internet websites, Volunteer Recruitment Database, University of Surrey campus, email, SMS contact and radio as required.

8.5 Inclusion criteria

The volunteer:

1. Has signed an informed consent
2. Aged 18 to 65 years
3. Is known to be BCG vaccinated (documented in medical files and/or by the presence of a BCG scar)
4. Is healthy according to a medical examination and medical history at screening
5. Is willing and likely to comply with the trial procedures
6. Is prepared to grant authorized persons access to their medical records

8.6 Exclusion criteria

The volunteer:

1. Has a history of tuberculosis or has been in close contact to a person with active tuberculosis within 5 years from trial inclusion
2. Has a positive QuantiFERON®-TB Gold In-Tube assay at inclusion
3. Laboratory parameters outside of normal range judged by site investigator to be clinically significant
4. Has within 3 months prior to the day of inclusion been in treatment with a product which is likely to modify the immune response (e.g., immunoglobulin, systemic corticosteroids, methotrexate, azathioprine, cyclosporine or blood products)
5. Has been vaccinated with a live vaccine within 6 weeks prior to the day of inclusion (e.g. MMR, yellow fever, oral typhoid vaccines)

6. Has been vaccinated with BCG < 6 months prior to the day of inclusion

7. Has been tuberculin (TST) tested < 6 months prior to the day of inclusion

8. Has a known congenital or acquired immune deficiency

9. Has an active disease affecting the lymphoid organs (e.g., Hodgkin's disease, lymphoma, leukaemia, sarcoidosis)

10. Is infected with HIV

11. Has a current skin condition which interferes with the reading of the skin tests e.g. tattoos, severe scarring, burns/sunburns, rash, eczema, psoriasis, or any other skin disease at or near the injection sites

12. Has a condition where blood drawings pose more than minimal risk for the volunteer, such as haemophilia, other coagulation disorders, or significantly impaired venous access

13. Currently participating in another clinical trial with an investigational or non investigational drug or device, or has participated in another clinical trial within the 3 months prior to dosing

14. Has participated in previous clinical trials investigating the ESAT-6 and/or CPP-10 antigens

15. Is pregnant, breast-feeding or intending to get pregnant

16. Is a female not willing to use effective barrier (including spermicidal gel), hormonal or intrauterine contraceptive measures

17. Has a history of alcohol, narcotic, benzodiazepine, or other substance abuse or dependence within the 12 months preceding Visit 1

18. Has a positive urine drug screen at Visit 1 and Visit 2 (i.e., amphetamines, barbiturates, benzodiazepines, cannabinoids, cocaine, or opiates)

19. Has a positive alcohol breath test at Visit 1 and Visit 2. [NOTE: subjects must be told to avoid consumption of alcoholic beverages for at least 24 hours prior to attending the Centre]

20. Has a condition which in the opinion of the investigator is not suitable for participation in the study

8.6.1 Predetermined reasons for discontinuation

Trial volunteers are free to withdraw from the trial whenever they desire without giving a reason for this and will still be entitled to continuing medical care.

There will be no pre-defined medical events, which could lead to the withdrawal of a volunteer, as the Investigational Medicinal Products (IMP) investigated in the trial, are administered simultaneously and only once at Visit 2 (Day 0). At the following visits (Visit 3, day 1 to Visit 4, day 28), only follow-up procedures in relation to the immune response and safety assessments are performed. The procedures of the follow-up visits will not expose a trial volunteer to any risk.

The trial can be terminated at any time if the sponsor or the principal investigator concludes that the trial poses an unacceptable risk to the volunteers.

If, for any reason, a volunteer wishes to discontinue her/his participation in the trial, or if, according to principal investigator's judgement, she/he must be withdrawn from the trial, the date and reason (if possible) for withdrawal must be recorded in the CRF.

8.6.2 Permanent contraindications

Permanent contraindications are listed as exclusion criteria in section 8.6.

8.6.3 Temporary contraindications

It will be the responsibility of the investigator to judge from a medical evaluation if a volunteer's condition on the day of the injections should lead to the postponement of the visit.

FEVER (≥ 38.3 ºC) on the day of the injections must lead to 2 days postponement of the injections.

9 Investigational medicinal products

The investigational medicinal product C-Tb, is manufactured at SSI in Denmark according to Good Manufacturing Practice (GMP) standards.

The w:w ratio of rdESAT-6 and rCFP-10 is 1:1 in the test solution. A dose of 0.1 µg C-Tb contains 0.05 µg rdESAT-6 and 0.05 µg rCFP-10 per 0.1 mL.

9.1 Composition

Investigational product: C-Tb

rdESAT-6..0.5 µg
rCFP-10..0.5 µg
Disodium hydrogen phosphate dihydrate.................................1.4 mg
Potassium dihydrogen phosphate..0.2 mg
Potassium chloride...0.2 mg
Sodium chloride...8.0 mg
Polysorbate 20...0.1 µL
Phenol..0.5 %
Water for injection..up to 1 mL

Comparative product: 2 T.U. Tuberculin PPD RT 23 SSI:

Tuberculin .... 0.4 µg
Disodium hydrogen phosphate dihydrate .... 7.6 mg
Potassium dihydrogen phosphate.. 1.5 mg
Sodium chloride .... 4.8 mg
Potassium hydroxychinoline sulphate..................................... 100 µg
Polysorbate 80.. 50.0 µg
Water for injections.. up to 1 ml

9.2 Treatments administered

The 2 T.U. Tuberculin PPD RT 23 SSI and the C-Tb agents look identical and both appear as clear, colourless solutions.

The test solutions are filled into 3 mL glass vials with perforable rubber stoppers and aluminium caps. Each vial is intended for administration of 0.1 mL test product to one volunteer.

The C-Tb agent is formulated as a 0.1 µg C-Tb / 0.1 mL solution for injection.

C-Tb is dissolved in phosphate buffered saline containing 0.01% Polysorbate 20 and with a pH adjusted between 6.9 and 7.9, and preserved with 0.5% phenol in accordance with the Ph. Eur. Test for the efficacy of antimicrobial preservation.

Each volunteer will in the one arm receive one injection of C-Tb (0.1 µg/0.1 mL) and in the other arm receive one injection of the reference agent 2 T.U. Tuberculin PPD RT 23 SSI according to a randomisation code.

9.2.1 Doses and administration

The C-Tb and 2 T.U. Tuberculin PPD RT 23 SSI skin test agents are administered by the Mantoux technique by staff with training and experience in the Mantoux testing technique.

The subject number given to a volunteer eligible for inclusion must match the subject number on the labels of the cardboard box containing the investigational products.

It will remain unknown for the volunteer, the staff at the investigational site and the trial staff at the sponsor’s site (SSI) in which arm the C-Tb skin test agent and the 2 T.U. Tuberculin PPD RT 23 SSI skin test agent are injected, until the randomisation code will be revealed at the time of reaching ‘clean file’ for the whole trial.

Administration of the C-Tb and 2 T.U. Tuberculin PPD RT 23 SSI skin test agents:

- The test products should preferably be taken out of the refrigerator half an hour before each injection is performed
- Before the administration the “Use before” date must be checked

- The test agents may only be administered if they appear as clear and colourless solutions

- Disinfection of the injection sites before the injections is not necessary. If the injection sites are disinfected anyway, they must be completely dry before injection of the test products

- The two vials in the package are clearly marked with the arm they should be applied to. For standardisation reasons the vial for RIGHT arm should always be used first

- Use a sterile 1.0 mL disposable syringe fitted with a short-bevelled needle 26 gauge for the administration of 0.1 mL of the test solutions by the Mantoux technique

- Stretch the skin slightly and hold the needle almost parallel to the skin with the bevelled side upwards. The needle should be inserted about 2 mm into the flexor surface of the RIGHT/LEFT forearm at the junction of the upper third with the lower two-thirds. The needle should be visible through the epidermis before SLOWLY inserting 0.1 mL of the test agent intradermally. The appearance of a small papule of 8-10 mm in diameter after the injection indicates correct injection technique. The papule disappears after approximately 10 minutes

If one or both of the injections fail, - do not result in a papule (as described above), this must be recorded in the CRF together with the reason. A failed injection must under no circumstances be repeated in the same volunteer. In case one of the injections fails in a given volunteer, he/she should (if possible) for safety concerns stay in the trial for the follow-up visits. The volunteer should in this case be replaced by a new volunteer who will be given the next screening number available.

The syringe and the needle must be discarded as a single unit after injection in a labelled, puncture-proof container. The vial must be kept together with the cardboard box until drug accountability documents have been completed and approved by the monitor. This will normally take place at the termination monitoring visit.

9.2.2 Packaging and labelling

The packaging and labelling is performed at SSI according to Good Manufacturing Practice (GMP) standards. The C-Tb and the 2 T.U. Tuberculin PPD RT 23 SSI skin test agents cannot visually be distinguished from each other and the solutions look identical when drawn up in syringes as part of the test administration procedure. The labels are labelled with both Tuberculin PPD RT 23 SSI and C-Tb (0.1 µg / 0.1 ml). The inner label states in which arm the product should be injected without revealing the contents of the specific vial.

9.2.3 Transport of trial vaccines

The C-Tb skin test agent and the 2 T.U. Tuberculin PPD RT 23 SSI will be packed at Statens Serum Institut and sent by courier to the trial sites. Temperature loggers will be packed with the trial products prior to shipping for monitoring of the temperature conditions during the transport from Statens Serum Institut to the storage facility at the trial site. The temperature loggers will

be collected by trial staff upon arrival to the storage facility, and sent back to SSI according to detailed instructions given by Statens Serum Institut.

The IMP may not be dispensed by the site responsible investigator before the clinical trial manager at SSI confirms that the transport was carried out according to the temperature requirements.

9.2.4 Storage information

The stability of C-Tb is followed during the trial and is 24 months if stored in a refrigerator at + 2ºC to + 8ºC. The declared shelf-life of 2 T.U. Tuberculin PPD RT 23 SSI is 36 months if stored in a refrigerator at + 2ºC to + 8ºC. For blinding purposes the date of expiry is the same for 2 T.U. Tuberculin as for C-Tb in this clinical trial. The earliest of the expiry dates is selected.

The vials should be kept in the cardboard boxes until administered. The "Use before" date is printed on the "outer" labels on the cardboard boxes.

The IMPs are stored in a refrigerator at Surrey Clinical Research Centre, University of Surrey. The refrigerator is equipped with a temperature surveillance system and alarm systems.

In case the refrigerator, where the IMPs are stored, breaks down, the test items should be moved to another refrigerator as soon as possible. The reason and time of when the IMPs are moved to another location must be registered in the temperature log of the IMP.

In case of significant deviations in storage conditions, as judged by the principal investigator, the respective agents may not be used. The SSI clinical trial monitor should seek information from the clinical trial manager at SSI, in order to decide if the agents should be destroyed or may be used in the trial.

Trial staff at the trial site will be responsible for the monitoring of storage conditions. The site responsible investigator at the University of Surrey is responsible for the dispensing of the trial products. The dispensed IMP will be recorded in a dispensing log.

9.2.5 Randomisation procedure

The study comprises 1 dose of C-Tb (0.1µg / 0.1 mL) and 1 dose of 2 T.U. Tuberculin PPD RT 23 SSI.

Each volunteer receives both skin test agents in a split body design, the C-Tb is injected in one arm and the 2 T.U. Tuberculin PPD RT 23 SSI injected in the other arm.

To allow for an unbiased evaluation of the skin test reactions and any adverse events the two skin tests (C-Tb and Tuberculin PPD RT 23 SSI) are randomly allocated to the right and left arm in a double blind fashion.

The randomisation is performed by a statistician at the Biostatistics Unit at SSI, who will not be involved in the statistical analysis of the data from the trial.

300 test kits with 2 vials each (one with C-Tb and one with 2 T.U. Tuberculin PPD RT 23 SSI) are prepared. By means of a random permutation in a statistical analysis program (SAS), the subject numbers are randomly allocated to the two treatments in blocks of 10 volunteers, 5 of them receiving the C-Tb formulation in the right arm and 5 receiving the 2 T.U. Tuberculin PPD RT 23 SSI formulation in the right arm. The SAS program also prepares packing lists for the test kits, individual labels for the vials and the cardboard boxes, as well as emergency envelopes for each volunteer, making it possible, if necessary, to reveal the actual allocation of the C-Tb skin test agent without unblinding the whole trial.

Screening Numbers:

All volunteers screened will be allocated the lowest available screening number at visit 1. The screening numbers available are S500-S999.

Subject Numbers:

At the investigational site, the physical randomisation procedure consists in allocation of the lowest available subject number to a new eligible volunteer at Visit 2, and administration of the skin tests with the corresponding number. The labels on the vials specify whether the test agent should be injected in the right or the left arm.

300 test kits, each with 1 vial of 0.1 µg/0.1 ml C-Tb and 1 vial of 2 T.U. Tuberculin PPD RT 23 SSI will be labelled with subject numbers 001-300.

The extra subject numbers are needed in case an injection is unsuccessful and a volunteer will need to be replaced.

9.2.6 Blinding and unblinding

This study is double blind and therefore neither the trial staff, sponsor, nor the volunteer will know which arm is injected with the C-Tb skin test and which arm is injected with 2 T.U. Tuberculin PPD RT 23 SSI. The identity of the injections given to a particular volunteer's right or left arm (C-Tb or 2 T.U. Tuberculin PPD RT 23 SSI) can be revealed by breaking a sealed emergency code envelope. There will be one set of emergency envelopes at the trial site kept under the responsibility of the site responsible investigator, and there will be one set in the Department of Regulatory & Medical Affairs, SSI, kept under the responsibility of the Quality Person for Pharmacovigilance (QPPV), SSI.

In the event of a medical emergency, the code may be broken by the investigator ONLY if the information given in the emergency code envelope is of relevance for the further treatment of the volunteer. In this case, the code must be kept STRICTLY CONFIDENTIAL, and must ONLY be revealed to investigational site staff directly involved in the medical emergency on a need to know basis.

The Department of Regulatory & Medical Affairs, SSI may break the code ONLY if necessary to comply with serious adverse reaction reporting requirements of the competent authority (CA)

or ethics committee (EC) (see section 11.4 and 11.5). In this case, the code must be kept STRICTLY CONFIDENTIAL and must under no circumstances be revealed to any person not directly involved in the reporting, including the principal investigator, the clinical trial manager, the trial statistician or any person working with laboratory or statistical analysis of the trial data and interpretation of the trial results.

If the code is broken, the time, date, reason for breaking it, and, finally, the signature of the person breaking it, must be recorded on the emergency code envelope. The broken emergency code envelope must be kept together with the volunteer's case record form if broken at the investigational site, or according to internal procedures at SSI if broken in Department of Regulatory & Medical Affairs, SSI.

The clinical trial manager at SSI must be informed within 72 hours after breaking the code/envelope (without being informed about the actual treatment code).

9.2.7 Treatment compliance

As the test products are injected by study staff during the study visits under controlled conditions, treatment compliance procedures are not relevant in the present clinical trial.

9.2.8 Drug accountability

All used and unused vials must be kept at the trial site until the SSI monitor has performed the final check of drug accountability as part of the termination monitoring visit.

It is the responsibility of the principal investigator to make sure that all vials are accounted for during the clinical trial and it is the responsibility of the SSI monitor to check this at the routine monitoring visits.

Upon completion of the termination monitoring visit, the SSI monitor will give permission for the vials to be destructed at the trial sites or that the vials are returned to SSI for destruction. All dispensed IMPs will be recorded in a dispensing log.

9.2.9 Precautions and overdosing

According to the Summary of Product Characteristics for Tuberculin PPD RT 23 SSI [35] approved in Denmark and Germany anaphylactic reactions have not been seen in relation to tuberculin testing.

In the previous trials with C-Tb or rdESAT-6 no anaphylactic reactions have been seen.

Necessary treatment for an anaphylactic reaction must however always be available at the trial site.

A vasovagal reaction to the Mantoux injection can occur. The risk will be minimised by ensuring that the volunteer is seated on a reclining chair prior to administration of the skin test agents. Should a vasovagal reaction occur the volunteer can be positioned lying down and the pulse and blood pressure being recorded until recovery. Should this event occur it will be

documented in the CRF. This will be further clarified and mitigated in the Risk Management Plan.

9.2.10 Concomitant medication

Concomitant medication considered necessary for the volunteer during the course of the trial should be recorded in the concomitant medication pages of the CRF of the volunteer.

Decisions on withdrawal will be at the discretion of the investigator. See section 8.6 for medications and live vaccines that are listed under exclusion criteria.

9.3 Investigational events

Visit No.	1[1]	2	3	4	4x
Trial day	-28 days-0	0	2-3	28	Term. Visit [2]
Information of volunteer	×				
Signing of Consent Form<br>- allocate Screening No.	×				
In-/exclusion criteria	×	×			
Demography	×				
Medical history	×				
General medical examination	×			×	×
Blood samples (central lab.)[3]	×			×	×
Blood sample for QuantiFERON®-TB Gold In-Tube assay[4]	×			×[5]	×[5]
Urine test for drug screening (i.e., amphetamines, barbiturates, benzodiazepines, cannabinoids, cocaine, or opiates)	×	×			
Urine pregnancy test (female volunteers)		×			
Alcohol breath test	×	×			
Inclusion/randomisation<br>- allocate Subject No.		×			
Skin testing with C-Tb 0.1µg)		×			
Skin testing with 2 T.U. Tuberculin PPD RT 23 SSI		×			
Hand out diaries		×			
Collect diaries				×	×
Concomitant medication	×	×	×	×	×
Adverse events reporting		×	×	×	×
Reaction measurement and digital photo of reaction			×	×	×
Trial completion				×	×

1) The screening visit (Visit No. 1) takes place up to 28 days before the Inclusion visit (Visit No. 2)
2) A termination visit will be performed if a volunteer is withdrawn prior to visit 4 (day 28)
3) Blood samples for: RBC, haemoglobin, haematocrit, differential WBC, platelets, AST, ALT, alkaline phosphatase, albumin, bilirubin, creatinine, glucose, potassium, sodium and HIV (HIV only at inclusion visit)
4) Blood sample for measuring *in-vitro* IFN-γ response with the QuantiFERON®-TB Gold In-Tube assay.
In case the result of the QFT assay is indeterminate the volunteer will be offered an extra screening visit where a blood test can be drawn for a new QFT assay. The blood test must be drawn in due time to receive the result before skin testing at visit 2. If the 2nd QFT result is indeterminate the volunteer will be excluded from further participation in the trial.
5) Subjects with a positive QuantiFERON®-TB Gold In -Tube test result will be asked to come for a final check 6 months after the last visit in the trial (Visit 4)

9.3.1 Specificity assessment

The specificity of the C-Tb skin test is defined as the probability of a negative C-Tb test result in a population with no exposure to TB (= Prob {C-Tb^- | TB^-}). The specificity depends on the value chosen as the cut-off point for a positive C-Tb result. By definition the cut-off value is the smallest induration diameter (in mm) giving a C-Tb^+ result, i.e., C-Tb^+ = {Induration $\geq$ cut-off}. With these definitions the specificity of the C-Tb skin test is Prob {C-Tb^- | TB^-} = Prob {Induration < cut-off} and is, in the present study population, estimated by the relative frequency of patients with induration < cut-off, corresponding to 1 – F^-(cut-off - 1), where F^- is the distribution function of the induration responses.

A low cut-off value implies a high sensitivity but a low specificity; and a high cut-off value implies a low sensitivity but a high specificity.

Together with a concurrent C-Tb study in TB patients, TESEC-04, the data from the present study will contribute to define an optimal cut-off value, balancing sensitivity and specificity via a ROC curve.

9.3.2 Safety assessments

Local and systemic adverse events (AEs) will be assessed through medical assessments the first hour after the skin test injections and at the 2 follow up visits performed following the injections of the skin test agents on day 2-3 and day 28, respectively. All volunteers will be given a diary on day 2 or 3 where all AEs (local and systemic) experienced during the 28 days should be recorded. Medical examinations and safety laboratory tests will be performed at Visits 1 and 4.

All AEs and out of range laboratory parameters will be assessed by the investigator, and any serious AEs will be reported, as specified in Section 11.3. A detailed description of any local or systemic adverse events will be made in the CRF.

In case of injection site AEs, additional images, if applicable, will be made of the injection site(s) at the time of occurrence. The course of any adverse events will be documented by sequential descriptions and, if applicable, by making digital images until the events have completely disappeared.

The effect of the C-Tb skin test agent applied to the mother of an unborn child has not been investigated as C-Tb only induces a local celluar reaction in MTb infected individuals in the form of a delayed-type hypersensitivity skin reaction. Hence, it is considered acceptable not to perform development and reproductive studies with C-Tb. However, a repeated dose toxicity study has been performed in rats. Histopathological examination of tissues and organs including ovaries and testes did not reveal any changes related to the test agent.

It is routine practice to exclude pregnant women, women who plan to become pregnant or nursing mothers from the early trials until more safety information have been obtained. In the TESEC-03 trial women who could become pregnant during the study will be tested for

pregnancy before the injections of the skin tests and will be recommended to use an effective contraceptive during the course of the study. Any woman who finds or suspects that she has become pregnant while participating in the study will be requested to inform the research physician immediately AND will be followed up where possible to record the outcome.

The two antigens in C-Tb are also present in Tuberculin PPD RT 23 SSI. PPD has been used for 100 years in millions of people world-wide and has been found to be safe during pregnancy and lactation as stated in the Tuberculin PPD RT 23 SSIs currently approved SmPC for Denmark and Germany. C-Tb and Tuberculin PPD RT 23 SSI have comparable safety profiles and women of childbearing potential may be included in future phase III clinical trials with C-Tb without prior pregnancy testing.

For safety reasons the injections will be postponed 2 days if a volunteer on the day of injections has an oral temperature ≥ 38.3 °C, and/or if the volunteer is in treatment with analgesics/ antipyretics. Furthermore, there will be at least 30 minutes between the injections to different volunteers. All volunteers will be observed for one hour after the injections by experienced study staff at the trial site. Immediate AEs, if any will be recorded in the CRF.

9.3.3 Collection and handling of blood samples

Blood samples collected by venepuncture will be drawn by experienced clinical personnel according to standard procedures.

HIV test:

5 mL of clotted blood will be collected at the screening visit.

Blood samples for laboratory safety tests:

4 mL blood in EDTA tubes (PURPLE TOP) for blood count and 3.5 mL blood in coagulation tubes (YELLOW TOP) for chemistry will be collected at each sampling occasion for measuring RBC, haemoglobin, haematocrit, differential WBC, platelets, AST, ALT, alkaline phosphatase, albumin, bilirubin, creatinine, glucose, potassium and sodium.

Safety blood tests taken at Surrey Clinical Research Centre, Surrey will be analysed at the accredited hospital laboratory, The Royal Surrey County Hospital Guildford. The blood samples will be identified using the study number, subject initials and date of birth (DOB) to secure the confidentiality of the volunteers.

Blood samples QuantiFERON®-TB Gold In-Tube assay:

Standard test tubes provided by the manufacturer are used, handled, stored, and analysed according to the manufacturer’s instructions. Blood samples are incubated on the day they are taken. 3 mL blood is drawn into the tubes for the QFT assay provided by the manufacturer for measuring *in-vitro* IFN-γ response.

The accredited laboratory TDL (The Doctor's Laboratory, 60 Whitfield Street, London, W1T 4EU, UK) will be used to analyse the QFT assay. The date, study number and DOB will be used as identifiers to secure the confidentiality of the volunteers.

In case the result of the QFT assay is indeterminate the volunteer will be offered an extra screening visit where a blood test can be drawn for a new QFT assay. The blood test must be drawn in due time to receive the result before skin testing at visit 2. If the 2nd QFT result is indeterminate the volunteer will be excluded from further participation in the trial.

There is a small risk that this blood test can become positive during the trial due to the injected C-Tb skin test agent. Volunteers with a positive QFN test taken at the last visit (Visit 4) will therefore be offered an additional visit 6 months after the last visit in the trial to be re-tested with the QFN assay.

Each laboratory parameter outside of normal range judged by investigator to be non-clinically significant should be marked in the print outs with "NCS" together with the date and initials of the investigator.

9.3.4 Time schedule

The documents to the Ethics Committee (EC) and to the Competent Authority (MHRA) are planned to be submitted in November 2010.

The first volunteer's first visit (FPFV) is planned to take place in Q1 2011.

The last volunteer's last visit (LPLV) is planned to take place in Q4 2011.

10 Ethical aspects

TB continues to be a major cause of morbidity and mortality throughout the World, being one of the most important fatal infections of human beings. WHO estimates that 9.4 million new cases of tuberculosis occurred in 2008 with an estimated 1.3 million deaths in HIV negative people and an additional 500 000 deaths among HIV-positive people [2, 3, 4].

To control the disease, fast and accurate diagnosis is very important. The new diagnostic tool to be investigated in this phase II trial is a traditional tuberculin like skin test agent. However unlike the classical tuberculin PPD which contain a complex mixture of various antigens, C-Tb contains a mixture of two purified antigens, rdESAT-6 and rCFP-10, making C-Tb more specific than PPD.

C-Tb has previously been tested in healthy adult volunteers and adults recently diagnosed with active TB. Please refer to the Investigator's Brochure for further information.

Participation in the trial is voluntary, and the volunteer may choose to leave the trial at any time without any specific reason and still be entitled to continuing medical care.

Before inclusion in the trial, all volunteers will be informed (verbally and in writing) that there is a risk of unexpected adverse reactions, and that this risk has to be taken into account when considering participation. Detailed information about all trial procedures and the inconveniences these may pose on the volunteer will be explained as well. Informed consent will be obtained from all volunteers.

There will be at least 30 minutes between the injections of C-Tb and 2 T.U. Tuberculin PPD RT 23 SSI to different volunteers and all volunteers will be observed for one hour after the injections by experienced study staff.

Volunteers should not expect any benefits from participation, except, for the benefit of the intensive general health status investigations that will be performed during the trial.

The total amount of blood needed from each volunteer will be approximately 26 mL. Blood samples will be coded before they are sent for laboratory analyses, and only the principal investigator and the study staff will have access to information that may link laboratory results with personal identification.

Based on the non-clinical pharmacology and toxicity studies, two phase I clinical trials with rdESAT-6 (alone) and two phase I trials with C-Tb, it is expected that the administration of C-Tb by the Mantoux injection technique in the dose 0.1 µg will only expose the volunteers to minimal risks, such as local reversible adverse reactions at the injection sites. C-Tb is not likely to cause any systemic adverse reactions.

2 T.U. Tuberculin PPD RT 23 SSI, which is co-administered to the trial volunteers, is an established TB diagnostic test, which has been in use for more than 50 years on a world-wide basis. Adverse reactions described in the Summary of Product Charachteristics (SmPC) for 2 T.U. Tuberculin PPD RT 23 SSI approved in Denmark and Germany are:

- Hypersensitivity to tuberculin causing vesicles and superficial skin necrosis at the injection site
- Pain, itching and discomfort at the injection site
- Regional lymph node swelling and fever

In conclusion, it is anticipated that there is a reasonable balance between the risks and the inconveniences to which the volunteers will be exposed and the benefits they may obtain as a result of the increased medical attention which is part of the clinical trial, i.e. medical examinations and laboratory investigations.

10.1 Payment of volunteers

In recognition of the time involved in participating in the study, volunteers will be compensated with £50 for each visit to the hospital, which equals a total of £200 for the completion of all

scheduled visits. Compensation for travel expenses will be paid after the completion of each visit.

Subjects with a positive QuantiFERON®-TB Gold In -Tube test result at the final visit 4 (day 28) will be asked to come for a final check 6 months after the last visit in the trial (Visit 4) and will be compensated with additional £40.

10.2 Data Safety Monitoring Board

The clinical trial is expected to expose the trial volunteers to minimal risks only. The principal investigator and the site responsible investigator take the responsibility for the safety of the volunteers in the daily clinical practice. The investigational product C-Tb will be administered at a fixed dose and only once during the trial to each volunteer. Due to the above design no Data Safety Monitoring Board has been assessed necessary for this trial.

11 Adverse events

This section reviews the procedures for recording and reporting of AEs in the trial. Relevant definitions and terms are listed. Furthermore, the procedures for immediate reporting of serious AEs to SSI and for expedited reporting to the competent authorities and to the local ethics committee are described.

11.1 Definitions and terms

All definitions in the following are in accordance with the ICH E2A guideline [32].

Adverse Event (AE)
Any untoward medical occurrence in a volunteer or a volunteer participating in a clinical investigation and receiving a pharmaceutical product, which does not necessarily have a causal relationship with this product.

Adverse Reaction/Adverse Drug Reaction (AR/ADR)
Any untoward and unintended response to an investigational product related to any dose administered. The terms 'Adverse Reaction' and 'Adverse Drug Reaction' are the same thing (in practice), and imply that there is a suspected relationship between the event and the trial product. In practice this means that there is evidence or arguments that suggest a causal relationship, i.e. a relationship cannot be ruled out.
For further details, see ICH Topic 2A and EU directive 2001/20/EC.

Seriousness criteria
A serious AE or AR is any untoward medical occurrence that at any dose:

- results in death
- is life-threatening
- requires hospitalisation or prolongation of hospitalisation

- results in persistent or significant disability/incapacity
- is an important medical condition

NOTE: The term 'life-threatening' in the above definition refers to an event during which the volunteer was at risk of death at the time of the event, it does not refer to an event which hypothetically could have caused death, had it been more severe.

For further details, see ICH Topic 2A and EU directive 2001/20/EC.

Unexpected adverse reaction
An AR, the nature or severity of which is **not** consistent with the applicable product information (e.g., the Investigator's Brochure).

If a serious suspected AR is unexpected it is a 'Suspected Unexpected Serious Adverse Reaction' (SUSAR).
For further details, see ICH Topic 2A and EU directive 2001/20/EC.

11.2 Standard reporting of adverse events

The investigator is responsible for the recording of all reported AEs in the Adverse Event Forms of the CRFs during the visits. The investigator must use the following terms:

The **causal relationship** between an AE and the trial tests is assessed using the following terms:

- Not related
- Possible
- Probable
- Certain

The **intensity** of an AE is assessed using the following terms:

- Mild (i.e. easily tolerated)
- Moderate (i.e. sufficient to interfere with daily activities)
- Severe (i.e. sufficient to prevent normal activity)

Furthermore, for AEs that are included in the FDA Guidance for Industry, September 2007 [37], the intensity will as far as possible be rated according to the more specific rating system suggested in this guidance document.

If an AE is assessed as Potentially life threatening = grade 4, according to [37], this implies that the AE is serious and that expedited reporting to SSI by use of the CIOMS form is required, as described in section 11.3 of this protocol.

The **outcome** of an AE is assessed using the following terms:

- Fatal
- Not yet recovered
- Recovered with sequelae
- Recovered without sequelae
- Unknown

NOTE: If an AE is still ongoing at the last visit, it must be followed by the investigator until it has resolved or stabilised.

The **seriousness** of an AE is assessed by answering the following questions:

- Did the event result in death?
- Has the event been or is the event life-threatening?
- Has the event required inpatient hospitalisation or prolonging of hospitalisation?
- Has the event resulted in significant or persistent disability or incapacity?
- Is the event medically important

To ensure no confusion or misunderstanding of the difference between the terms "serious" and "severe," which are not synonymous, the following note of clarification is provided:

The term "severe" is often used to describe the intensity (severity) of a specific event (as in mild, moderate, or severe myocardial infarction); the event itself, however, may be of relatively minor medical significance (such as severe headache). This is not the same as "serious," which is based on patient/event outcome or action criteria usually associated with events that pose a threat to a volunteer's life or functioning. Seriousness (not severity) serves as a guide for defining regulatory reporting obligations.

11.3 Investigator's immediate reporting of serious adverse events to SSI

If a serious AE/AR (see definitions in section 11.1) occurs, the investigator is responsible for completing a CIOMS from (see template in Appendix 3), in addition to the adverse event form in the CRF [38].

The completed CIOMS form must be sent to the Department of Regulatory & Medical Affairs at SSI as soon as possible and at the latest 72 hours after his/her first knowledge of the serious AE/AR.

The completed CIOMS form must be sent ***by fax*** to the following SSI address:

Statens Serum Institut
Department of Regulatory & Medical Affairs
Fax: +45 32 68 39 73
Att.: Medical Affairs

The initial report should be followed by follow-up reports (using the same form) if additional important information becomes available.

11.4 SSI's expedited reporting of SUSARs to CA and EC

The time from SSI's first knowledge of a SUSAR (see definitions in section 11.1 and 11.4) until expedited reporting to CA/EC is as follows [39, 40]:

- For SUSARs resulting in death or life-threatening conditions, notification no later than seven (7) calendar days after SSI's first knowledge of the AR and reporting after an additional eight (8) calendar days

- For other SUSARs reporting no later than fifteen (15) calendar days after SSI's first knowledge of the AR (notification prior to this is not required)

MHRA only accepts electronic SUSAR reporting and the Department of Regulatory & Medical Affairs, SSI will be responsible for reporting by email of **unblinded** SUSAR reports according to the above timelines to MHRA.

Reports scanned as PDF documents can be e-mailed to pharmacovigilance@mhra.gsi.gov.uk, clearly stating that the attachment is a 'UK clinical trial SUSAR'.

The Department of Regulatory & Medical Affairs, SSI will in addition be responsible for the reporting by ordinary mail of **unblinded** SUSAR reports according to the above timelines to the EC

A NRES Safety Report Form enclosing the CIOMS forms should be used when reporting SUSARs to the EC [40].

Procedures for unblinding are described in section 9.2.6.

The Department of Regulatory & Medical Affairs, SSI will be responsible for distributing **blinded** copies of SUSAR reports according to the above timelines to the following parties:

- the principal investigator

- site responsible investigator(s)

- the clinical trial manager at SSI

The **blinded** SUSAR reports will be filed in the trial master file by SSI and in the investigator's file by the investigator/monitor.

11.5 Annual reporting of suspected serious adverse reactions to CA and EC

SSI is responsible for submitting annual safety update reports on **unblinded** suspected serious adverse reactions, including all SUSARs to the EC and the CA (MHRA) as electronic documents on disc to the EC and MHRA.

The MHRA address to be used is:

Information Processing Unit, Area 6
Medicines and Healthcare products Regulatory Agency, MHRA
Market Towers
1 Nine Elms Lane
Vauxhall
London SW85NQ
Great Britain

Annual Safety Reports should be prepared at yearly intervals from the date of the CTA approval and be submitted within sixty (60) days. If the clinical trial is ended before the first yearly Annual Safety Report should have been prepared, a safety report shall be prepared within ninety (90) days of the end of the trial, together with the end of trial notification.

The Department of Regulatory & Medical Affairs, SSI will be responsible for distributing blinded copies of these annual safety reports to the principal investigator, site responsible investigator(s) and the clinical trial manager at SSI.

The blinded SUSAR reports will be filed in the trial master file by SSI and in the investigator's file by the investigator/monitor.

12 Data management and statistical analysis

12.1 General considerations

A milestone in the clinical development of C-Tb is to determine on optimal cut-off value for the skin test. The cut-off value is defined as the smallest induration diameter that will result in a positive C-Tb "diagnosis". That is, a person is considered C-Tb positive ($C\text{-}Tb^+$) if the induration is $\geq$ cut-off and C-Tb negative ($C\text{-}Tb^-$) if the induration is < cut-off.

If a TB patient (latent or active) is denoted as TB^+ and a TB-healthy person as TB^- the performance of a diagnostic test like C-Tb is quantified by its sensitivity, i.e., $Prob\{C\text{-}Tb^+ \mid TB^+\}$ and by its specificity, $Prob\{C\text{-}Tb^- \mid TB^-\}$.

A low cut-off value implies a high sensitivity and a low specificity; and a high cut-off value implies a low sensitivity and a high specificity.

Together with a concurrent C-Tb study in recently diagnosed TB patients (TESEC-04) the data from the present study will contribute to define an optimal cut-off value, balancing sensitivity and specificity.

If $F^{-}(x)$ (= Prob{induration ≤ x}) denotes the (theoretical) distribution function of the induration in a TB^{-} population the specificity of the C-Tb skin test is F^{-}(cut-off - 1).

If $F^{+}(x)$ (= Prob{induation ≤ x}) denotes the (theoretical) distribution function of the induration in a TB^{+} population the sensitivity of the C-Tb skin test is 1- F^{+}(cut-off - 1).

The main objective in the present trial is therefore to estimate the distribution function F^{-}.

The Biostatistics Unit at SSI will be responsible for the data management and the statistical analysis of the trial data.

12.2 Data management

Clinical data is collected in the CRFs. The subject diaries are considered supportive for the investigators when filling in the CRFs, i.e. only relevant information from the diaries will be transferred to the CRFs by the investigator. The diaries will not be sent to the sponsor’s site but will remain filed at the investigator’s site and filed with the investigator’s copy of the CRFs.

SSI will receive laboratory data as signed printouts from the investigator. The printouts are identified by subject number and date of blood sampling.

Data from the CRFs and from laboratory records will be entered into SAS data sets and checked for consistency and plausibility by custom-made SAS programs. All ambiguous or implausible data items will be resolved by data queries to the investigator. When these findings have been resolved the SAS data sets will be copied to CDs, an audit trail established for the SAS data sets, and printouts of the datasets are proofread against the CRFs and laboratory printouts for the SAS data sets.

12.3 Clean file procedures

After correction of any errors found in the proofreading process, the SAS data sets reach clean file status. The SAS data sets will then be copied to CDs. Two identical sets of CDs will be made. One CD will be archived in the trial master file. The other CD will be archived at the Biostatistics unit at SSI.

The statistical analysis reported in the integrated clinical trial report will be based on the clean file data.

12.4 Analysis populations

The safety analysis population consists of all subjects who have received at least one TST (C-Tb or PPD) in the trial.

As the focus in this trial is on estimation of $F^-(x)$ the efficacy population will be a pure "per protocol population" meaning that only those subjects who meet the inclusion criteria and have an induration measurement on day 2 or 3 after the injections are included in the estimation.

12.5 Statistical methods

The protocol allows reading of the induration to take place on day 2 or day 3. To investigate the influence of the time of reading the induration the correlations between time of reading (hours since injection) and induration diameters are assessed by Spearman's rank correlation coefficient.

The induration diameters after the PPD skin test are compared with those after C-Tb by means of Spearman's rank correlation, Wilcoxon's signed rank test and supplemented with scatter plots.

Similarly the inclusion QuantiFERON®-TB Gold values are correlated with the C-Tb indurations by means of Spearman's rank correlation, supplemented with scatter plots.

12.6 Sample size determinations

The focus of the study is the estimation of $F^-(x)$. Power calculations are therefore not relevant for the sample size calculations. The sample size is instead based on the precision of the estimate $\hat{F}^-(x)$ of $F^-(x)$.

From the binomial distribution the variance of $\hat{F}^-(x)$ is $\mathrm{Var}(\hat{F}^-(x)) = \hat{F}^-(x)(1-\hat{F}^-(x))/n$. An approximate 95% confidence interval for fixed x is therefore $\hat{F}^-(x) \pm 2\sqrt{\hat{F}^-(x)(1-\hat{F}^-(x))/n}$, where n is the number of subjects in the group.

The highest value of $\mathrm{Var}(\hat{F}^-(x))$ $(= 0.5/\sqrt{n})$ is obtained if $F(x) = 0.5$.

The table below shows the standard deviation $\mathrm{SD}(\hat{F}^-(x))$ as a function of $F^-(x)$ for n=100, 150 and 200.

	Number of subjects (n)		
$F^-(x)$	100	150	200
0.5	0.050	0.041	0.035
0.8	0.040	0.033	0.028
0.9	0.030	0.024	0.021

With 150 subjects in the trial $\hat{F}^-(x)$ will always be estimated with a standard deviation ≤ 0.041

12.7 Interim analysis

No interim statistical analyses are planed.

13 Good clinical practice considerations

The ICH guidelines, ICH Topic E 6 [31] and ICH Topic E 2A [32], the EC directives 2001/20/EC [33] and 2005/28/EC [34] of the European Parliament (including underlying guidance) were followed when writing this protocol.

13.1 Declaration of Helsinki

This clinical trial will be conducted in accordance with the principles of the Declaration of Helsinki (see Appendix 1).

13.2 Subject information and informed consent

Volunteers are informed about the trial both verbally and in writing. The information is provided by trained staff in an objective way, describing both advantages and disadvantages of participation in the trial. The volunteer will be given reasonable time (min 24 hours) to consider whether he/she wishes to participate.

The written information and the informed consent form [41] are approved by the relevant EC and CA before use.

13.3 Independent ethics committee submission and approval

An application file will be submitted for approval to the Ethics Committee [42, 43, 44], and the inclusion of volunteers will only start AFTER the trial has been approved by this instance.

The trial site, Clinical Research Centre, University of Surrey is responsible for submitting the trial to the EC.

13.4 Competent authority submission and approval

An application file will be submitted for approval to the regulatory authorities in England [39, 44]:

Medicines and Healthcare products Regulatory Agency, MHRA
Information Processing Unit, Area 6
Market Towers
1 Nine Elms Lane
London, SW8 5NQ
Great Britain

Only after approval by both the above mentioned EC and the MHRA the clinical trial will be initiated.

SSI is responsible for the submission to MHRA.

13.5 Subject data protection

The site responsible investigator is responsible for keeping a list (a screening log) of all trial volunteers. All volunteers who are subject to at least one trial intervention such as for example the extended medical examination at the screening visit should be listed in the screening log. For screening failures, the reason for exclusion should be given in the screening log.

The screening log must identify the volunteers by: screening number, subject number (i.e. only given to volunteers who are included/have received the C-Tb and the 2 T.U. Tuberculin PPD RT 23 SSI skin test agents), full name and last address known, sex, and date of birth. At the end of the clinical trial the screening log must be signed off by the site responsible investigator at the trial site for correctness. This is done as part of the termination monitoring visit.

In the CRF, only screening number, subject number and date of birth must be recorded. It must always be possible to trace a CRF to the screening log.

The volunteers must be informed about and agree to the following:

- After the end of the clinical trial (i.e., after the date of the last visit of the last subject), the principal investigator will store the screening log for at least 15 years.
- After the end of the clinical trial, the screening log must be accessible by EC or CA for at least 15 years if needed in connection with an inspection.

13.6 Investigator's responsibilities

By signing this protocol, the principal investigator and the site responsible investigator(s) take the overall responsibility for the conduct of the clinical trial in accordance with the protocol, good clinical practice (GCP) [31, 32, 33, 34] and any relevant national regulations, for the complete and accurate recording of all data, including all reported AEs in the CRFs, and for immediate reporting of serious AEs to SSI, according to the procedures described in section 11 of this protocol.

The delegation of responsibilities to sub-investigators/nurses/important staff members must be specified in writing in the log of staff, or a similar trial document according to instructions, see the following section.

13.7 Curricula vitae and log(s) of staff

Investigational site(s):
Before initiation of the trial, current dated and signed curricula vitae in English for all investigators and staff members with significant responsibilities in the trial must be collected.

The log of staff of ALL staff members at the investigational site must be completed with responsibilities (incl. start date and end date of responsibilities), signature and initials (written by her/him self) by the staff member.

The monitor must go through the log of staff with the responsible investigator, for completeness and correctness at the trial initiation visit. The principal investigator will document this by signing off the log of staff with signature, date and initials.

During the active phase of the trial, the monitor must regularly check that the log of staff remains complete and correct at all times and resolve any outstanding issues.

Sponsor's site(s):
At the sponsor's site current, dated and signed curricula vitae in English for the clinical trial manager and monitor(s) etc. involved in the trial must be available. Furthermore, ALL staff members involved in the trial must be listed in a log of staff.

For SSI staff members who may make recordings in the CRFs (according to SSI SOPs) or other trial documents, initials must be recorded in the log off staff.

13.8 Indemnity statement

Prior to the inclusion of the first volunteer in the clinical trial, SSI will present a signed Indemnity Statement to all investigators participating in the clinical trial (see Appendix 2).

13.9 Training

The principal investigator is overall responsible for, that all staff members at the trial site are adequately qualified before the initiation of the trial.

If relevant the clinical trial manager will be responsible for performing training sessions covering general GCP issues as: safety procedures, reporting of suspected serious adverse reactions including SUSARs, information of volunteers, recording in CRFs, handling of IMP, randomisation procedures, blinding and unblinding procedures etc. If training is needed in relation to the Mantoux testing technique etc., the site responsible investigators will perform these sessions. All performed sessions will be documented in the CVs of the investigators and the staff members performing the Mantoux testing etc.

Previous GCP qualifications and experience must be stated in the current dated and signed CVs of the study staff. For investigators and nurses (or other staff members with significant responsibilities in the trial) who have no previous documented training in GCP, GCP certificates will be issued by the clinical trial manager upon satisfactory participation in the above listed GCP training session(s).

13.10 Monitoring

The clinical trial monitor from SSI will make regular visits to the trial site. Together with the study staff at the site, the monitor will, for example, check the following:

- That the protocol is being followed
- That facilities and staffing remain acceptable

- That the CRFs are being correctly filled in
- That the CRFs are in accordance with source data
- That the clinical supplies (especially the IMP) can be accounted for
- That the IMP have been transported according to requirements and are stored properly at the investigational site
- That the Investigator's File is being kept in proper order

All visits to the investigational site are documented. Any query is discussed and resolved with the investigator or relevant study staff. The monitor will work according to a monitoring plan approved by the clinical trial manager and will follow SSI's GCP SOPs for GCP-monitoring.

13.11 Audit and inspection

The investigator must give access to personnel from SSI for the conduct of audits at the investigational site. Auditors and inspectors from CA, EC or other relevant authorities must at all times be allowed access to conduct inspections as well and must be given access to all trial related documents, including the Investigator's File and the volunteers' personal medical records, if applicable.

13.12 Definition and archiving of source data

Source data is defined as all information in original records and certified copies of original records necessary for the reconstruction and evaluation of the clinical trial. A document, which identifies the source data in this clinical trial, will be prepared and signed by responsible investigator before initiation of the trial. In the following examples of source data are listed:

- EC approval documents
- Approval from the MHRA
- Signed informed consent forms
- The screening log
- Investigator and nurse notes (i.e. for data either not recorded in the CRF or for data not recorded directly in the CRF)
- Medical records/journals
- Print outs of laboratory results
- The CRF (for data recorded directly in the CRF)
- Volunteer diaries

- IMP inventory and accountability logs
- IMP storage condition log(s)

13.13 Definition and archiving of essential documents

It is the responsibility of the principal investigator and SSI to maintain the essential documents as described in the ICH guidelines for at least 15 years after the termination of the trial. In the present clinical trial termination is defined as the last volunteer's last visit.

The completeness of the TMF and the IF are verified by the monitor at trial initiation, during the active phase of the trial and, finally, at the end of the trial as part of the trial termination. At the trial termination visit at the investigational site(s), the monitor goes through the archiving requirements with the responsible investigator and will assure that the archiving requirements are met.

14 Agreement and financial settlement

The agreement between the Investigational institution (the University of Surrey) and SSI must be signed prior to inclusion of the first volunteer in the clinical trial. The agreement must clearly state the rights and obligations of the parties concerned and include a detailed financial settlement.

Furthermore, a written agreement should be made between the principal investigator and the accredited laboratory TDL (The Doctor's Laboratory), regarding the laboratory analysis in the trial.

15 Insurance

SSI is the sponsor and manufacturer of the IMP to be administered in this clinical trial. SSI carries a product liability insurance programme including cover for clinical trials. The insurance programme covers world wide is currently placed with insurer CHARTIS through insurance brokers Bowring Marsh, Tower Place, London, EC3R 5BU, UK. The policy covers claims arising from injury/injuries caused by trial medication used in clinical trials sponsored by the company (SSI) if the trial medication has been used in accordance with the instructions given in the protocol. The insurance certificate is enclosed in Appendix 4.

16 Confidentiality and disclosure

All CRFs, information and results generated by SSI, as well as information on product development, patented or not, including patent applications and manufacturing processes not previously published, are considered confidential and shall remain the sole property of SSI.

An integrated statistical and clinical study report (CSR) will be prepared by SSI in co-operation with the principal investigator. A summary of the CSR will be submitted to the CA no later than one year after the end of the trial (the end is defined as the last volunteer's last visit).

No data from the clinical trial may be published, presented or communicated, except to CA or EC, prior to the release of the internal clinical trial report, unless approved by SSI in writing. All investigators agree not to discuss externally or publish any result from the trial without the possibility of SSI to give comments.

In the event of the publication of the trial results in a scientific journal the names of the authors and their order of appearance will be as specified in the agreement between the investigational institution/site (The University of Surrey) and SSI.

17 Changes to the protocol

The clinical trial procedures may be changed if the principal investigator and the clinical trial manager agree to the changes. If the changes are substantial, both the EC and CA must approve the changes before they can be implemented. All substantial changes must be documented by protocol amendments and if necessary rewritten full protocols.

18 References

1. Points to Consider on the Evaluation of Diagnostic Agents. Committee for Proprietary Medicinal Products (CPMP/EWP/1119/98)
2. World Health Organisation. Global Tuberculosis Control- epidemiology, strategy, financing. WHO Report 2009, (WHO/HTM/TB/2009.411)
3. Global tuberculosis control: a short update to the 2009 report (World Health Organisation. Global Tuberculosis Control- epidemiology, strategy, financing. WHO Report 2009, (WHO/HTM/TB/2009.411))
4. TB/HIV FACTS 2009 (http://www.who.int/tb/challenges/hiv/factsheet_hivtb_2009update.pdf)
5. www.undp.org/mdg/goal6.shtml
6. TB diagnosis - a challenge to science and policy, EAGLES European Action on Global Life Sciences. November 2008
7. Pai Madhukar, Kalantri Shriprakash, Dheda Keertan. New tools and emerging technologies for the diagnosis of tuberculosis: Part 1. Latent tuberculosis. Expert Rev. Mol. Diagn. 6(3), (2006). 2006 Future Drugs Ltd.
8. Snider DE. Tuberculin test. Am Rev Respir Dis 1982; 125: 108-18

9. Huebner RE, Schein MF, Bass JB. The tuberculin skin test. Clin Infect Dis 1993; 17: 968-75

10. Andersen P, Munck ME, Pollock JM, Doherty TM. Specific immune-based diagnosis of tuberculosis. Lancet 2000; 356: 1099-104

11. Lange, C; Pai, M; Drobniewski, F and Miglior, G. B. Interferon-γ release assays for the diagnosis of active tuberculosis: sensible or silly. Eur Respir J 2009: 1250-1253

12. Andersen P, Andersen AB, Sorensen AL, Nagai S. Recall of long-lived immunity to Mycobacterium tuberculosis infection in mice. J Immunol 1995; 154: 3359-72

13. Van Pinxteren A H, Laurens et al. Diagnosis of Tuberculosis Based on Two Specific Antigens ESAT-6 and CFP10.Clinical and Diagnostic Laboratory Immunology, Mar 2000, p. 155-160

14. Pai Madhukar, Riley Lee W, Colford John M Jr. Interferon-□ assays in the immunodiagnosis of tuberculosis: a systemic review. The Lancet, Infectious Diseases Vol 4 December 2004.

15. Ulrichs T, Munk ME, Mollenkopf H, et al. Differential T-cell responses to Mycobacterium tuberculosis ESAT-6 in tuberculosis patients and healthy donors. Eur J Immunol 1998; 28: 3949-58

16. Ravn P, Demissie A, Eguale T, et al. Human T-cell responses to the ESAT-6 antigen from Mycobacterium tuberculosis. J Infect Dis 1999; 179: 637-45

17. Johnson PD, Stuart RL, Grayson ML, Olden D, Clancy A, Ravn P, Andersen P, Britton WJ, Rothel JS. Tuberculin-purified protein derivative-, MPT-64-, and ESAT-6-stimulated gamma interferon responses in medical students before and after M. bovis BCG vaccination and in patients with tuberculosis. Clin Diagn Lab Immunol 1999; 6: 934-37

18. Lein AD, von Reyn CF, Ravn P, Horsburgh CR et al.Cellular immune responses to ESAT-6 discriminate between patients with pulmonary disease due to M.avium complex and those with pulmonary disease due to M. tuberculosis.Clin Diagn Lab Immunol 1999; 6: 606-609

19. Aggerbeck H, Madsen S.M. Safety of ESAT-6. Elsevier, Tuberculosis (2006) 86; 363-373

20. Renshaw P, Lightbody K.L, Veverka V et al. Structure and function of the complex formed by the tuberculosis virulence factors CFP-10 and ESAT-6. The EMBO Journal, 2005, 24, 2491-2498

21. Skjøt, R.L.V., Oettinger T, Rosenkrands I, Ravn P, Brock I, Jacobsen S, Andersen P. Comparative evaluation of low-molecular-mass T-cell antigens from Mycobacterium tuberculosis identifies members of the ESAT-6 family as immunodominant. Infect. Immun. 2000, 68: 214-220

22. Elhay MJ, Oettinger T, Andersen P. Delayed type hypersensitivity responses to ESAT-6 and MPT-64 from Mycobacterium tuberculosis in the guinea pig. Infect Immune 1998; 66: 3454-6.

23. Arend, Sandra M et al. Double blind randomized Phase 1 study comparing rdESAT-6 to tuberculin as skin test reagent in the diagnosis of tuberculosis infection. Tuberculosis (2007), doi: 10.1016/j.tube.2007.11.004.

24. Thierry-Carstensen B, Aggerbeck H and Jensen A.M. A phase I (first in man), double blind, "within subject" randomized and controlled, dose-adjusting clinical trial on the safety and the diagnostic potential of the rdESAT-6 skin test in the diagnosis of tuberculosis. Internal clinical study report date of issue 04-01-2008.

25. Lillebæk T, Bergstedt W, Tingskov PN, Thierry-Carstensen B, Aggerbeck H, Hoff ST, Weldingh K, Andernsen P, Søborg B, Thomen VO, Andersen AB. Risk of sensitization in healthy adults following repeated administration of rdESAT-6 skin test reagent by the Mantoux injection technique. Tuberculosis 2009;89:158-62

26. Tingskov P N, Thierry-Carstensen B, Aggerbeck, Jensen A. A phase I clinical trial to assess the risk of sensitisation in healthy adults following repeated administration of rdESAT-6 by the Mantoux injection technique. Internal clinical study report date of issue 03-04-2009.

27. Reece S.T., Stride N., Ovendale P., Reed S.G. et al. Skin test performed with highly purified Mycobacterium tuberculosis recombinant protein triggers tuberculin schock in infected guinea pigs. Infection and Immunity, June 2005, p. 3301-3306.

28. Internal SSI Report F1137

29. Andersen, Aase Bengaard et al. First-in-Man Open Clinical Trial of a Combined rdESAT-6 and rCFP-10 Tuberculosis Specific Skin Test Reagent. PLoS ONE June 2010, Volume 5, Issue 6, e11277

30. Tingskov, Pernille N, Aggerbeck H and Jensen A M. An open phase I clinical trial on the safety and the risk of sensitisation by escalating doses and repeated injections of the rdESAT-6 + rCFP-10 skin test reagent following intradermal administration to healthy adults. Internal clinical study report date of issue 18-06-2010

31. Note for guidance on good clinical practice. ICH Topic E 6. (CPMP/135/95)

32. Note for guidance on clinical safety data management: definitions and standards for expedited reporting ICH Topic E 2 A (CPMP/ICH/377/95)

33. Directive 2001/20/EC of the European Parliament and the council of 4 April 2001 on the approximation of the laws, regulations and administrative provision of the Member States relating to the implementation of good clinical practice in the conduct of clinical trials on medicinal products for human use. Official Journal L 121, 01/05/2001 P.0034 – 0044.

34. Directive 2005/28/EC of the European Parliament of 8 April 2005 laying down principles and detailed guidelines for good clinical practice as regards investigational medicinal products for human use, as well as the requirements for authorisation of the manufacturing or importation of such products.

35. Mutual Recognition Procedure (MRP) SmPC for Denmark and Germany approved on 08 May 2006

36. Immunisation against infectious disease 2006. The Green Book (updated 28 July 2010). Published by the The Stationery Office under license from the Department of Health, UK

37. Guidance for Industry: Toxicity grading scale for healthy adult and adolescent volunteers enrolled in preventive vaccine clinical trials, FDA September 2007

38. Detailed guidance on the collection, verification and presentation of adverse reaction reports arising from clinical trials on medicinal products for human use, Eudralex Vol. 10 Chapter II, rev2 2006.04

39. MHRA Homepage; Clinical trial authorisations, www.mhra.gov.uk

40. NRES Homepage; Safety reports for CTIMPs, www.nres.npsa.nhs.uk

41. Information sheets & Consent forms – Guidance for researchers and reviewers, Version 3.2 May 2007 (NRES)

42. Guidance for applicants to the National Research Ethics Service, 2007 (NRES)

43. Information sheet 5: Research Ethics, 2007, Wandsworth Teaching PCT

44. IRAS – Integrated Research Application System, www.myresearchproject.org.uk

WORLD MEDICAL ASSOCIATION DECLARATION OF HELSINKI

Ethical Principles for Medical Research Involving Human Subjects

Adopted by the 18th WMA General Assembly, Helsinki, Finland, June 1964, and amended by the:
29th WMA General Assembly, Tokyo, Japan, October 1975
35th WMA General Assembly, Venice, Italy, October 1983
41st WMA General Assembly, Hong Kong, September 1989
48th WMA General Assembly, Somerset West, Republic of South Africa, October 1996
52nd WMA General Assembly, Edinburgh, Scotland, October 2000
53th WMA General Assembly, Washington 2002 (Note of Clarification on paragraph 29 added)
55th WMA General Assembly, Tokyo 2004 (Note of Clarification on Paragraph 30 added)
59th WMA General Assembly, Seoul, October 2008

A. INTRODUCTION

1. The World Medical Association (WMA) has developed the Declaration of Helsinki as a statement of ethical principles for medical research involving human subjects**,** including research on identifiable human material and data.

   The Declaration is intended to be read as a whole and each of its constituent paragraphs should not be applied without consideration of all other relevant paragraphs.

2. Although the Declaration is addressed primarily to physicians, the WMA encourages other participants in medical research involving human subjects to adopt these principles.

3. It is the duty of the physician to promote and safeguard the health of patients, including those who are involved in medical research. The physician's knowledge and conscienceare dedicated to the fulfilment of this duty.

4. The Declaration of Geneva of the WMA binds the physician with the words, "The health of my patient will be my first consideration," and the International Code of Medical Ethics declares that, "A physician shall act in the patient's best interest when providing medical care."

5. Medical progress is based on research that ultimately must include studies involving human subjects. Populations that are underrepresented in medical research should be provided appropriate access to participation in research.

6. In medical research involving human subjects, the well-being of the individual research subject must take precedence over all other interests.

7. The primary purpose of medical research involving human subjects is to understand the causes, development and effects of diseases and improve preventive, diagnostic and therapeutic interventions (methods, procedures and treatments). Even the best current interventions must be evaluated continually through research for their safety, effectiveness, efficiency, accessibility and quality.

8. In medical practice and in medical research, most interventions involve risks and burdens.

9. Medical research is subject to ethical standards that promote respect for all human subjects and protect their health and rights. Some research populations are particularly vulnerable and need special protection. These include those who cannot give or refuse consent for themselves and those who may be vulnerable to coercion or undue influence.

10. Physicians should consider the ethical, legal and regulatory norms and standards for research involving human subjects in their own countries as well as applicable international norms and standards. No national or international ethical, legal or regulatory requirement should reduce or eliminate any of the protections for research subjects set forth in this Declaration.

B. PRINCIPLES FOR ALL MEDICAL RESEARCH

11. It is the duty of physicians who participate in medical research to protect the life, health, dignity, integrity, right to self-determination, privacy, and confidentiality of personal information of research subjects.

12. Medical research involving human subjects must conform to generally accepted scientific principles, be based on a thorough knowledge of the scientific literature, other relevant sources of information, and adequate laboratory and, as appropriate, animal experimentation. The welfare of animals used for research must be respected.

13. Appropriate caution must be exercised in the conduct of medical research that may harm the environment.

14. The design and performance of each research study involving human subjects must be clearly described in a research protocol. The protocol should contain a statement of the ethical considerations involved and should indicate how the principles in this Declaration have been addressed. The protocol should include information regarding funding, sponsors, institutional affiliations, other potential conflicts of interest, incentives for subjects and provisions for treating and/or compensating subjects who are harmed as a consequence of participation in the research study. The protocol should describe arrangements for post-study access by study subjects to interventions identified as beneficial in the study or access to other appropriate care or benefits.

15. The research protocol must be submitted for consideration, comment, guidance and approval to a research ethics committee before the study begins. This committee must be independent of the researcher, the sponsor and any other undue influence. It must take into consideration the laws and regulations of the country or countries in which the research is to be performed as well as applicable international norms and standards but these must not be allowed to reduce or eliminate any of the protections for research subjects set forth in this Declaration. The committee must have the right to monitor ongoing studies. The researcher must provide monitoring information to the committee, especially information about any serious adverse events. No change to the protocol may be made without consideration and approval by the committee.

16. Medical research involving human subjects must be conducted only by individuals with the appropriate scientific training and qualifications. Research on patients or healthy volunteers requires the supervision of a competent and appropriately qualified physician or other health care professional. The responsibility for the protection of research subjects must always rest with the physician or other health care professional and never the research subjects, even though they have given consent.

17. Medical research involving a disadvantaged or vulnerable population or community is only justified if the research is responsive to the health needs and priorities of this population or community and if there is a reasonable likelihood that this population or community stands to benefit from the results of the research.

18. Every medical research study involving human subjects must be preceded by careful assessment of predictable risks and burdens to the individuals and communities involved in the research in comparison with foreseeable benefits to them and to other individuals or communities affected by the condition under investigation.

19. Every clinical trial must be registered in a publicly accessible database before recruitment of the first subject.

20. Physicians may not participate in a research study involving human subjects unless they are confident that the risks involved have been adequately assessed and can be satisfactorily managed. Physicians must immediately stop a study when the risks are found to outweigh the potential benefits or when there is conclusive proof of positive and beneficial results.

21. Medical research involving human subjects may only be conducted if the importance of the objective outweighs the inherent risks and burdens to the research subjects.

22. Participation by competent individuals as subjects in medical research must be voluntary. Although it may be appropriate to consult family members or community leaders, no competent individual may be enrolled in a research study unless he or she freely agrees.

23. Every precaution must be taken to protect the privacy of research subjects and the confidentiality of their personal information and to minimize the impact of the study on their physical, mental and social integrity.

24. In medical research involving competent human subjects, each potential subject must be adequately informed of the aims, methods, sources of funding, any possible conflicts of interest, institutional affiliations of the researcher, the anticipated benefits and potential risks of the study and the discomfort it may entail, and any other relevant aspects of the study. The potential subject must be informed of the right to refuse to participate in the study or to withdraw consent to participate at any time without reprisal. Special attention should be given to the specific information needs of individual potential subjects as well as to the methods used to deliver the information. After ensuring that the potential subject has understood the information, the physician or another appropriately qualified individual must then seek the potential subject's freely-given informed consent, preferably in writing. If the consent cannot be expressed in writing, the non-written consent must be formally documented and witnessed.

25. For medical research using identifiable human material or data, physicians must normally seek consent for the collection, analysis, storage and/or reuse. There may be situations where consent would be impossible or impractical to obtain for such research or would pose a threat to the validity of the research. In such situations the research may be done only after consideration and approval of a research ethics committee.

26. When seeking informed consent for participation in a research study the physician should be particularly cautious if the potential subject is in a dependent relationship with the physician or may consent under duress. In such situations the informed consent should be sought by an appropriately qualified individual who is completely independent of this relationship.

27. For a potential research subject who is incompetent, the physician must seek informed consent from the legally authorized representative. These individuals must not be included in a research study that has no likelihood of benefit for them unless it is intended to promote the health of the population represented by the potential subject, the research cannot instead be performed with competent persons, and the research entails only minimal risk and minimal burden.

28. When a potential research subject who is deemed incompetent is able to give assent to decisions about participation in research, the physician must seek that assent in addition to the consent of the legally authorized representative. The potential subject's dissent should be respected.

29. Research involving subjects who are physically or mentally incapable of giving consent, for example, unconscious patients, may be done only if the physical or mental condition that prevents giving informed consent is a necessary characteristic of the research population. In such circumstances the physician should seek informed consent from the legally authorized representative. If no such representative is available and if the research cannot be delayed, the study may proceed without informed consent provided that the specific reasons for involving subjects with a condition that renders them unable to give informed consent have been stated in the research protocol and the study has been approved by a research ethics committee. Consent to remain in the research should be obtained as soon as possible from the subject or a legally authorized representative.

30. Authors, editors and publishers all have ethical obligations with regard to the publication of the results of research. Authors have a duty to make publicly available the results of their research on human subjects and are accountable for the completeness and accuracy of their reports. They should adhere to accepted guidelines for ethical reporting. Negative and inconclusive as well as positive results should be published or otherwise made publicly available. Sources of funding, institutional affiliations and conflicts of interest should be declared in the publication. Reports of research not in accordance with the principles of this Declaration should not be accepted for publication.

C. ADDITIONAL PRINCIPLES FOR MEDICAL RESEARCH COMBINED WITH MEDICAL CARE

31. The physician may combine medical research with medical care only to the extent that the research is justified by its potential preventive, diagnostic or therapeutic value and if the physician has good reason to believe that participation in the research study will not

adversely affect the health of the patients who serve as research subjects.

32. The benefits, risks, burdens and effectiveness of a new intervention must be tested against those of the best current proven intervention, except in the following circumstances:
    - The use of placebo, or no treatment, is acceptable in studies where no current proven intervention exists; or
    - Where for compelling and scientifically sound methodological reasons the use of placebo is necessary to determine the efficacy or safety of an intervention and the patients who receive placebo or no treatment will not be subject to any risk of serious or irreversible harm. Extreme care must be taken to avoid abuse of this option.

33. At the conclusion of the study, patients entered into the study are entitled to be informed about the outcome of the study and to share any benefits that result from it, for example, access to interventions identified as beneficial in the study or to other appropriate care or benefits.

34. The physician must fully inform the patient which aspects of the care are related to the research. The refusal of a patient to participate in a study or the patient's decision to withdraw from the study must never interfere with the patient-physician relationship.

35. In the treatment of a patient, where proven interventions do not exist or have been ineffective, the physician, after seeking expert advice, with informed consent from the patient or a legally authorized representative, may use an unproven intervention if in the physician's judgement it offers hope of saving life, re-establishing health or alleviating suffering. Where possible, this intervention should be made the object of research, designed to evaluate its safety and efficacy. In all cases, new information should be recorded and, where appropriate, made publicly available.

Indemnity Statement

Trial title: A phase IIa specificity trial of the diagnostic agent C-Tb, when given intradermally by the Mantoux technique to healthy volunteers previously vaccinated with BCG

Trial code: TESEC-03

Dear Professor David Lewis,

You have kindly agreed to consider undertaking the above-mentioned clinical trial as investigator, in accordance with the protocol for the trial TESEC-03.

In the event that any recruited patient in the trial should suffer any personal injury resulting from the clinical trial, SSI agrees to indemnify the institution where the clinical trial is being undertaken, Clinical Research Centre, University of Surrey, Guildford, United Kingdom, and, through Clinical Research Centre, University of Surrey, Guildford, United Kingdom, any of its employees or agents participating in the trial, against liability imposed by law, but not assumed voluntarily, and arising from the use of the test products in the trial, PROVIDED THAT:

1) SSI shall not indemnify against, nor have any obligation whatsoever as regards liability arising from or related to any error, omission, intentional wrongful act, or other negligence on the part of said institutions or persons, such as medical malpractice; and

2) Any such institution or person seeking indemnity

   a) has fully complied with the protocol for the trial, and

   b) has promptly notified SSI of any notice of any type of claim, or the likelihood of a claim, relating to the trial,

   c) as regards any claim, makes no statement, takes no action, nor makes any commitment affecting SSI's interests, without SSI's prior written consent, and further, provides all reasonable and necessary assistance to SSI in the defence of any claim, allowing SSI, at its cost and in its discretion, to take over the defence of any action and to have full control in handling the claim.

Please note that this letter is not a legal contract itself, but rather summarizes the main points of SSI's liability under its agreement with Clinical Research Centre, University of Surrey, Guildford, United Kingdom.

Yours sincerely,

Statens Serum Institut
Ingrid Kromann
Head of the Department of Vaccine Development

SUSPECT ADVERSE REACTION REPORT

I. REACTION INFORMATION

1. Subject initials / number	1a. Country	2. Date of birth Day | Month | Year	2a. Age Years	3. Sex F/M	4-6 Reaction onset Day | Month | Year	8-12. Check all appropriate to adverse reaction
/	Great Britain					☐ Patient died
7+13. Describe reaction(s) and identify reactions that are considered serious. Comment on relatedness and expectedness, intensity, outcome, whether vaccinations were discontinued.						☐ Involved or prolonged inpatient hospitalisation<br>☐ Involved persistent or significant disability or incapacity<br>☐ Life threatening<br>☐ Other medically important condition

II. SUSPECT DRUG(S) INFORMATION

14. Suspect drug(s) name(s) and batch number(s)		20. Did reaction abate after stopping drug?
15. Daily dose given of suspect drug(s)<br>1 test dose	16. Route(s) and sites of administration	☐ yes ☐ no ☒ NA
17. Indication(s) for use<br>**Diagnosis of Tuberculosis**		21. Did reaction reappear after reintroduction?
18. Date(s) of administration of suspect drug(s)	19. Therapy duration<br>N/A	☐ yes ☐ no ☒ NA

III. CONCOMITANT DRUG(S) AND HISTORY

22. List relevant past drug history and concomitant drug(s) and dates of administration

23. Other relevant medical history and concurrent conditions.

IV. MANUFACTURER INFORMATION

24a. Name and address of manufacturer
Statens Serum Institut, Artillerivej 5, DK-2300 Copenhagen S, Denmark

24b. Manufacturer Control No.	24d. Report source<br>☒ study ☐ literature<br>☐ health professional	Date and signature of initial reporter:<br><br>Print name of investigator:
24c. Date received by manufacturer		
Date of this report	25a. Report type<br>☐ initial ☐ follow-up	Date and signature of reporter of this report:<br><br>Print name of reporter:

Use this page if you have further comments or if additional space is needed for any of the fields of the CIOMS FORM. Please state No. of the field you continue on this page.

Field No.	Comments or continuation of text
	This field should always be complete<br>Subject initials:<br>Subject number:

CHARTIS

CERTIFICATE OF CLINICAL TRIAL LIABILITY INSURANCE

**INSURER:**	Chartis Insurance UK Limited
**POLICY NUMBER:**	20001156
**PROMOTER:**	Statens Serum Institut<br>5 Artillerivej<br>DK-2300 Copenhagen S, Denmark
**TRIAL REFERENCE:**	TESEC-03
**TRIAL TITLE:**	A phase IIa specificity trial of the diagnostic agent C-Tb, when given intradermally by the Mantoux technique to healthy volunteers previously vaccinated with BCG.
**LIMIT OF INDEMNITY:**	GBP5,000,000
**COUNTRY OF TRIAL:**	UK
**NUMBER OF PATIENTS:**	150
**NUMBER OF SITES:**	2
**PERIOD OF TRIAL:**	1st January 2011 to 31st December 2011

Terms and Conditions:
Such coverage being subject to the terms, conditions, limitations and exceptions of the Master Policy number 20001156.

This document is furnished as a matter of information only and does not modify the contract of insurance between the Insurer and the Insured.

SIGNED FOR AND ON BEHALF OF THE INSURER

Chartis Insurance UK Limited CHARTIS

CHARTIS INSURANCE UK LIMITED

This insurance is underwritten by Chartis Insurance UK Limited which is authorised and regulated by the Financial Services Authority (FSA number 202628). Chartis Insurance UK Limited is a member of the Association of British Insurers. Registered in England: company number 1486260. Registered address: The Chartis Building, 58 Fenchurch Street, London, EC3M 4AB.
